# Trends in the Prevalence of Common Retinal and Optic Nerve Diseases in China: An Artificial Intelligence Based National Screening

**DOI:** 10.1167/tvst.13.4.28

**Published:** 2024-04-22

**Authors:** Ruiheng Zhang, Li Dong, Xuefei Fu, Lin Hua, Wenda Zhou, Heyan Li, Haotian Wu, Chuyao Yu, Yitong Li, Xuhan Shi, Yangjie Ou, Bing Zhang, Bin Wang, Zhiqiang Ma, Yuan Luo, Meng Yang, Xiangang Chang, Zhaohui Wang, Wenbin Wei

**Affiliations:** 1 Beijing Tongren Eye Center, Beijing Key Laboratory of Intraocular Tumor Diagnosis and Treatment, Beijing Ophthalmology & Visual Sciences Key Lab, Medical Artificial Intelligence Research and Verification Key Laboratory of the Ministry of Industry and Information Technology, Beijing Tongren Hospital, Capital Medical University, Beijing, China; 2 Beijing Airdoc Technology Co., Ltd., Beijing, China; 3 School of Biomedical Engineering, Capital Medical University, Beijing, China; 4 iKang Guobin Healthcare Group Co., Ltd, Beijing, China

**Keywords:** artificial intelligence, screening, retinal and optic nerve diseases

## Abstract

**Purpose:**

Retinal and optic nerve diseases have become the primary cause of irreversible vision loss and blindness. However, there is still a lack of thorough evaluation regarding their prevalence in China.

**Methods:**

This artificial intelligence-based national screening study applied a previously developed deep learning algorithm, named the Retinal Artificial Intelligence Diagnosis System (RAIDS). De-identified personal medical records from January 2019 to December 2021 were extracted from 65 examination centers in 19 provinces of China. Crude prevalence and age-sex-adjusted prevalence were calculated by mapping to the standard population in the seventh national census.

**Results:**

In 2021, adjusted referral possible glaucoma (63.29, 95% confidence interval [CI] = 57.12–68.90 cases per 1000), epiretinal macular membrane (21.84, 95% CI = 15.64–29.22), age-related macular degeneration (13.93, 95% CI = 11.09–17.17), and diabetic retinopathy (11.33, 95% CI = 8.89–13.77) ranked the highest among 10 diseases. Female participants had significantly higher adjusted prevalence of pathologic myopia, yet a lower adjusted prevalence of diabetic retinopathy, referral possible glaucoma, and hypertensive retinopathy than male participants. From 2019 to 2021, the adjusted prevalence of retinal vein occlusion (0.99, 95% CI = 0.73–1.26 to 1.88, 95% CI = 1.42–2.44), macular hole (0.59, 95% CI = 0.41–0.82 to 1.12, 95% CI = 0.76–1.51), and hypertensive retinopathy (0.53, 95% CI = 0.40–0.67 to 0.77, 95% CI = 0.60–0.95) significantly increased. The prevalence of diabetic retinopathy in participants under 50 years old significant increased.

**Conclusions:**

Retinal and optic nerve diseases are an important public health concern in China. Further well-conceived epidemiological studies are required to validate the observed increased prevalence of diabetic retinopathy, hypertensive retinopathy, retinal vein occlusion, and macular hole nationwide.

**Translational Relevance:**

This artificial intelligence system can be a potential tool to monitor the prevalence of major retinal and optic nerve diseases over a wide geographic area.

## Introduction

Retinal and optic nerve diseases have become the primary cause of irreversible vision loss and blindness.^1^^,^^2^ In China, several population-based cohort studies reveal that pathologic myopia, glaucoma, age-related macular degeneration, and diabetic retinopathy are the most common causes of irreversible vision loss and blindness.^3^^–^^7^ In addition to the reduced quality of life, vision loss further increases the risk of overall mortality due to cardiovascular diseases,^8^ dementia,^9^ and depression.^10^ Vision loss leads to a severe disease burden on public health.

In 2021, the International Agency for the Prevention of Blindness (IAPB) implemented a new initiative, “2030 in Sight,” to achieve the goal that no one experiences unnecessary or preventable sight loss and everyone can achieve their full potential.^11^ As the most populous developing country in the world, China has the largest population at risk for vision loss. Yet, the population-based studies mentioned above were conducted within confined areas and age groups, which cannot fully reflect the prevalence of major retinal and optic nerve diseases in different regional, age, and gender groups. Additionally, some newly emerged challenges on vision loss warrant a national update survey, such as myopic maculopathy.^3^ Traditionally, the national epidemiological survey on retinal and optic nerve diseases is costly and not feasible, requiring oversampling of specific subgroups to increase the precision of estimates. In contrast, deep learning algorithms are shown to be cost-effective and could be used in large-scale screening. Previously, we developed a deep learning algorithm called the Retinal Artificial Intelligence Diagnosis System (RAIDS) to identify 10 retinal and optic nerve diseases.^12^ It was then implemented in real-world practice and showed superior or similar diagnostic performance to retinal experts.^12^ RAIDS-based screening could provide an update on the national prevalence of major retinal and optic nerve diseases.

In the current study, we exhibit the RAIDS-based screening of nearly 2 million participants attending annual physical examinations across 19 provinces of China, focusing on regional, age, and gender distribution of major retinal and optic nerve diseases.

## Methods

### Ethics, Consent, and Permissions

The Medical Ethics Committee of Beijing Tongren Hospital (TRECKY2018-056-GZ(2022)-07) and the Ethics Committee of the iKang Corporation (IKANG LLPJ-002) approved the study protocol. The Ethics Committee of the iKang Corporation approved and oversaw the de-identified, encrypted transmission, storage, and use of the images from all 65 examination centers of the iKang Guobin Health Physical Examination Management Group Co., Ltd. The Medical Ethics Committee of Beijing Tongren Hospital approved and oversaw the image process, statistical analysis, and manuscript drafting. All fundus images were de-identified before the analysis. Oral informed consent was obtained from all participants at the outset of the physical examination and has been formally documented. The Ethics Committee of iKang Corporation exempted written informed consent in the present study as the data retrospectively used in this study were de-identified beforehand. The study was registered on ClinicalTrials (https://clinicaltrials.gov/; NCT04678375, and NCT04592068).

### Study Population and Baseline Data

De-identified personal medical records from January 2019 to December 2021 were extracted from 65 examination centers of the iKang Guobin Health Physical Examination Management Group Co., Ltd. (Supplementary Table S1, Supplementary Fig. S1). All participants enrolled in an annual physical examination at iKang Guobin centers, which included physical measurements (body weight, height, waist circumference, and blood pressure), a fasting blood test (fasting plasma glucose, HbA1c, total cholesterol, low-density lipoprotein, high-density lipoprotein, and serum creatinine), and a medical history questionnaire. Diabetes status was ascertained by medication usage or meeting criteria according to the American Diabetes Association standard.^13^ Arterial hypertension was ascertained by medication usage, or an average systolic blood pressure of at least 140 mm Hg or an average diastolic blood pressure of at least 90 mm Hg.^14^ The inclusion criteria included: (1) participants who underwent a fundus photograph examination; and (2) gradable fundus images from both eyes.

### Deployment of the RAIDS and Outcome Ascertainment

The development and performance of RAIDS has been extensively illustrated in a previous study.^12^ In brief, the overall architecture of the multi-task convolutional neural network (CNN) included a first part containing a model for the macula and optic disc region and a second part consisting of a model for multi-task learning for the retinal disease classification. In real-world validation, 208,758 images from 110,784 participants and 10,084 images from the Beijing Eye Study and Kailuan Eye Study were used for external validation. For diagnosing 10 retinal and optic nerve diseases, the accuracy of RAIDS ranged from 0.982 to 1.000, which is comparable to most retinal experts.^12^

In the present study, RAIDS was deployed on a cloud service and connected to the fundus cameras through the internet. Using nonmydriatic 45-degree fundus cameras (see Supplementary Table S1), trained operators took binocular fundus photography of participants and stored them at local computers. Operators were then asked to identify non-gradable images due to reasons such as blur or defocus, and took fundus photography repeatedly. If gradable images could not be obtained eventually, the participants were excluded from RAIDS diagnosis and referred to the local hospital.^12^ RAIDS was used to automatically generate a report on 10 retinal and optic nerve diseases, including diabetic retinopathy, age-related macular degeneration, referral possible glaucoma, pathological myopia, retinal vein occlusion, macula hole, epiretinal macular membrane, hypertensive retinopathy, myelinated fibers, and retinitis pigmentosa. Once the RAIDS-based diagnosis was made, the fundus images were simultaneously sent to all participants, and subsequent referral options were given to a retinal expert at the local hospital.

### Statistical Analysis

Based on the seventh national census of China in 2020 (http://www.stats.gov.cn/tjsj/pcsj/rkpc/7rp/indexch.htm, accessed on June 15, 2022), a standard population by age and sex group was generated (Supplementary Table S2). The standard population refers to the age and sex distributions derived from a census. Consequently, both the crude prevalence and age-sex-adjusted prevalence were calculated by mapping the data to the standard population from the seventh national census. Mapping to the standard Chinese population served to adjust for the differences in age and sex composition between the included participants and the general Chinese population. N-out-of-N bootstrapping with 2000 replicates was used to estimate 95% confidence intervals (95% CI) of the crude prevalence and adjusted prevalence. All statistical analysis was performed using R Statistical Software (version 4.1.1; R Foundation for Statistical Computing, Vienna, Austria).

## Results

A total of 1,904,927 participants enrolled in the annual physical examination. The numbers of participants under 30 years, 30 to 39 years, 40 to 49 years, 50 to 59 years, and above 60 years were 15.6%, 33.8%, 18.8%, 18.9%, and 12.9%, respectively (Table 1). The prevalence of diabetes and arterial hypertension were higher in the older age group.

**Table 1.** Baseline Characteristics of Participants

	Age group				
	29 and below	30-39	40–49	50–59	60 and above
Number of participants	298,055	644,810	357,257	360,630	244,175
Male (%)	142,145 (47.69)	340,767 (52.85)	192,478 (53.88)	182,013 (50.47)	123,256 (50.48)
Female (%)	155,910 (52.31)	304,043 (47.15)	164,779 (46.12)	178,617 (49.53)	120,919 (49.52)
Systolic blood pressure (SD)	115.44 (13.27)	116.11 (14.13)	120.26 (16.06)	128.07 (17.97)	136.9 (19.05)
Diastolic blood pressure (SD)	69.53 (9.27)	71.1 (10.52)	74.75 (12.14)	78.21 (12)	77.81 (11.29)
BMI (SD)	22.59 (7.1)	23.72 (14.32)	24.5 (3.69)	24.86 (3.35)	24.79 (3.46)
Waist circumference (SD)	76.92 (11.78)	81.45 (11.52)	83.49 (10.77)	85.23 (12.77)	86.48 (10.03)
Fasting plasma glucose (SD)	5.01 (0.66)	5.19 (0.87)	5.46 (1.23)	5.8 (1.52)	6.08 (1.62)
HbA1c, % (SD)	5.32 (0.43)	5.43 (0.55)	5.6 (0.73)	5.86 (0.92)	6.11 (1.01)
Total cholesterol (SD)	4.6 (0.85)	4.82 (1.16)	5.04 (0.94)	5.27 (1.01)	5.25 (1.08)
Low-density lipoprotein (SD)	2.65 (0.72)	2.82 (0.88)	2.96 (0.77)	3.1 (0.8)	3.1 (1.22)
High-density lipoprotein (SD)	1.48 (0.3)	1.45 (0.35)	1.46 (0.38)	1.48 (0.32)	1.49 (0.32)
Serum creatinine (SD)	66.12 (14.12)	66.28 (15.21)	66.78 (16.84)	66.04 (17.93)	67.61 (18.94)
Diabetes, %	0.45	1.53	5.04	11.53	19.78
Hypertension, %	4.23	6.64	15.59	32.11	51.56

BMI, body mass index.

### Prevalence of Retinal and Optic Nerve Diseases in 2021

The prevalence of 10 major retinal and optic nerve diseases are listed in Table 2. In 2021, adjusted referral possible glaucoma (63.29, 95% CI = 57.12–68.90 cases per 1000) had the highest age and sex-adjusted prevalence among 10 diseases. Adjusted prevalence of epiretinal macular membrane (21.84, 95% CI = 15.64–29.22 cases per 1000), age-related macular degeneration (13.93, 95% CI = 11.09–17.17 cases per 1000), and diabetic retinopathy (11.33, 95% CI = 8.89–13.77 cases per 1000) ranked after referral possible glaucoma. For retinal pigmentosa, it was estimated that the age and sex-adjusted prevalence was 0.35 (95% CI = 0.26–0.44) per 1000 population.

**Table 2.** Prevalence of Retinal and Optic Nerve Diseases in 2019 to 2021

	Prevalence 2019		Prevalence 2020		Prevalence 2021		Percentage Change	
	*n* = 659,026		*n* = 874,902		*n* = 370,999		2019–2021	
	Crude	Adjusted	Crude	Adjusted	Crude	Adjusted	Crude	Adjusted
Diabetic retinopathy	9.60 (9.36–9.83)	10.99 (8.60–13.57)	10.30 (10.09–10.52)	11.36 (8.88–13.81)	10.28 (9.96–10.61)	11.33 (8.89–13.77)	7.08%	3.09%
Age-related macular degeneration	11.62 (11.36–11.88)	14.22 (11.25–17.43)	11.14 (10.92–11.36)	13.28 (10.39–16.43)	11.19 (10.85–11.53)	13.93 (11.09–17.17)	−3.70%	−2.04%
Referral possible glaucoma	74.22 (73.59–74.86)	76.25 (67.83–85.37)	65.67 (65.15–66.19)	66.75 (58.70–76.53)	65.43 (64.64–66.23)	63.29 (57.12–68.90)	−11.84%	−17.00%
Pathological myopia	5.22 (5.05–5.40)	6.46 (5.27–7.73)	5.43 (5.28–5.58)	7.09 (5.62–8.77)	5.53 (5.29–5.77)	7.35 (5.92–9.18)	5.94%	13.78%
Retinal vein occlusion	0.73 (0.67–0.80)	0.99 (0.73–1.26)	1.13 (1.06–1.20)	1.39 (1.05–1.78)	1.57 (1.44–1.70)	1.88 (1.42–2.44)	115.07%	89.90%
Macular hole	0.44 (0.39–0.50)	0.59 (0.41–0.82)	0.74 (0.68–0.80)	0.96 (0.69–1.26)	0.86 (0.77–0.96)	1.12 (0.76–1.51)	95.45%	89.83%
Epiretinal macular membrane	15.13 (14.84–15.43)	22.36 (15.82–29.42)	15.70 (15.44–15.96)	21.74 (15.71–28.85)	16.59 (16.18–17.01)	21.84 (15.64–29.22)	9.65%	−2.33%
Hypertensive retinopathy	0.45 (0.40–0.51)	0.53 (0.40–0.67)	0.54 (0.49–0.59)	0.55 (0.43–0.67)	0.80 (0.71–0.89)	0.77 (0.60–0.95)	77.78%	45.28%
Myelinated fibers	5.97 (5.78–6.16)	6.22 (4.81–8.42)	6.03 (5.86–6.19)	5.33 (4.69–6.00)	6.14 (5.89–6.40)	5.65 (4.89–6.42)	2.85%	−9.16%
Retinitis pigmentosa	0.35 (0.31–0.40)	0.32 (0.25–0.37)	0.36 (0.32–0.41)	0.32 (0.26–0.38)	0.39 (0.33–0.46)	0.35 (0.26–0.44)	11.43%	9.38%

Crude, crude prevalence per 1000 participants; Adjusted, age and sex-adjusted prevalence per 1000 standard population. Data were expressed as the estimate (95% confidence interval).

**Figure 1.**
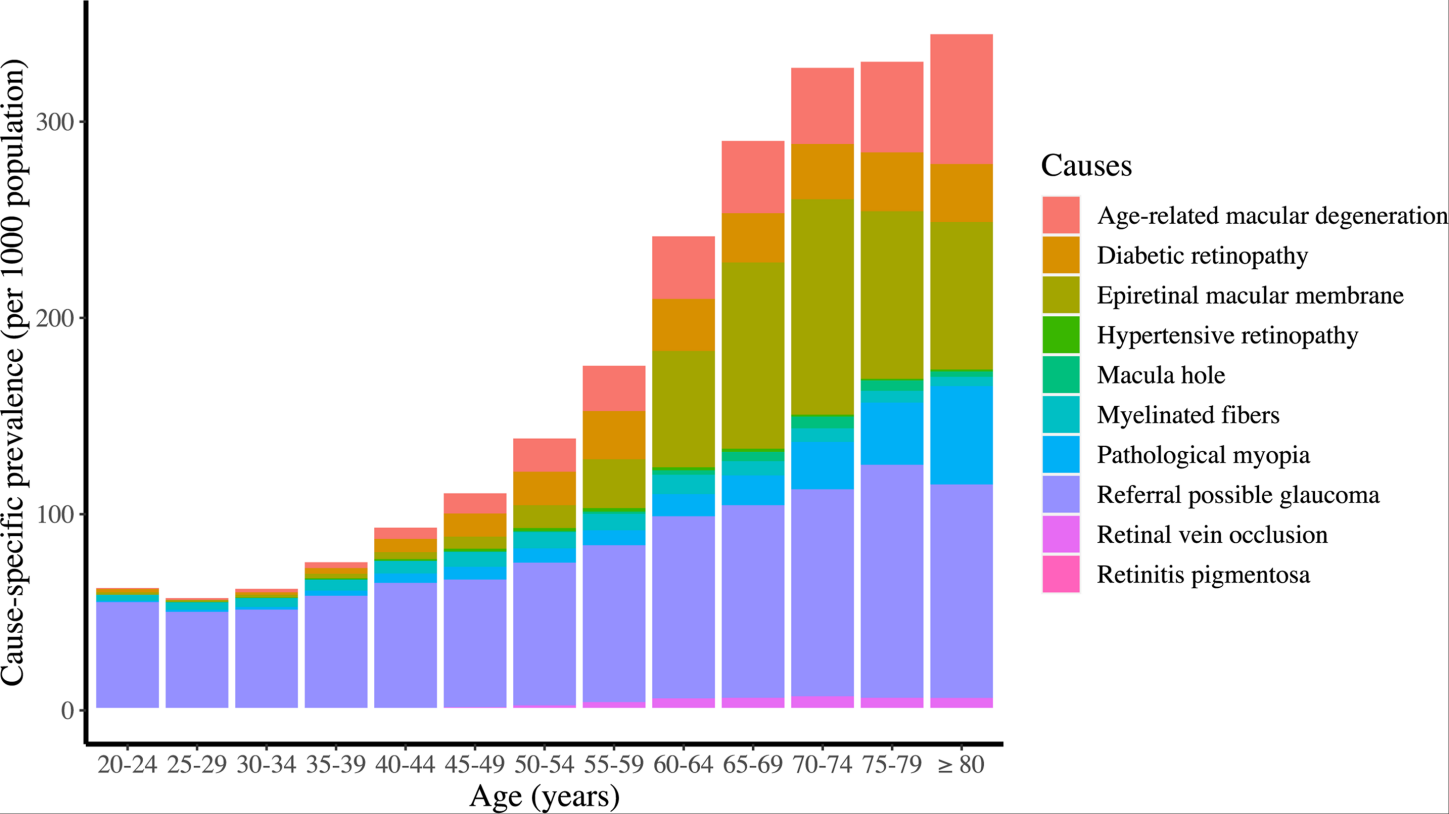
Association between age and 10 major retinal and optic nerve diseases in 2021.

There was a strong association between age and retinal and optic nerve diseases (Fig. 1, Supplementary Tables S2–S13). The overall prevalence began to rise at age 35 to 39 years. Among participants younger than 50 years, referral possible glaucoma was the leading constitution for retinal and optic nerve diseases. In contrast, the prevalence of age-related macular degeneration, diabetic retinopathy, epiretinal macula membrane, and pathologic myopia have significantly increased after the age of 50 years. In the working-age population (aged 15 to 64 years), referral possible glaucoma, age-related macular degeneration, diabetic retinopathy, and epiretinal macula membrane were the most prevalent retinal and optic nerve diseases (Supplementary Table S14).

**Figure 2.**
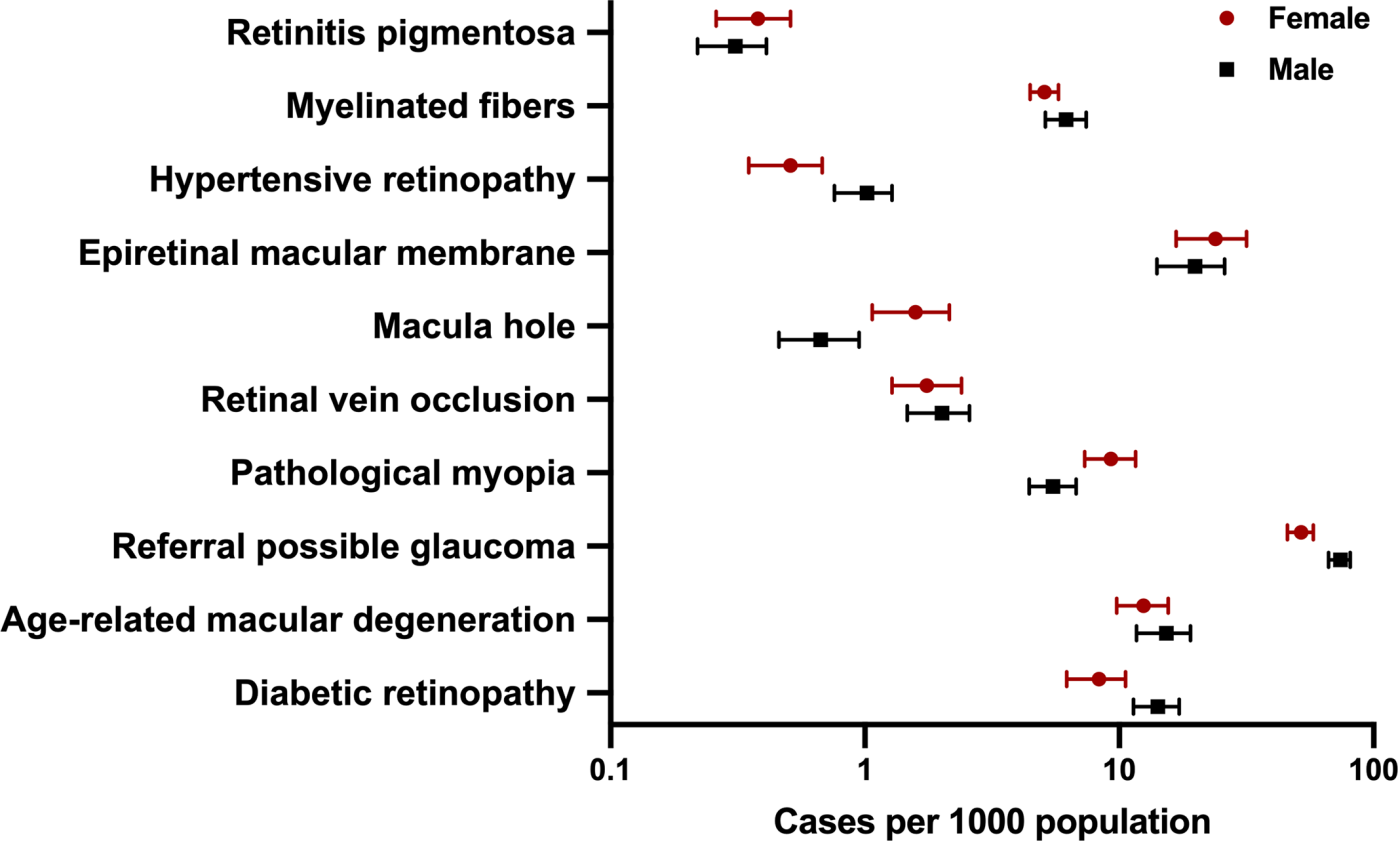
Sex difference of 10 major retinal and optic nerve diseases in 2021.

Sex differences were also observed in several retinal and optic nerve diseases (Fig. 2, Supplementary Tables S15, S16). In 2021, female participants had a significantly higher adjusted prevalence of pathologic myopia (9.29, 95% CI = 7.31–11.61 for female participants and 5.49, 95% CI = 4.43–6.78 for male participants, cases per 1000), yet there was a lower prevalence of adjusted diabetic retinopathy (8.33, 95% CI = 6.21–10.59 for female participants and 14.19, 95% CI = 11.37–17.21 for male participants, cases per 1000), referral possible glaucoma (51.90, 95% CI = 45.88–57.91 for female participants and 74.15, 95% CI = 66.52–81.03 for male participants, cases per 1000), and hypertensive retinopathy (0.51, 95% CI = 0.35–0.68 for female participants and 1.02, 95% CI = 0.76–1.28 for male participants, cases per 1000). There were no obvious sex differences among other retinal and optic nerve diseases.

Among RAIDS-equipped healthcare centers in 19 provinces of China, 17 provinces completed more than 10,000 participants in 17 provinces over a 3-year screening period. In 2021, regional differences were detected among 17 provinces (Supplementary Tables S17–S33). Yet, no apparent geographic trend was observed, except for hypertensive retinopathy and retinal vein occlusion, which exhibited an increasing trend from southeast to northwest (Supplementary Figs. S2–S11).

### Trend Analyses

From 2019 to 2021, the age- and sex-adjusted prevalence of retinal vein occlusion (0.99, 95% CI = 0.73–1.26 to 1.88, 95% CI = 1.42–2.44 cases per 1000, percentage change: 89.90%), macular hole (0.59, 95% CI = 0.41–0.82 to 1.12, 95% CI = 0.76–1.51 cases per 1000, percentage change: 89.83%) and hypertensive retinopathy (0.53, 95% CI = 0.40–0.67 to 0.77, 95% CI = 0.60–0.95 cases per 1000, percentage change: 42.28%) significantly increased (see Table 2). The increased prevalence of these retinal and optic nerve diseases could be found in almost all age groups, both sexes and almost all provinces (see Supplementary Tables S2–S33). However, the magnitude of increased prevalence was unevenly distributed among the age groups, especially for retinal vein occlusion and hypertensive retinopathy. For retinal vein occlusion, the adjusted prevalence increased by 366.67% (0.03, 95% CI = 0.01–0.06 to 0.14, 95% CI = 0.05–0.28 cases per 1000), 211.11% (0.09, 95% CI = 0.02–0.17 to 0.28, 95% CI = 0.09–0.46 cases per 1000), 93.94% (0.33, 95% CI = 0.29–0.38 to 0.64, 95% CI = 0.51–0.81 cases per 1000), and 136.36% (0.55, 95% CI = 0.34–0.79 to 1.30, 95% CI = 0.79–1.80 cases per 1000) in the 30 to 34 years, 35 to 39 years, 40 to 44 years, and 45 to 49 years age groups, respectively, which was higher than 89.90% in the overall population. Similarly, the adjusted prevalence of hypertensive retinopathy has also increased by 300% (0.02, 95% CI = 0.00–0.08 to 0.08, 95% CI = 0.02–0.14 cases per 1000), 271.43% (0.14, 95% CI = 0.08–0.19 to 0.52, 95% CI = 0.34–0.82 cases per 1000), 120.59% (0.34, 95% CI = 0.17–0.50 to 0.75, 95% CI = 0.52–0.98 cases per 1000), and 249.12% (0.34, 95% CI = 0.21–0.51 to 1.34, 95% CI = 0.94–1.78 cases per 1000) in the 25 to 29 years, 35 to 39 years, 40 to 44 years, and 45 to 49 years age groups, respectively, which was higher than 89.83% in the overall population (see Supplementary Tables S2–S6).

We did not observe any significant changes in the general prevalence of the other seven retinal and optic nerve diseases (see Table 2). However, we also found a striking increase in the prevalence of diabetic retinopathy in young age groups. From 2019 to 2021, the adjusted prevalence of diabetic retinopathy significantly increased by 270.83% (0.24, 95% CI = 0.00–0.51 to 1.13, 95% CI = 0.03–3.31 cases per 1000), 69.70% (0.33, 95% CI = 0.18–0.44 to 0.56, 95% CI = 0.36–0.76 cases per 1000), and 41.94% (0.93, 95% CI = 0.59–1.29 to 1.32, 95% CI = 0.77–1.99 cases per 1000) in the 20 to 24 years, 25 to 29 years, and 30 to 34 years age groups, respectively. In contrast, no significant changes were observed in older age groups (see Supplementary Tables S2–S13).

## Discussion

In the present study, RAIDS was deployed in real-world healthcare centers and screened nearly 2 million participants attending annual physical examinations across 19 provinces of China. We found that referral possible glaucoma, epiretinal macular membrane, age-related macular degeneration, and diabetic retinopathy were the most prevalent retinal and optic nerve diseases in China. The present study showed associations between age as well as gender with the prevalence of retinal and optic nerve diseases. The present study also highlights the strikingly increased prevalence of retinal vein occlusion, diabetic retinopathy, and hypertensive retinopathy, especially in the age groups below 50 years.

The accurate diagnosis of glaucoma on one visit is challenging, especially based solely on fundus photography. In the present study, we estimated that referral possible glaucoma has the highest prevalence in China. In 2021, 63.29 of 1000 (95% CI = 57.12–68.90) participants were identified as susceptible to glaucoma with RAIDS. Enlarged cup-to-disc ratio, focal thinning or notching of the neuro-retinal rim, optic disc hemorrhages, and localized retinal nerve fiber layer defects are the primary features of fundus changes of glaucoma.^15^ Thus, the prevalence of referral possible glaucoma reflects the detection rate of these fundus changes. Based on over 5000 participants in the US National Health and Nutrition Examination Survey, about 6% of participants aged ≥40 years had an enlarged cup-to-disc ratio (≥0.6).^16^ Similarly, in the Beijing Eye Study, 4.6% of participants had at baseline and 3.4% of participants developed localized retinal nerve fiber layer defects during the 10-year follow-up.^17^ In contrast, only 3.6% of Beijing Eye Study population was diagnosed with glaucoma.^18^ These studies highlight the limited specificity of the fundus photography in glaucoma diagnosis,^19^^,^^20^ and accurate diagnosis of glaucoma relies on long-term follow-up. For now, population screening for glaucoma is borderline cost-effective in both urban and rural China.^21^ Artificial intelligence-based screening, especially co-screening of multi-retinal and optic disc diseases on one fundus photography, could largely reduce screening costs and make population screening for glaucoma cost-effective.^22^

Apart from glaucoma, we found that epiretinal macular membrane, age-related macular degeneration, and diabetic retinopathy rank after referral possible glaucoma. In particular, we also estimated the prevalence of retinitis pigmentosa is 0.39 of 1000 (95% CI = 0.33–0.46) in 2021, which is slightly higher than a large generic eye disease screening conducted in 1986 (0.26/1000).^23^ According to the Singapore Epidemiology of Eye Diseases, it is estimated that 0.09% of Chinese participants between 40 and 80 years of age have retinitis pigmentosa.^24^ The present study showed similar prevalence in these age groups (see Supplementary Tables S5–S12).

Based on the large population and artificial intelligence-based screening, we depicted the prevalence of these diseases in different age, sex, and regional groups. We found a geographic increase in hypertensive retinopathy and retinal vein occlusion from southeast to northwest. This geographic trend matches the sodium intake of Chinese, based on a national nutrition survey. It is well known that high sodium intake is the primary cause of hypertension, which further increases the risk of retinal vein occlusion.^25^ By recording diet and urinary sodium excretion, extensive studies have found a geographic increase in sodium intake from southeast to northwest in China.^26^^,^^27^ Thus, such geographic variance in hypertensive retinopathy and retinal vein occlusion prevalence might be caused by diet habits. Besides, the prevalence of major retinal and optic nerve diseases is closely correlated with age and exhibited sex difference.^2^ The age- and sex-specific prevalence is in line with estimates from the Global Burden of Disease (GBD) study.^28^ Compared to the GBD study, the present study covers a broader range of diseases.

Through RAIDS-based screening, we can track the yearly changes in prevalence. This cross-sectional study delineates the trend of 10 major retinal and optic disc diseases during 3 consecutive years, including the period before and during the coronavirus disease 2019 (COVID-19) restriction measures in China. According to the National Health Commission of China, 87,071 and 15,243 cases were reported in 2020 and 2021, respectively. By the end of 2021, the infection rate of COVID-19 in mainland China was about 0.007%, which largely excludes the effects of COVID-19 infection. We found a significant increase in retinal vein occlusion, hypertensive retinopathy, and macular holes from 2019 to 2021. Arterial hypertension is the most common cardiovascular risk factor for retinal vein occlusion,^25^ and the cause of hypertensive retinopathy. During the past 3 years, the rise in blood pressure was observed in several studies,^29^^,^^30^ which could partially explain these changes from 2019 to 2021. Meanwhile, the prevalence of macular holes also significantly increased during 2019 to 2021. This might be caused by delayed diagnosis, surgery, and increased waiting times during the COVID-19 pandemic.^31^

The present study further highlights the increased prevalence of diabetic retinopathy, retinal vein occlusion, and hypertensive retinopathy in young age groups. According to epidemiological surveys in China, the prevalence of diabetes in the 20 to 39 years age group was 3.2% according to the 2008 national survey and 5.9% according to the 2013 national survey.^32^ A similar trend was also found by China National Nutrition and Health Survey.^33^^,^^34^ The present study highlights the target organ damage of diabetes and hypertension among young age groups.

This study has some strengths. We used a multi-center, artificial intelligence-based screening technique to explore the prevalence, dynamic changes, age-, sex-, and regional differences of 10 retinal and optic nerve diseases. However, some limitations should be mentioned. First of all, we did not obtain sufficient and unbiased data on visual acuity. The missing data on visual acuity limited further exploration of the visual loss burden. In health examinations, it is difficult to carry out a best-corrected visual acuity examination for all participants. Thus, subsequent studies are needed to fully understand the impact of retinal and optic nerve diseases on vision loss. Second, artificial intelligence-based screening may misclassify some cases. In a previous study, we proved that RAIDS achieved superior/noninferior diagnostic accuracy and sensitivity compared to retinal experts and general ophthalmologists. However, it demonstrated lower specificity in diagnosing macular hole and epiretinal macular membrane compared to human ophthalmologists. Consequently, the prevalence estimates for these retinal diseases should be interpreted with caution. Third, the present study only included 19 out of 34 provinces, municipalities, and autonomous regions. Data from these areas can help to better understand the rational and ethnic differences in major retinal and optic nerve diseases. However, data from the majority of Middle and West China are still lacking. Fourth, the duration of the present study was short, with a 2-year interval, which might not be sufficient to accurately reflect long-term changes in disease. Given the short duration of the study, such significant changes in prevalence are unexpected and warrant a cautious interpretation, as in the cases of hypertensive retinopathy and retinal vein occlusion. Further observation is needed to validate the long-term trend of increased prevalence of these diseases and to exclude potential influence from other factors or methodological aspects in the future. Fifth, because the current study was conducted in the physical examination institution, the results might be biased by confounding factors, such as socioeconomic status and occupation. Additionally, the inclusion criteria of only considering patients with both eyes gradable could potentially introduce a selection bias, affecting the generalizability of the results.

In conclusion, retinal and optic nerve diseases are a significant public health concern in China. Further well-conceived epidemiological studies are required to validate the observed increased prevalence of diabetic retinopathy, hypertensive retinopathy, retinal vein occlusion, and macular hole nationwide.

## Supplementary Material

Supplement 1
