# Supplementary material for: Trends in the Prevalence of Common Retinal and Optic Nerve Diseases in China: An Artificial Intelligence Based National Screening
Source: Transl Vis Sci Technol. 2024 Apr 22;13(4):28. doi: 10.1167/tvst.13.4.28 (PMC11044835; doi:10.1167/tvst.13.4.28)

Table of Contents

**Section S1: Supplementary eTables**

[Table S1. List of the location of each health screening centers and the digital retinal cameras used in each center. 3](#_Toc157463922)

[Table S2. Standard population based on the seventh national census of China 6](#_Toc157463923)

[Table S3. Prevalence of retinal and optic nerve diseases in aged 20 to 24 participants. 11](#_Toc157463924)

[Table S4. Prevalence of retinal and optic nerve diseases in aged 25 to 29 participants. 11](#_Toc157463925)

[Table S5. Prevalence of retinal and optic nerve diseases in aged 30 to 34 participants. 12](#_Toc157463926)

[Table S6. Prevalence of retinal and optic nerve diseases in aged 35 to 39 participants. 13](#_Toc157463927)

[Table S7. Prevalence of retinal and optic nerve diseases in aged 40 to 44 participants. 14](#_Toc157463928)

[Table S8. Prevalence of retinal and optic nerve diseases in aged 45 to 49 participants. 14](#_Toc157463929)

[Table S9. Prevalence of retinal and optic nerve diseases in aged 50 to 54 participants. 15](#_Toc157463930)

[Table S10. Prevalence of retinal and optic nerve diseases in aged 55 to 59 participants. 16](#_Toc157463931)

[Table S11. Prevalence of retinal and optic nerve diseases in aged 60 to 64 participants. 17](#_Toc157463932)

[Table S12. Prevalence of retinal and optic nerve diseases in aged 65 to 69 participants. 18](#_Toc157463933)

[Table S13. Prevalence of retinal and optic nerve diseases in aged 70 to 74 participants. 18](#_Toc157463934)

[Table S14. Prevalence of retinal and optic nerve diseases in aged 75 to 79 participants. 19](#_Toc157463935)

[Table S15. Prevalence of retinal and optic nerve diseases in aged 80 or above participants. 20](#_Toc157463936)

[Table S16. Prevalence of retinal and optic nerve diseases in working population (aged 15-64). 21](#_Toc157463937)

[Table S17. Prevalence of retinal and optic nerve diseases in female participants. 22](#_Toc157463938)

[Table S18. Prevalence of retinal and optic nerve diseases in male participants. 22](#_Toc157463939)

[Table S19. Prevalence of retinal and optic nerve diseases in Anhui province. 23](#_Toc157463940)

[Table S20. Prevalence of retinal and optic nerve diseases in Beijing. 24](#_Toc157463941)

[Table S21. Prevalence of retinal and optic nerve diseases in Chongqing. 25](#_Toc157463942)

[Table S22. Prevalence of retinal and optic nerve diseases in Fujian. 26](#_Toc157463943)

[Table S23. Prevalence of retinal and optic nerve diseases in Guangdong. 26](#_Toc157463944)

[Table S24. Prevalence of retinal and optic nerve diseases in Guizhou. 27](#_Toc157463945)

[Table S25. Prevalence of retinal and optic nerve diseases in Hubei 28](#_Toc157463946)

[Table S26. Prevalence of retinal and optic nerve diseases in Hunan. 29](#_Toc157463947)

[Table S27. Prevalence of retinal and optic nerve diseases in aged Jiangsu 30](#_Toc157463948)

[Table S28. Prevalence of retinal and optic nerve diseases in Liaoning. 30](#_Toc157463949)

[Table S29. Prevalence of retinal and optic nerve diseases in Ningxia. 31](#_Toc157463950)

[Table S30. Prevalence of retinal and optic nerve diseases in Shannxi. 32](#_Toc157463951)

[Table S31. Prevalence of retinal and optic nerve diseases in Shandong. 33](#_Toc157463952)

[Table S32. Prevalence of retinal and optic nerve diseases in Shanghai. 34](#_Toc157463953)

[Table S33. Prevalence of retinal and optic nerve diseases in Sichuan. 34](#_Toc157463954)

[Table S34. Prevalence of retinal and optic nerve diseases Tianjin 35](#_Toc157463955)

[Table S35. Prevalence of retinal and optic nerve diseases in Zhejiang 36](#_Toc157463956)

[Figure S1. Distribution of participants in 2019-2021. 38](#_Toc157463957)

[Figure S2. Prevalence of diabetic retinopathy in 2021 (age and sex-adjusted prevalence per 1000 standard population). 39](#_Toc157463958)

[Figure S3. Prevalence of age-related macular degeneration in 2021 (age and sex-adjusted prevalence per 1000 standard population). 40](#_Toc157463959)

[Figure S4. Prevalence of referral possible glaucoma in 2021 (age and sex-adjusted prevalence per 1000 standard population. 41](#_Toc157463960)

[Figure S5. Prevalence of pathological myopia in 2021(age and sex-adjusted prevalence per 1000 standard population). 42](#_Toc157463961)

[Figure S6. Prevalence of retinal vein occlusion in 2021(age and sex-adjusted prevalence per 1000 standard population). 43](#_Toc157463962)

[Figure S7. Prevalence of macula hole in 2021 (age and sex-adjusted prevalence per 1000 standard population). 44](#_Toc157463963)

[Figure S8. Prevalence of epiretinal macular membrane in 2021 (age and sex-adjusted prevalence per 1000 standard population). 45](#_Toc157463964)

[Figure S9. Prevalence of hypertensive retinopathy in 2021 (age and sex-adjusted prevalence per 1000 standard population). 46](#_Toc157463965)

[Figure S10. Prevalence of myelinated fibers in 2021 (age and sex-adjusted prevalence per 1000 standard population). 47](#_Toc157463966)

[Figure S11. Prevalence of retinitis pigmentosa in 2021 48](#_Toc157463967)

## Table S1. List of the location of each health screening centers and the digital retinal cameras used in each center.

| **Province** | **Camera** | **iKang Corporation public health screening centers (City)** |
| --- | --- | --- |
| Shanghai | TRC-NW200 | Shanghai Yuanhua Medical Centre of iKang Guobin Healthcare Group (Shanghai, China) |
| Shanghai | CR-2 AF | Shanghai Jun'an Medical Centre of iKang Healthcare Group (Shanghai, China) |
| Shanghai | CR-2 AF | Shanghai Wangzu Medical Centre of iKang Guobin Healthcare Group (Shanghai, China) |
| Shanghai | CR-2 AF | Shanghai Zhiwei Medical Centre of iKang Guobin Healthcare Group (Shanghai, China) |
| Shanghai | CR-2 AF | Shanghai Yipin Medical Centre of iKang Guobin Healthcare Group (Shanghai, China) |
| Shanghai | CR-2 AF | Shanghai Fukang Medical Centre of iKang Guobin Healthcare Group (Shanghai, China) |
| Shanghai | CR-2 AF | Shanghai Binming Medical Centre of iKang Guobin Healthcare Group (Shanghai, China) |
| Shanghai | CR-2 AF | Shanghai Waizhitan Medical Centre of iKang Guobin Healthcare Group (Shanghai, China) |
| Shanghai | CR-2 AF | Shanghai Renren Medical Centre of iKang Guobin Healthcare Group (Shanghai, China) |
| Anhui | CR-2 AF | Hefei Medical Centre of iKang Healthcare Group (Hefei, China) |
| Anhui | CR-2 AF | Wuhu Medical Centre of iKang Guobin Healthcare Group (Wuhu, China) |
| Jiangsu | CR-2 AF | Nanjing Junan Medical Centre of iKang Guobin Healthcare Group (Nanjing, China) |
| Jiangsu | CR-2 AF | Nanjing Xinjie Medical Centre of iKang Guobin Healthcare Group (Nanjing, China) |
| Jiangsu | CR-2 AF | Wuxi Medical Centre of iKang Healthcare Group (Wuxi, China) |
| Jiangsu | CR-2 AF | Hangzhou Wenhui Medical Centre of iKang Healthcare Group (Hangzhou, China) |
| Jiangsu | CR-2 AF | Hangzhou Zhuoyue Medical Centre of iKang Healthcare Group (Hangzhou, China) |
| Jiangsu | CR-2 AF | Hangzhou Junan Medical Centre of iKang Guobin Healthcare Group (Hangzhou, China) |
| Jiangsu | CR-2 AF | Suzhou Medical Centre of iKang Guobin Healthcare Group (Suzhou, China) |
| Jiangsu | CR-2 AF | Suzhou Zhuoyue Medical Centre of iKang Healthcare Group (Suzhou, China) |
| Jiangsu | CR-2 AF | Zhenjiang Wenguang Medical Centre of iKang Healthcare Group (Zhenjiang, China) |
| Jiangsu | CR-2 AF | Jiangyin LingangMedical Centre of iKang Healthcare Group (Jiangyin, China) |
| Zhejiang | CR-2 AF | Ningbo Haishu Medical Centre of iKang Guobin Healthcare Group (Ningbo, China) |
| Hubei | CR-2 AF | Yichang Zhuoyue Medical Centre of iKang Healthcare Group (Yichang, China) |
| Hubei | CR-2 AF | Wuhan Jindun Medical Centre of iKang Guobin Healthcare Group (Wuhan, China) |
| Hubei | CR-2 AF | Wuhan Zhuoyue Medical Centre of iKang Healthcare Group (Wuhan, China) |
| Jilin | CR-2 AF | Changchun Jianshe Medical Centre of iKang Guobin Healthcare Group (Changchun, China) |
| Tianjin | CR-2 AF | Tianjin Fenghui Medical Centre of iKang Guobin Healthcare Group (Tianjin, China) |
| Tianjin | CR-2 AF | Tianjin Dongrun Medical Centre of iKang Guobin Healthcare Group (Tianjin, China) |
| Tianjin | CR-2 AF | Tianjin Heping Medical Centre of iKang Healthcare Group (Tianjin, China) |
| Tianjin | CR-2 AF | Tianjin Hexi Medical Centre of iKang Guobin Healthcare Group (Tianjin, China) |
| Tianjin | CR-2 AF | Tianjin Yuecheng Medical Centre of iKang Guobin Healthcare Group (Tianjin, China) |
| Shandong | CR-2 AF | Weihai Ciming Medical Centre of iKang Guobin Healthcare Group (Weihai, China) |
| Shandong | CR-2 AF | Jinan Zhuoyue Medical Centre of iKang Healthcare Group (Jinan, China) |
| Shandong | CR-2 AF | Weifang Ciming Medical Centre of iKang Guobin Healthcare Group (Weifang, China) |
| Shandong | CR-2 AF | Yantai Medical Centre of iKang Healthcare Group (Yantai, China) |
| Shandong | CR-2 AF | Qingdao Zhuoyue Medical Centre of iKang Healthcare Group (Qingdao, China) |
| Liaoning | TRC-NW300 | Shenyang Jinai Medical Centre of iKang Guobin Healthcare Group (Shenyang, China) |
| Liaoning | CR-2 AF | Shenyang Shenhe Medical Centre of iKang Guobin Healthcare Group (Shenyang, China) |
| Beijing | CR-2 AF | Beijing Yayun Medical Centre of iKang Guobin Healthcare Group (Beijing, China) |
| Beijing | CR-2 AF | Beijing Xizhimen Medical Centre of iKang Guobin Healthcare Group (Beijing, China) |
| Shanxi | Nonmyd α-DⅢ | Xi'an Qujiang Medical Centre of iKang Guobin Healthcare Group (Xi'an, China) |
| Shanxi | Nonmyd α-DⅢ | Xi'an Weiyang Medical Centre of iKang Guobin Healthcare Group (Xi'an, China) |
| Shanxi | Nonmyd α-DⅢ | Xi'an Lianhu Medical Centre of iKang Healthcare Group (Xi'an, China) |
| Guangdong | CR-2 AF | Dongguan Songshan Medical Centre of iKang Healthcare Group (Dongguan, China) |
| Guangdong | CR-2 AF | Dongguan Caifu Medical Centre of iKang Guobin Healthcare Group (Dongguan, China) |
| Guangdong | CR-2 AF | Foshan Medical Centre of iKang Healthcare Group (Foshan, China) |
| Guangdong | CR-2 AF | Guangzhou Medical Centre of iKang Guobin Healthcare Group (Guangzhou, China) |
| Guangdong | CR-2 AF | Guangzhou Junan Medical Centre of iKang Healthcare Group (Guangzhou, China) |
| Guangdong | CR-2 AF | Guangzhou Nantian Medical Centre of iKang Healthcare Group (Guangzhou, China) |
| Guangdong | CR-2 AF | Guangzhou Tianhe Medical Centre of iKang Healthcare Group (Guangzhou, China) |
| Shenzhen | NT-2000 | Shenzhen Zhuoyue Medical Centre of iKang Healthcare Group (Shenzhen, China) |
| Shenzhen | CR-2 AF | Shenzhen Xinglin Medical Centre of iKang Guobin Healthcare Group (Shenzhen, China) |
| Hunan | CR-2 AF | Changsha shuangta Medical Centre of iKang Healthcare Group (Changsha, China) |
| Fujian | CR-2 AF | Fuzhou Gulou Medical Centre of iKang Guobin Healthcare Group (Fuzhou, China) |
| Chongqing | CR-2 AF | Chongqing Zhuoyue Medical Centre of iKang Healthcare Group (Chongqing, China) |
| Sichuan | CR-2 AF | Chengdu Anshengmei Medical Centre of iKang Guobin Healthcare Group (Chengdu, China) |
| Sichuan | CR-2 AF | Chengdu Luomashi Medical Centre of iKang Guobin Healthcare Group (Chengdu, China) |
| Sichuan | CR-2 AF | Chengdu Hongzhaobi Medical Centre of iKang Guobin Healthcare Group (Chengdu, China) |
| Sichuan | CR-2 AF | Mianyang Medical Centre of iKang Guobin Healthcare Group (Mianyang, China) |
| Ningxia | CR-2 AF | Guyuan Medical Centre of iKang Guobin Healthcare Group (Guyuan, China) |
| Ningxia | CR-2 AF | Yinchuan Medical Centre of iKang Guobin Healthcare Group (Yinchuan, China) |
| Guizhou | CR-2 AF | Liupanshui Medical Centre of iKang Guobin Healthcare Group (Liupanshui, China) |
| Guizhou | CR-2 AF | Kaili Medical Centre of iKang Guobin Healthcare Group (Kaili, China) |
| Guizhou | CR-2 AF | Bijie Medical Centre of iKang Guobin Healthcare Group (Bijie, China) |
| Guizhou | CR-2 AF | Guizhou Zhuoyue Medical Centre of iKang Guobin Healthcare Group (Guiyang, China) |

## Table S2. Standard population based on the seventh national census of China

| age | gender | Standard population | gender | Standard population |
| --- | --- | --- | --- | --- |
| 0 | male | 6312409 | Female | 5675648 |
| 1 | male | 7559981 | Female | 6823810 |
| 2 | male | 8020423 | Female | 7246355 |
| 3 | male | 9670005 | Female | 8748073 |
| 4 | male | 9406513 | Female | 8420671 |
| 5 | male | 8765848 | Female | 7781423 |
| 6 | male | 9881523 | Female | 8710283 |
| 7 | male | 9548981 | Female | 8414176 |
| 8 | male | 10317678 | Female | 9036074 |
| 9 | male | 9503428 | Female | 8284642 |
| 10 | male | 9285412 | Female | 8062153 |
| 11 | male | 9462024 | Female | 8233020 |
| 12 | male | 9279079 | Female | 8077840 |
| 13 | male | 8920655 | Female | 7756399 |
| 14 | male | 8659620 | Female | 7519792 |
| 15 | male | 8225995 | Female | 7097229 |
| 16 | male | 8195908 | Female | 7022248 |
| 17 | male | 7401041 | Female | 6329585 |
| 18 | male | 7560195 | Female | 6482984 |
| 19 | male | 7670204 | Female | 6698751 |
| 20 | male | 7742857 | Female | 6820490 |
| 21 | male | 7389412 | Female | 6546097 |
| 22 | male | 8078183 | Female | 7171626 |
| 23 | male | 8103460 | Female | 7222510 |
| 24 | male | 8362083 | Female | 7504957 |
| 25 | male | 9042474 | Female | 8103973 |
| 26 | male | 8845171 | Female | 7981817 |
| 27 | male | 9588428 | Female | 8710507 |
| 28 | male | 9975395 | Female | 9085250 |
| 29 | male | 10710802 | Female | 9803515 |
| 30 | male | 13463923 | Female | 12509159 |
| 31 | male | 13094627 | Female | 12356576 |
| 32 | male | 12335076 | Female | 11702635 |
| 33 | male | 13315974 | Female | 12630879 |
| 34 | male | 11662208 | Female | 11074133 |
| 35 | male | 10077433 | Female | 9508899 |
| 36 | male | 10034259 | Female | 9465682 |
| 37 | male | 9815714 | Female | 9248027 |
| 38 | male | 11217120 | Female | 10582164 |
| 39 | male | 9787511 | Female | 9276123 |
| 40 | male | 8997291 | Female | 8543426 |
| 41 | male | 9934985 | Female | 9404632 |
| 42 | male | 9492205 | Female | 9072006 |
| 43 | male | 9097039 | Female | 8661247 |
| 44 | male | 10111174 | Female | 9641325 |
| 45 | male | 10335133 | Female | 9924785 |
| 46 | male | 11305313 | Female | 10903672 |
| 47 | male | 11844299 | Female | 11389118 |
| 48 | male | 12147117 | Female | 11714893 |
| 49 | male | 12559824 | Female | 12100733 |
| 50 | male | 13149971 | Female | 12912065 |
| 51 | male | 12342864 | Female | 12101706 |
| 52 | male | 13149508 | Female | 12955572 |
| 53 | male | 10539231 | Female | 10420437 |
| 54 | male | 11923896 | Female | 11669046 |
| 55 | male | 11648724 | Female | 11457431 |
| 56 | male | 11301683 | Female | 11257663 |
| 57 | male | 13130492 | Female | 12913650 |
| 58 | male | 9562232 | Female | 9569864 |
| 59 | male | 5172895 | Female | 5386152 |
| 60 | male | 6442344 | Female | 6457541 |
| 61 | male | 6148108 | Female | 5987851 |
| 62 | male | 7832019 | Female | 7632258 |
| 63 | male | 8678055 | Female | 8553952 |
| 64 | male | 7770599 | Female | 7880211 |
| 65 | male | 8101750 | Female | 8193355 |
| 66 | male | 8003803 | Female | 8244652 |
| 67 | male | 7147712 | Female | 7472606 |
| 68 | male | 7115265 | Female | 7435214 |
| 69 | male | 5969393 | Female | 6321810 |
| 70 | male | 5667080 | Female | 5933693 |
| 71 | male | 5539299 | Female | 5689346 |
| 72 | male | 4640378 | Female | 4911856 |
| 73 | male | 4399581 | Female | 4646645 |
| 74 | male | 3916395 | Female | 4245763 |
| 75 | male | 3414021 | Female | 3765378 |
| 76 | male | 3265307 | Female | 3553654 |
| 77 | male | 2835339 | Female | 3184213 |
| 78 | male | 2657484 | Female | 3014552 |
| 79 | male | 2580282 | Female | 2968619 |
| 80 | male | 2271741 | Female | 2647347 |
| 81 | male | 1869035 | Female | 2270547 |
| 82 | male | 1914824 | Female | 2327654 |
| 83 | male | 1645840 | Female | 2065243 |
| 84 | male | 1455563 | Female | 1915084 |
| 85 | male | 1213783 | Female | 1695450 |
| 86 | male | 1034635 | Female | 1484029 |
| 87 | male | 935469 | Female | 1349612 |
| 88 | male | 687799 | Female | 1036640 |
| 89 | male | 554405 | Female | 834708 |
| 90 | male | 476404 | Female | 739712 |
| 91 | male | 315634 | Female | 527601 |
| 92 | male | 262078 | Female | 450885 |
| 93 | male | 183520 | Female | 328019 |
| 94 | male | 129958 | Female | 238938 |
| 95 | male | 98355 | Female | 191417 |
| 96 | male | 68067 | Female | 138151 |
| 97 | male | 46250 | Female | 97486 |
| 98 | male | 32835 | Female | 67781 |
| 99 | male | 25948 | Female | 53522 |
| 100 | male | 35129 | Female | 83737 |

# Table S3. Prevalence of retinal and optic nerve diseases in aged 20 to 24 participants.

|  | **Prevalence 2019** | | **Prevalence 2020** | | **Prevalence 2021** | | **Percentage change 2019-2021** | |
| --- | --- | --- | --- | --- | --- | --- | --- | --- |
|  | **n=9623** | | **n=20542** | | **n=10799** | |  |  |
|  | Crude | Adjusted | Crude | Adjusted | Crude | Adjusted | Crude | Adjusted |
| Diabetic retinopathy | 0.31 (0.06-0.91) | 0.24 (0.00-0.55) | 0.15 (0.03-0.43) | 0.10 (0.00-0.24) | 0.56 (0.20-1.21) | 1.13 (0.03-3.13) | 80.65% | 370.83% |
| Age-related macular degeneration | 0.52 (0.17-1.21) | 0.38 (0.10-0.65) | 0.73 (0.41-1.20) | 0.52 (0.16-0.85) | 0.46 (0.15-1.08) | 0.46 (0.07-1.02) | -11.54% | 21.05% |
| Referral possible glaucoma | 56.64 (52.10-61.44) | 57.80 (53.93-61.79) | 47.17 (44.31-50.16) | 49.18 (45.23-53.24) | 47.69 (43.75-51.88) | 55.10 (49.14-61.50) | -15.80% | -4.67% |
| Pathological myopia | 0.83 (0.36-1.64) | 0.80 (0.08-1.48) | 0.34 (0.14-0.70) | 0.27 (0.04-0.54) | 1.02 (0.51-1.82) | 0.63 (0.14-1.15) | 22.89% | -21.25% |
| Retinal vein occlusion | 0.21 (0.03-0.75) | 0.13 (0.00-0.31) | 0.00 (0.00-0.18) | 0.00 (0.00-0.00) | 0.00 (0.00-0.34) | 0.00 (0.00-0.00) | -100.00% | -100.00% |
| Macula hole | 0.00 (0.00-0.38) | 0.00 (0.00-0.00) | 0.05 (0.00-0.27) | 0.02 (0.00-0.05) | 0.09 (0.00-0.52) | 0.05 (0.00-0.15) | NA | NA |
| Epiretinal macular membrane | 1.04 (0.50-1.91) | 0.67 (0.19-1.12) | 0.49 (0.23-0.90) | 0.34 (0.04-0.63) | 0.93 (0.44-1.70) | 1.25 (0.36-2.56) | -10.58% | 86.57% |
| Hypertensive retinopathy | 0.00 (0.00-0.38) | 0.00 (0.00-0.00) | 0.00 (0.00-0.18) | 0.00 (0.00-0.00) | 0.00 (0.00-0.34) | 0.00 (0.00-0.00) | NA | NA |
| Myelinated fibers | 2.70 (1.77-3.96) | 2.74 (1.10-4.48) | 3.26 (2.53-4.14) | 3.12 (2.49-3.63) | 2.59 (1.72-3.75) | 2.99 (1.90-4.06) | -4.07% | 9.12% |
| Retinitis pigmentosa | 0.10 (0.00-0.58) | 0.19 (0.00-0.56) | 0.10 (0.01-0.35) | 0.06 (0.00-0.13) | 0.09 (0.00-0.52) | 0.12 (0.00-0.37) | -10.00% | -36.84% |

Crude, crude prevalence per 1000 participants; Adjusted, age and sex-adjusted prevalence per 1000 standard population. Data was expressed as the estimate (95% confidence interval)

# Table S4. Prevalence of retinal and optic nerve diseases in aged 25 to 29 participants.

|  | **Prevalence 2019** | | **Prevalence 2020** | | **Prevalence 2021** | | **Percentage change 2019-2021** | |
| --- | --- | --- | --- | --- | --- | --- | --- | --- |
|  | **n=88108** | | **n=117271** | | **n=50525** | |  |  |
|  | Crude | Adjusted | Crude | Adjusted | Crude | Adjusted | Crude | Adjusted |
| Diabetic retinopathy | 0.34 (0.23-0.49) | 0.33 (0.18-0.44) | 0.48 (0.36-0.62) | 0.49 (0.43-0.56) | 0.49 (0.32-0.73) | 0.56 (0.36-0.76) | 44.12% | 69.70% |
| Age-related macular degeneration | 1.26 (1.04-1.52) | 1.23 (0.88-1.55) | 0.89 (0.72-1.07) | 0.87 (0.66-1.07) | 0.95 (0.70-1.26) | 0.94 (0.72-1.17) | -24.60% | -23.58% |
| Referral possible glaucoma | 51.77 (50.31-53.25) | 53.03 (50.16-54.55) | 47.92 (46.71-49.16) | 48.84 (47.90-49.78) | 47.78 (45.93-49.67) | 49.92 (47.15-52.14) | -7.71% | -5.86% |
| Pathological myopia | 1.42 (1.18-1.69) | 1.38 (1.02-1.73) | 0.96 (0.79-1.16) | 0.97 (0.85-1.08) | 1.05 (0.79-1.37) | 1.05 (0.87-1.26) | -26.06% | -23.91% |
| Retinal vein occlusion | 0.05 (0.01-0.12) | 0.04 (0.01-0.08) | 0.02 (0.00-0.06) | 0.02 (0.00-0.05) | 0.04 (0.00-0.14) | 0.04 (0.00-0.08) | -20.00% | 0.00% |
| Macula hole | 0.09 (0.04-0.18) | 0.08 (0.04-0.12) | 0.09 (0.05-0.17) | 0.09 (0.04-0.16) | 0.16 (0.07-0.31) | 0.17 (0.07-0.26) | 77.78% | 112.50% |
| Epiretinal macular membrane | 1.08 (0.87-1.32) | 1.08 (0.84-1.26) | 0.94 (0.77-1.13) | 0.94 (0.76-1.09) | 0.93 (0.68-1.24) | 0.98 (0.74-1.19) | -13.89% | -9.26% |
| Hypertensive retinopathy | 0.02 (0.00-0.08) | 0.02 (0.00-0.07) | 0.03 (0.01-0.09) | 0.04 (0.01-0.07) | 0.08 (0.02-0.20) | 0.08 (0.02-0.14) | 300.00% | 300.00% |
| Myelinated fibers | 3.58 (3.19-3.99) | 3.61 (3.26-3.92) | 3.81 (3.47-4.18) | 3.82 (3.67-3.99) | 3.66 (3.15-4.23) | 3.67 (3.21-4.17) | 2.23% | 1.66% |
| Retinitis pigmentosa | 0.15 (0.08-0.25) | 0.15 (0.10-0.21) | 0.15 (0.09-0.24) | 0.15 (0.09-0.21) | 0.30 (0.17-0.49) | 0.31 (0.15-0.42) | 100.00% | 106.67% |

Crude, crude prevalence per 1000 participants; Adjusted, age and sex-adjusted prevalence per 1000 standard population. Data was expressed as the estimate (95% confidence interval)

# Table S5. Prevalence of retinal and optic nerve diseases in aged 30 to 34 participants.

|  | **Prevalence 2019** | | **Prevalence 2020** | | **Prevalence 2021** | | **Percentage change 2019-2021** | |
| --- | --- | --- | --- | --- | --- | --- | --- | --- |
|  | **n=127166** | | **n=159993** | | **n=65397** | |  |  |
|  | Crude | Adjusted | Crude | Adjusted | Crude | Adjusted | Crude | Adjusted |
| Diabetic retinopathy | 0.98 (0.82-1.17) | 0.93 (0.59-1.29) | 1.31 (1.14-1.50) | 1.27 (0.97-1.63) | 1.35 (1.08-1.66) | 1.32 (0.67-1.99) | 37.76% | 41.94% |
| Age-related macular degeneration | 2.30 (2.05-2.58) | 2.26 (1.94-2.61) | 1.74 (1.55-1.96) | 1.69 (1.42-2.01) | 1.67 (1.37-2.01) | 1.66 (1.48-1.90) | -27.39% | -26.55% |
| Referral possible glaucoma | 56.99 (55.72-58.28) | 56.24 (53.69-59.19) | 50.44 (49.37-51.52) | 49.91 (48.12-51.78) | 50.66 (48.99-52.37) | 51.03 (49.31-53.07) | -11.11% | -9.26% |
| Pathological myopia | 1.87 (1.64-2.12) | 1.88 (1.76-2.02) | 1.49 (1.31-1.70) | 1.48 (1.32-1.62) | 1.36 (1.09-1.67) | 1.35 (1.12-1.65) | -27.27% | -28.19% |
| Retinal vein occlusion | 0.03 (0.01-0.08) | 0.03 (0.01-0.06) | 0.04 (0.02-0.09) | 0.04 (0.02-0.06) | 0.15 (0.07-0.28) | 0.14 (0.05-0.28) | 400.00% | 366.67% |
| Macula hole | 0.12 (0.07-0.19) | 0.11 (0.10-0.13) | 0.14 (0.09-0.21) | 0.14 (0.08-0.19) | 0.12 (0.05-0.24) | 0.12 (0.08-0.18) | 0.00% | 9.09% |
| Epiretinal macular membrane | 1.67 (1.46-1.92) | 1.63 (1.33-1.94) | 1.58 (1.39-1.79) | 1.55 (1.21-1.87) | 1.45 (1.18-1.78) | 1.46 (1.26-1.63) | -13.17% | -10.43% |
| Hypertensive retinopathy | 0.10 (0.05-0.17) | 0.10 (0.08-0.11) | 0.14 (0.09-0.21) | 0.13 (0.09-0.17) | 0.12 (0.05-0.24) | 0.12 (0.05-0.21) | 20.00% | 20.00% |
| Myelinated fibers | 4.27 (3.92-4.64) | 4.24 (4.06-4.44) | 4.47 (4.15-4.81) | 4.46 (4.10-4.81) | 4.47 (3.97-5.01) | 4.46 (3.82-5.05) | 4.68% | 5.19% |
| Retinitis pigmentosa | 0.27 (0.19-0.37) | 0.27 (0.19-0.36) | 0.34 (0.25-0.44) | 0.33 (0.21-0.48) | 0.21 (0.12-0.36) | 0.21 (0.11-0.31) | -22.22% | -22.22% |

Crude, crude prevalence per 1000 participants; Adjusted, age and sex-adjusted prevalence per 1000 standard population. Data was expressed as the estimate (95% confidence interval)

# Table S6. Prevalence of retinal and optic nerve diseases in aged 35 to 39 participants.

|  | **Prevalence 2019** | | **Prevalence 2020** | | **Prevalence 2021** | | **Percentage change 2019-2021** | |
| --- | --- | --- | --- | --- | --- | --- | --- | --- |
|  | **n=105568** | | **n=134628** | | **n=52058** | |  |  |
|  | Crude | Adjusted | Crude | Adjusted | Crude | Adjusted | Crude | Adjusted |
| Diabetic retinopathy | 2.98 (2.66-3.33) | 2.89 (2.19-3.60) | 3.21 (2.91-3.53) | 3.13 (2.57-3.72) | 2.94 (2.49-3.44) | 2.95 (2.26-3.65) | -1.34% | 2.08% |
| Age-related macular degeneration | 4.02 (3.64-4.42) | 4.01 (3.37-4.62) | 2.87 (2.59-3.17) | 2.83 (2.48-3.32) | 2.94 (2.49-3.44) | 2.95 (2.20-3.75) | -26.87% | -26.43% |
| Referral possible glaucoma | 64.56 (63.08-66.05) | 63.78 (61.31-66.16) | 57.51 (56.28-58.77) | 56.99 (54.54-59.17) | 57.74 (55.75-59.78) | 57.82 (56.19-59.35) | -10.56% | -9.34% |
| Pathological myopia | 2.94 (2.62-3.28) | 3.02 (2.30-3.71) | 2.50 (2.24-2.78) | 2.51 (2.29-2.72) | 2.57 (2.16-3.05) | 2.57 (2.14-3.15) | -12.59% | -14.90% |
| Retinal vein occlusion | 0.09 (0.05-0.17) | 0.09 (0.02-0.17) | 0.17 (0.11-0.26) | 0.17 (0.12-0.23) | 0.27 (0.15-0.45) | 0.28 (0.09-0.46) | 200.00% | 211.11% |
| Macula hole | 0.14 (0.08-0.23) | 0.15 (0.06-0.24) | 0.16 (0.10-0.25) | 0.16 (0.15-0.18) | 0.27 (0.15-0.45) | 0.27 (0.19-0.36) | 92.86% | 80.00% |
| Epiretinal macular membrane | 2.47 (2.18-2.79) | 2.43 (2.20-2.67) | 1.97 (1.74-2.22) | 1.94 (1.60-2.27) | 2.29 (1.89-2.73) | 2.30 (1.92-2.63) | -7.29% | -5.35% |
| Hypertensive retinopathy | 0.14 (0.08-0.23) | 0.14 (0.08-0.19) | 0.31 (0.22-0.42) | 0.31 (0.22-0.40) | 0.52 (0.34-0.75) | 0.52 (0.34-0.82) | 271.43% | 271.43% |
| Myelinated fibers | 5.58 (5.14-6.05) | 5.55 (5.15-6.04) | 4.98 (4.61-5.37) | 4.95 (4.41-5.51) | 5.44 (4.82-6.11) | 5.44 (4.52-6.20) | -2.51% | -1.98% |
| Retinitis pigmentosa | 0.22 (0.14-0.33) | 0.21 (0.11-0.32) | 0.27 (0.19-0.37) | 0.27 (0.22-0.33) | 0.36 (0.22-0.57) | 0.37 (0.31-0.41) | 63.64% | 76.19% |

Crude, crude prevalence per 1000 participants; Adjusted, age and sex-adjusted prevalence per 1000 standard population. Data was expressed as the estimate (95% confidence interval)

# Table S7. Prevalence of retinal and optic nerve diseases in aged 40 to 44 participants.

|  | **Prevalence 2019** | | **Prevalence 2020** | | **Prevalence 2021** | | **Percentage change 2019-2021** | |
| --- | --- | --- | --- | --- | --- | --- | --- | --- |
|  | **n=71301** | | **n=93003** | | **n=33485** | |  |  |
|  | Crude | Adjusted | Crude | Adjusted | Crude | Adjusted | Crude | Adjusted |
| Diabetic retinopathy | 6.61 (6.02-7.23) | 6.48 (5.50-7.43) | 6.56 (6.05-7.10) | 6.40 (5.75-7.05) | 6.78 (5.93-7.72) | 6.86 (5.71-8.18) | 2.57% | 5.86% |
| Age-related macular degeneration | 6.47 (5.89-7.08) | 6.43 (5.83-6.99) | 5.58 (5.11-6.08) | 5.60 (4.70-6.47) | 5.35 (4.59-6.19) | 5.58 (4.05-7.11) | -17.31% | -13.22% |
| Referral possible glaucoma | 72.03 (70.15-73.95) | 70.82 (69.16-72.51) | 63.75 (62.19-65.34) | 63.14 (61.01-65.20) | 64.63 (62.02-67.31) | 64.14 (62.16-65.98) | -10.27% | -9.43% |
| Pathological myopia | 4.22 (3.76-4.73) | 4.30 (3.72-5.06) | 3.62 (3.25-4.03) | 3.69 (3.29-4.09) | 4.63 (3.93-5.42) | 4.80 (3.91-5.78) | 9.72% | 11.63% |
| Retinal vein occlusion | 0.35 (0.23-0.52) | 0.33 (0.29-0.38) | 0.54 (0.40-0.71) | 0.53 (0.40-0.66) | 0.63 (0.39-0.96) | 0.64 (0.51-0.81) | 80.00% | 93.94% |
| Macula hole | 0.18 (0.10-0.31) | 0.19 (0.11-0.26) | 0.22 (0.13-0.33) | 0.21 (0.16-0.26) | 0.24 (0.10-0.47) | 0.26 (0.17-0.34) | 33.33% | 36.84% |
| Epiretinal macular membrane | 4.17 (3.71-4.67) | 4.19 (3.68-4.57) | 3.57 (3.20-3.97) | 3.62 (3.04-4.19) | 3.40 (2.81-4.09) | 3.54 (2.63-4.45) | -18.47% | -15.51% |
| Hypertensive retinopathy | 0.35 (0.23-0.52) | 0.34 (0.17-0.50) | 0.42 (0.30-0.57) | 0.39 (0.30-0.48) | 0.72 (0.46-1.07) | 0.75 (0.52-0.98) | 105.71% | 120.59% |
| Myelinated fibers | 6.33 (5.76-6.93) | 6.35 (5.58-7.10) | 6.12 (5.63-6.64) | 6.11 (5.90-6.27) | 6.30 (5.48-7.21) | 6.26 (5.49-7.20) | -0.47% | -1.42% |
| Retinitis pigmentosa | 0.53 (0.38-0.73) | 0.53 (0.34-0.69) | 0.40 (0.28-0.55) | 0.40 (0.32-0.51) | 0.24 (0.10-0.47) | 0.22 (0.09-0.35) | -54.72% | -58.49% |

Crude, crude prevalence per 1000 participants; Adjusted, age and sex-adjusted prevalence per 1000 standard population. Data was expressed as the estimate (95% confidence interval)

# Table S8. Prevalence of retinal and optic nerve diseases in aged 45 to 49 participants.

|  | **Prevalence 2019** | | **Prevalence 2020** | | **Prevalence 2021** | | **Percentage change 2019-2021** | |
| --- | --- | --- | --- | --- | --- | --- | --- | --- |
|  | **n=58765** | | **n=73208** | | **n=27495** | |  |  |
|  | Crude | Adjusted | Crude | Adjusted | Crude | Adjusted | Crude | Adjusted |
| Diabetic retinopathy | 10.47 (9.66-11.32) | 10.07 (9.38-10.72) | 11.35 (10.60-12.15) | 10.96 (9.66-12.59) | 11.78 (10.54-13.13) | 11.88 (10.43-13.23) | 12.51% | 17.97% |
| Age-related macular degeneration | 11.28 (10.44-12.17) | 11.07 (9.63-12.37) | 10.89 (10.15-11.67) | 10.70 (9.07-12.40) | 10.18 (9.03-11.44) | 10.21 (8.58-11.74) | -9.75% | -7.77% |
| Referral possible glaucoma | 78.74 (76.57-80.94) | 77.72 (76.63-79.01) | 66.45 (64.66-68.28) | 65.69 (64.34-67.10) | 64.88 (62.00-67.86) | 65.11 (63.28-66.88) | -17.60% | -16.22% |
| Pathological myopia | 7.33 (6.66-8.06) | 7.41 (6.47-8.27) | 6.27 (5.71-6.87) | 6.32 (5.72-6.88) | 6.47 (5.56-7.49) | 6.50 (5.64-7.30) | -11.73% | -12.28% |
| Retinal vein occlusion | 0.56 (0.39-0.79) | 0.55 (0.34-0.79) | 1.02 (0.81-1.28) | 1.00 (0.71-1.25) | 1.27 (0.89-1.77) | 1.30 (0.79-1.80) | 126.79% | 136.36% |
| Macula hole | 0.20 (0.11-0.36) | 0.21 (0.11-0.33) | 0.59 (0.43-0.79) | 0.59 (0.43-0.75) | 0.36 (0.17-0.67) | 0.37 (0.21-0.53) | 80.00% | 76.19% |
| Epiretinal macular membrane | 6.16 (5.54-6.83) | 6.15 (4.86-7.55) | 6.78 (6.19-7.40) | 6.78 (5.04-8.53) | 6.11 (5.22-7.10) | 6.12 (4.69-7.40) | -0.81% | -0.49% |
| Hypertensive retinopathy | 0.36 (0.22-0.55) | 0.34 (0.21-0.51) | 0.51 (0.36-0.70) | 0.48 (0.31-0.65) | 1.35 (0.95-1.85) | 1.34 (0.94-1.78) | 275.00% | 294.12% |
| Myelinated fibers | 6.91 (6.26-7.61) | 6.84 (6.62-7.04) | 6.64 (6.06-7.25) | 6.61 (5.91-7.27) | 7.49 (6.51-8.58) | 7.46 (6.73-8.23) | 8.39% | 9.06% |
| Retinitis pigmentosa | 0.46 (0.30-0.67) | 0.45 (0.24-0.65) | 0.33 (0.21-0.49) | 0.34 (0.18-0.49) | 0.29 (0.13-0.57) | 0.29 (0.11-0.48) | -36.96% | -35.56% |

Crude, crude prevalence per 1000 participants; Adjusted, age and sex-adjusted prevalence per 1000 standard population. Data was expressed as the estimate (95% confidence interval)

# Table S9. Prevalence of retinal and optic nerve diseases in aged 50 to 54 participants.

|  | **Prevalence 2019** | | **Prevalence 2020** | | **Prevalence 2021** | | **Percentage change 2019-2021** | |
| --- | --- | --- | --- | --- | --- | --- | --- | --- |
|  | **n=60084** | | **n=79673** | | **n=38811** | |  |  |
|  | Crude | Adjusted | Crude | Adjusted | Crude | Adjusted | Crude | Adjusted |
| Diabetic retinopathy | 17.04 (16.02-18.11) | 16.77 (14.63-18.43) | 18.34 (17.42-19.29) | 18.10 (15.68-20.46) | 16.59 (15.35-17.91) | 17.18 (15.73-18.71) | -2.64% | 2.44% |
| Age-related macular degeneration | 16.49 (15.49-17.54) | 16.32 (15.52-17.19) | 17.41 (16.51-18.34) | 17.24 (15.73-18.90) | 16.62 (15.37-17.94) | 16.88 (14.87-18.59) | 0.79% | 3.43% |
| Referral possible glaucoma | 85.95 (83.72-88.22) | 85.40 (83.41-87.23) | 75.90 (74.07-77.76) | 75.44 (73.27-77.76) | 72.20 (69.64-74.82) | 72.82 (70.24-75.32) | -16.00% | -14.73% |
| Pathological myopia | 8.01 (7.31-8.75) | 8.00 (7.34-8.63) | 8.53 (7.91-9.20) | 8.50 (7.87-8.97) | 7.45 (6.62-8.35) | 7.27 (6.98-7.53) | -6.99% | -9.13% |
| Retinal vein occlusion | 1.12 (0.86-1.42) | 1.07 (0.79-1.44) | 1.79 (1.51-2.11) | 1.79 (1.55-2.05) | 2.06 (1.63-2.56) | 2.06 (1.65-2.53) | 83.93% | 92.52% |
| Macula hole | 0.33 (0.20-0.51) | 0.34 (0.22-0.46) | 0.60 (0.44-0.80) | 0.62 (0.51-0.77) | 0.59 (0.38-0.89) | 0.59 (0.36-0.82) | 78.79% | 73.53% |
| Epiretinal macular membrane | 11.60 (10.76-12.49) | 11.61 (9.11-14.10) | 12.15 (11.40-12.93) | 12.30 (10.05-14.95) | 11.70 (10.65-12.82) | 11.62 (9.55-13.33) | 0.86% | 0.09% |
| Hypertensive retinopathy | 0.82 (0.60-1.08) | 0.82 (0.61-1.02) | 0.92 (0.72-1.15) | 0.88 (0.73-1.09) | 1.47 (1.11-1.90) | 1.53 (1.34-1.74) | 79.27% | 86.59% |
| Myelinated fibers | 7.89 (7.20-8.63) | 7.84 (7.43-8.24) | 8.25 (7.63-8.90) | 8.30 (7.67-9.03) | 7.96 (7.10-8.90) | 8.19 (6.93-9.52) | 0.89% | 4.46% |
| Retinitis pigmentosa | 0.47 (0.31-0.67) | 0.48 (0.43-0.52) | 0.35 (0.23-0.51) | 0.35 (0.27-0.44) | 0.39 (0.22-0.64) | 0.40 (0.26-0.57) | -17.02% | -16.67% |

Crude, crude prevalence per 1000 participants; Adjusted, age and sex-adjusted prevalence per 1000 standard population. Data was expressed as the estimate (95% confidence interval)

# Table S10. Prevalence of retinal and optic nerve diseases in aged 55 to 59 participants.

|  | **Prevalence 2019** | | **Prevalence 2020** | | **Prevalence 2021** | | **Percentage change 2019-2021** | |
| --- | --- | --- | --- | --- | --- | --- | --- | --- |
|  | **n=58154** | | **n=82413** | | **n=41495** | |  |  |
|  | Crude | Adjusted | Crude | Adjusted | Crude | Adjusted | Crude | Adjusted |
| Diabetic retinopathy | 24.64 (23.40-25.93) | 23.06 (20.80-25.85) | 25.55 (24.49-26.65) | 24.96 (23.33-26.81) | 24.22 (22.76-25.74) | 24.60 (22.31-26.86) | -1.70% | 6.68% |
| Age-related macular degeneration | 25.57 (24.30-26.89) | 24.90 (23.20-26.61) | 24.56 (23.51-25.64) | 23.98 (22.40-26.00) | 22.82 (21.41-24.30) | 23.01 (21.22-25.16) | -10.75% | -7.59% |
| Referral possible glaucoma | 93.41 (91.05-95.80) | 91.21 (86.52-95.81) | 80.58 (78.73-82.46) | 78.94 (75.76-83.90) | 79.94 (77.35-82.59) | 79.99 (75.06-85.33) | -14.42% | -12.30% |
| Pathological myopia | 7.82 (7.12-8.57) | 7.86 (7.55-8.30) | 8.52 (7.90-9.17) | 8.53 (8.29-8.85) | 7.83 (7.01-8.73) | 7.63 (6.34-9.17) | 0.13% | -2.93% |
| Retinal vein occlusion | 1.51 (1.21-1.86) | 1.47 (1.18-1.76) | 2.63 (2.29-3.01) | 2.57 (2.12-2.98) | 3.52 (2.97-4.14) | 3.61 (3.31-3.94) | 133.11% | 145.58% |
| Macula hole | 0.76 (0.55-1.02) | 0.72 (0.52-1.00) | 1.31 (1.08-1.58) | 1.25 (1.03-1.60) | 1.33 (1.00-1.72) | 1.21 (0.94-1.44) | 75.00% | 68.06% |
| Epiretinal macular membrane | 25.29 (24.03-26.60) | 24.13 (20.57-29.14) | 26.10 (25.02-27.21) | 24.50 (20.16-30.07) | 27.16 (25.62-28.77) | 24.95 (20.16-31.15) | 7.39% | 3.40% |
| Hypertensive retinopathy | 1.10 (0.85-1.41) | 1.14 (0.87-1.33) | 1.37 (1.13-1.65) | 1.34 (1.24-1.46) | 1.64 (1.27-2.08) | 1.68 (1.30-2.02) | 49.09% | 47.37% |
| Myelinated fibers | 8.46 (7.73-9.24) | 8.42 (7.72-9.15) | 8.42 (7.81-9.07) | 8.49 (8.04-8.89) | 8.34 (7.49-9.26) | 8.32 (7.56-9.02) | -1.42% | -1.19% |
| Retinitis pigmentosa | 0.43 (0.28-0.63) | 0.43 (0.31-0.56) | 0.67 (0.50-0.87) | 0.65 (0.46-0.82) | 0.58 (0.37-0.86) | 0.58 (0.31-0.84) | 34.88% | 34.88% |

Crude, crude prevalence per 1000 participants; Adjusted, age and sex-adjusted prevalence per 1000 standard population. Data was expressed as the estimate (95% confidence interval)

# Table S11. Prevalence of retinal and optic nerve diseases in aged 60 to 64 participants.

|  | **Prevalence 2019** | | **Prevalence 2020** | | **Prevalence 2021** | | **Percentage change 2019-2021** | |
| --- | --- | --- | --- | --- | --- | --- | --- | --- |
|  | **n=32545** | | **n=48374** | | **n=23583** | |  |  |
|  | Crude | Adjusted | Crude | Adjusted | Crude | Adjusted | Crude | Adjusted |
| Diabetic retinopathy | 29.62 (27.81-31.52) | 29.37 (27.15-31.63) | 29.81 (28.31-31.36) | 30.14 (28.91-31.57) | 26.33 (24.33-28.46) | 26.66 (25.15-28.30) | -11.11% | -9.23% |
| Age-related macular degeneration | 35.64 (33.65-37.71) | 35.61 (33.12-37.91) | 32.15 (30.59-33.76) | 32.55 (29.67-35.47) | 31.00 (28.82-33.29) | 31.78 (29.64-33.82) | -13.02% | -10.76% |
| Referral possible glaucoma | 110.74 (107.35-114.20) | 110.77 (106.65-113.40) | 95.63 (93.02-98.29) | 96.41 (90.64-101.06) | 92.23 (88.56-95.99) | 92.81 (89.27-96.97) | -16.71% | -16.21% |
| Pathological myopia | 9.16 (8.15-10.25) | 9.19 (8.04-10.46) | 11.39 (10.46-12.38) | 11.36 (10.54-12.44) | 11.58 (10.25-13.02) | 11.29 (9.92-12.71) | 26.42% | 22.85% |
| Retinal vein occlusion | 2.89 (2.33-3.53) | 2.90 (2.54-3.23) | 3.60 (3.08-4.17) | 3.68 (2.92-4.32) | 4.88 (4.03-5.85) | 4.87 (4.51-5.29) | 68.86% | 67.93% |
| Macula hole | 1.60 (1.19-2.09) | 1.63 (0.94-2.41) | 2.23 (1.83-2.69) | 2.17 (1.41-2.94) | 2.42 (1.83-3.13) | 2.35 (1.56-3.03) | 51.25% | 44.17% |
| Epiretinal macular membrane | 54.23 (51.80-56.75) | 55.06 (45.47-62.77) | 57.57 (55.51-59.69) | 57.47 (48.77-64.81) | 59.75 (56.75-62.85) | 59.23 (47.66-68.57) | 10.18% | 7.57% |
| Hypertensive retinopathy | 1.35 (0.98-1.81) | 1.36 (1.13-1.51) | 1.03 (0.77-1.36) | 1.04 (1.01-1.07) | 1.48 (1.03-2.06) | 1.48 (1.34-1.61) | 9.63% | 8.82% |
| Myelinated fibers | 8.88 (7.89-9.96) | 8.97 (7.95-10.10) | 9.41 (8.56-10.31) | 9.51 (8.66-10.37) | 9.63 (8.42-10.96) | 9.83 (8.84-10.76) | 8.45% | 9.59% |
| Retinitis pigmentosa | 0.61 (0.38-0.95) | 0.58 (0.30-0.87) | 0.62 (0.42-0.89) | 0.64 (0.45-0.82) | 1.19 (0.79-1.72) | 1.23 (0.91-1.63) | 95.08% | 112.07% |

Crude, crude prevalence per 1000 participants; Adjusted, age and sex-adjusted prevalence per 1000 standard population. Data was expressed as the estimate (95% confidence interval)

# Table S12. Prevalence of retinal and optic nerve diseases in aged 65 to 69 participants.

|  | **Prevalence 2019** | | **Prevalence 2020** | | **Prevalence 2021** | | **Percentage change 2019-2021** | |
| --- | --- | --- | --- | --- | --- | --- | --- | --- |
|  | **n=25845** | | **n=37411** | | **n=16786** | |  |  |
|  | Crude | Adjusted | Crude | Adjusted | Crude | Adjusted | Crude | Adjusted |
| Diabetic retinopathy | 27.55 (25.59-29.62) | 27.46 (25.65-29.20) | 26.86 (25.25-28.55) | 26.82 (25.46-28.32) | 25.14 (22.82-27.62) | 25.17 (24.04-26.27) | -8.75% | -8.34% |
| Age-related macular degeneration | 40.24 (37.88-42.71) | 40.21 (39.47-40.99) | 37.93 (36.02-39.92) | 38.03 (36.46-39.65) | 36.46 (33.67-39.41) | 36.82 (35.28-38.23) | -9.39% | -8.43% |
| Referral possible glaucoma | 113.95 (110.10-117.89) | 114.14 (110.55-118.27) | 102.35 (99.30-105.47) | 102.53 (99.72-104.86) | 97.52 (93.07-102.11) | 98.23 (94.23-102.64) | -14.42% | -13.94% |
| Pathological myopia | 11.92 (10.63-13.32) | 12.05 (10.53-13.72) | 14.41 (13.22-15.67) | 14.54 (13.87-15.57) | 15.07 (13.28-17.03) | 15.14 (13.16-16.80) | 26.43% | 25.64% |
| Retinal vein occlusion | 2.86 (2.25-3.59) | 2.87 (2.02-3.59) | 4.25 (3.62-4.96) | 4.27 (3.57-4.83) | 5.84 (4.74-7.11) | 5.96 (4.68-7.42) | 104.20% | 107.67% |
| Macula hole | 2.21 (1.67-2.86) | 2.25 (1.79-2.75) | 4.06 (3.44-4.76) | 4.06 (3.56-4.68) | 4.77 (3.78-5.93) | 4.75 (3.93-5.69) | 115.84% | 111.11% |
| Epiretinal macular membrane | 96.00 (92.43-99.65) | 97.20 (87.76-108.06) | 91.95 (89.04-94.92) | 93.38 (84.53-104.20) | 94.13 (89.75-98.64) | 94.88 (88.85-101.81) | -1.95% | -2.39% |
| Hypertensive retinopathy | 1.35 (0.94-1.88) | 1.37 (1.17-1.59) | 1.36 (1.02-1.79) | 1.32 (0.82-1.86) | 1.55 (1.01-2.27) | 1.60 (1.32-1.90) | 14.81% | 16.79% |
| Myelinated fibers | 7.70 (6.67-8.84) | 7.69 (6.52-8.63) | 7.81 (6.94-8.75) | 7.77 (7.21-8.44) | 7.39 (6.15-8.80) | 7.24 (5.64-8.96) | -4.03% | -5.85% |
| Retinitis pigmentosa | 0.62 (0.35-1.01) | 0.60 (0.35-0.94) | 0.45 (0.26-0.73) | 0.45 (0.36-0.54) | 0.42 (0.17-0.86) | 0.40 (0.18-0.64) | -32.26% | -33.33% |

Crude, crude prevalence per 1000 participants; Adjusted, age and sex-adjusted prevalence per 1000 standard population. Data was expressed as the estimate (95% confidence interval)

# Table S13. Prevalence of retinal and optic nerve diseases in aged 70 to 74 participants.

|  | **Prevalence 2019** | | **Prevalence 2020** | | **Prevalence 2021** | | **Percentage change 2019-2021** | |
| --- | --- | --- | --- | --- | --- | --- | --- | --- |
|  | **n=11054** | | **n=15472** | | **n=6093** | |  |  |
|  | Crude | Adjusted | Crude | Adjusted | Crude | Adjusted | Crude | Adjusted |
| Diabetic retinopathy | 30.85 (27.70-34.24) | 30.27 (26.17-36.35) | 30.51 (27.85-33.34) | 30.21 (28.17-32.44) | 28.39 (24.37-32.88) | 28.21 (25.10-31.07) | -7.97% | -6.81% |
| Age-related macular degeneration | 44.60 (40.83-48.61) | 43.64 (40.91-47.63) | 43.56 (40.40-46.90) | 42.99 (40.78-44.60) | 38.73 (34.03-43.89) | 38.72 (36.17-43.45) | -13.16% | -11.27% |
| Referral possible glaucoma | 128.91 (122.72-135.30) | 127.87 (123.04-133.52) | 105.93 (101.13-110.89) | 105.72 (98.67-113.13) | 104.87 (97.29-112.84) | 105.56 (99.64-113.23) | -18.65% | -17.45% |
| Pathological myopia | 20.08 (17.55-22.87) | 20.68 (18.98-22.06) | 24.75 (22.36-27.33) | 25.67 (23.46-28.46) | 22.65 (19.06-26.70) | 24.16 (17.51-32.46) | 12.80% | 16.83% |
| Retinal vein occlusion | 4.16 (3.05-5.55) | 4.19 (2.66-5.73) | 4.52 (3.53-5.71) | 4.63 (4.08-5.20) | 6.73 (4.83-9.12) | 6.56 (3.28-9.03) | 61.78% | 56.56% |
| Macula hole | 3.89 (2.82-5.24) | 3.97 (3.25-4.93) | 3.88 (2.96-4.99) | 4.13 (2.94-5.60) | 5.91 (4.14-8.17) | 6.12 (4.13-7.87) | 51.93% | 54.16% |
| Epiretinal macular membrane | 121.40 (115.37-127.64) | 122.15 (116.26-128.19) | 110.91 (106.00-115.96) | 110.73 (108.38-111.91) | 110.13 (102.37-118.26) | 109.84 (98.44-120.33) | -9.28% | -10.08% |
| Hypertensive retinopathy | 1.27 (0.69-2.12) | 1.28 (0.59-2.20) | 1.23 (0.74-1.92) | 1.27 (1.00-1.59) | 0.82 (0.27-1.91) | 0.85 (0.16-1.58) | -35.43% | -33.59% |
| Myelinated fibers | 7.24 (5.74-9.00) | 7.15 (5.31-9.42) | 8.14 (6.79-9.69) | 8.27 (7.73-8.90) | 7.22 (5.25-9.68) | 6.81 (3.17-10.32) | -0.28% | -4.76% |
| Retinitis pigmentosa | 0.36 (0.10-0.93) | 0.33 (0.09-0.54) | 0.45 (0.18-0.93) | 0.45 (0.07-0.88) | 0.66 (0.18-1.68) | 0.57 (0.16-0.91) | 83.33% | 72.73% |

Crude, crude prevalence per 1000 participants; Adjusted, age and sex-adjusted prevalence per 1000 standard population. Data was expressed as the estimate (95% confidence interval)

# Table S14. Prevalence of retinal and optic nerve diseases in aged 75 to 79 participants.

|  | **Prevalence 2019** | | **Prevalence 2020** | | **Prevalence 2021** | | **Percentage change 2019-2021** | |
| --- | --- | --- | --- | --- | --- | --- | --- | --- |
|  | **n=5606** | | **n=6906** | | **n=2442** | |  |  |
|  | Crude | Adjusted | Crude | Adjusted | Crude | Adjusted | Crude | Adjusted |
| Diabetic retinopathy | 31.22 (26.82-36.11) | 30.93 (27.24-33.38) | 32.29 (28.25-36.73) | 32.77 (30.65-35.86) | 30.71 (24.23-38.35) | 30.03 (22.25-38.65) | -1.63% | -2.91% |
| Age-related macular degeneration | 52.62 (46.92-58.79) | 51.25 (48.31-53.20) | 45.47 (40.67-50.65) | 45.24 (41.82-50.09) | 46.27 (38.29-55.37) | 46.15 (36.08-56.84) | -12.07% | -9.95% |
| Referral possible glaucoma | 127.90 (119.26-136.92) | 127.82 (117.86-136.79) | 118.74 (111.20-126.60) | 118.29 (109.90-124.63) | 119.57 (106.96-133.11) | 118.80 (111.20-127.48) | -6.51% | -7.06% |
| Pathological myopia | 24.26 (20.39-28.63) | 24.69 (21.62-27.57) | 32.15 (28.11-36.58) | 33.13 (30.17-36.58) | 31.12 (24.60-38.80) | 31.66 (27.34-34.70) | 28.28% | 28.23% |
| Retinal vein occlusion | 3.39 (2.04-5.29) | 3.35 (2.10-4.36) | 5.21 (3.65-7.21) | 5.17 (4.39-5.85) | 5.32 (2.84-9.09) | 5.41 (1.97-8.74) | 56.93% | 61.49% |
| Macula hole | 1.78 (0.86-3.28) | 1.77 (0.87-2.62) | 5.07 (3.53-7.04) | 5.17 (3.85-6.32) | 5.32 (2.84-9.09) | 5.33 (1.91-9.12) | 198.88% | 201.13% |
| Epiretinal macular membrane | 105.07 (97.16-113.39) | 103.31 (88.54-113.06) | 103.24 (96.16-110.66) | 101.56 (92.34-108.22) | 87.63 (76.71-99.55) | 85.40 (64.18-103.29) | -16.60% | -17.34% |
| Hypertensive retinopathy | 2.14 (1.11-3.74) | 1.92 (1.07-2.65) | 2.03 (1.11-3.40) | 2.03 (1.42-2.58) | 0.82 (0.10-2.96) | 0.78 (0.00-1.60) | -61.68% | -59.38% |
| Myelinated fibers | 6.24 (4.35-8.67) | 6.13 (5.06-7.39) | 7.38 (5.50-9.70) | 7.21 (6.12-8.28) | 5.73 (3.14-9.60) | 5.96 (4.94-7.27) | -8.17% | -2.77% |
| Retinitis pigmentosa | 0.36 (0.04-1.29) | 0.28 (0.00-0.59) | 0.58 (0.16-1.48) | 0.54 (0.14-0.92) | 0.82 (0.10-2.96) | 0.98 (0.00-2.24) | 127.78% | 250.00% |

Crude, crude prevalence per 1000 participants; Adjusted, age and sex-adjusted prevalence per 1000 standard population. Data was expressed as the estimate (95% confidence interval)

# Table S15. Prevalence of retinal and optic nerve diseases in aged 80 or above participants.

|  | **Prevalence 2019** | | **Prevalence 2020** | | **Prevalence 2021** | | **Percentage change 2019-2021** | |
| --- | --- | --- | --- | --- | --- | --- | --- | --- |
|  | **n=9623** | | **n=20542** | | **n=10799** | |  |  |
|  | Crude | Adjusted | Crude | Adjusted | Crude | Adjusted | Crude | Adjusted |
| Diabetic retinopathy | 23.83 (19.73-28.52) | 26.02 (22.26-31.01) | 30.09 (25.72-34.98) | 31.51 (26.12-38.04) | 29.87 (22.39-38.98) | 29.67 (18.35-42.51) | 25.35% | 14.03% |
| Age-related macular degeneration | 47.46 (41.66-53.82) | 47.66 (40.35-55.67) | 49.91 (44.28-56.03) | 51.83 (44.65-60.23) | 52.84 (42.81-64.42) | 66.09 (49.84-90.57) | 11.34% | 38.67% |
| Referral possible glaucoma | 139.92 (130.29-149.99) | 138.43 (126.51-151.30) | 114.31 (105.98-123.06) | 111.10 (101.00-122.85) | 118.32 (103.52-134.43) | 108.85 (86.97-125.68) | -15.44% | -21.37% |
| Pathological myopia | 26.30 (21.99-31.19) | 34.37 (26.66-46.36) | 33.21 (28.61-38.32) | 45.29 (32.17-68.53) | 44.80 (35.57-55.60) | 50.09 (37.98-66.59) | 70.34% | 45.74% |
| Retinal vein occlusion | 3.29 (1.88-5.33) | 3.82 (1.83-5.99) | 5.32 (3.57-7.63) | 5.27 (2.64-7.80) | 4.02 (1.62-8.27) | 6.31 (2.11-13.82) | 22.19% | 65.18% |
| Macula hole | 0.62 (0.13-1.80) | 0.94 (0.00-2.27) | 2.94 (1.68-4.76) | 2.90 (1.77-3.84) | 2.87 (0.93-6.69) | 2.81 (0.82-5.00) | 362.90% | 198.94% |
| Epiretinal macular membrane | 79.52 (72.07-87.47) | 74.05 (65.14-80.29) | 90.46 (82.97-98.39) | 86.87 (75.28-96.38) | 84.43 (71.80-98.49) | 75.15 (60.11-85.58) | 6.17% | 1.49% |
| Hypertensive retinopathy | 1.03 (0.33-2.40) | 1.16 (0.20-2.33) | 1.10 (0.40-2.39) | 0.96 (0.43-1.47) | 1.15 (0.14-4.14) | 0.92 (0.00-2.03) | 11.65% | -20.69% |
| Myelinated fibers | 6.78 (4.67-9.51) | 5.82 (2.32-9.41) | 7.52 (5.40-10.19) | 6.62 (4.77-8.70) | 4.60 (1.99-9.03) | 4.67 (1.47-8.66) | -32.15% | -19.76% |
| Retinitis pigmentosa | 0.41 (0.05-1.48) | 0.29 (0.00-0.66) | 1.10 (0.40-2.39) | 0.77 (0.00-1.53) | 0.00 (0.00-2.12) | 0.00 (0.00-0.00) | NA | NA |

Crude, crude prevalence per 1000 participants; Adjusted, age and sex-adjusted prevalence per 1000 standard population. Data was expressed as the estimate (95% confidence interval)

# Table S16. Prevalence of retinal and optic nerve diseases in working population (aged 15-64).

|  | **Prevalence 2019** | | **Prevalence 2020** | | **Prevalence 2021** | | **Percentage change 2019-2021** | |
| --- | --- | --- | --- | --- | --- | --- | --- | --- |
|  | **n=611618** | | **n=809608** | | **n=343911** | |  |  |
|  | Crude | Adjusted | Crude | Adjusted | Crude | Adjusted | Crude | Adjusted |
| Diabetic retinopathy | 8.14 (7.92-8.37) | 9.04 (6.57-11.56) | 8.83 (8.63-9.04) | 9.63 (6.79-12.52) | 8.99 (8.68-9.31) | 9.44 (6.84-12.24) | 10.44% | 4.42% |
| Age-related macular degeneration | 9.15 (8.91-9.39) | 10.23 (7.44-13.10) | 8.73 (8.53-8.93) | 9.59 (6.93-12.54) | 9.01 (8.70-9.33) | 9.57 (7.02-12.35) | -1.53% | -6.45% |
| Referral possible glaucoma | 70.54 (69.90-71.18) | 69.62 (63.70-75.51) | 62.43 (61.90-62.96) | 63.75 (59.37-68.01) | 62.52 (61.71-63.33) | 62.33 (57.97-66.80) | -11.37% | -10.47% |
| Pathological myopia | 4.33 (4.16-4.50) | 4.56 (3.62-5.47) | 4.23 (4.09-4.37) | 4.49 (3.42-5.53) | 4.38 (4.16-4.61) | 4.39 (3.45-5.36) | 1.15% | -3.73% |
| Retinal vein occlusion | 0.53 (0.48-0.60) | 0.63 (0.42-0.87) | 0.85 (0.79-0.92) | 0.97 (0.65-1.29) | 1.23 (1.12-1.35) | 1.27 (0.88-1.68) | 132.08% | 101.59% |
| Macula hole | 0.29 (0.25-0.34) | 0.32 (0.21-0.47) | 0.47 (0.43-0.52) | 0.51 (0.35-0.69) | 0.54 (0.46-0.62) | 0.51 (0.34-0.71) | 86.21% | 59.38% |
| Epiretinal macular membrane | 8.46 (8.23-8.69) | 10.02 (6.36-14.19) | 9.10 (8.90-9.31) | 10.28 (6.55-14.50) | 10.30 (9.97-10.65) | 10.26 (6.47-15.05) | 21.75% | 2.40% |
| Hypertensive retinopathy | 0.38 (0.33-0.43) | 0.43 (0.30-0.57) | 0.47 (0.42-0.52) | 0.48 (0.35-0.61) | 0.76 (0.67-0.85) | 0.79 (0.59-0.99) | 100.00% | 83.72% |
| Myelinated fibers | 5.86 (5.67-6.06) | 5.64 (4.96-6.33) | 5.88 (5.72-6.05) | 5.76 (5.08-6.43) | 6.07 (5.81-6.34) | 6.15 (5.33-7.02) | 3.58% | 9.04% |
| Retinitis pigmentosa | 0.34 (0.30-0.39) | 0.34 (0.27-0.41) | 0.35 (0.31-0.39) | 0.33 (0.26-0.39) | 0.38 (0.32-0.46) | 0.36 (0.27-0.47) | 11.76% | 5.88% |

Crude, crude prevalence per 1000 participants; Adjusted, age and sex-adjusted prevalence per 1000 standard population. Data was expressed as the estimate (95% confidence interval)

# Table S17. Prevalence of retinal and optic nerve diseases in female participants.

|  | **Prevalence 2019** | | **Prevalence 2020** | | **Prevalence 2021** | | **Percentage change 2019-2021** | |
| --- | --- | --- | --- | --- | --- | --- | --- | --- |
|  | **n=313146** | | **n=419177** | | **n=191945** | |  |  |
|  | Crude | Adjusted | Crude | Adjusted | Crude | Adjusted | Crude | Adjusted |
| Diabetic retinopathy | 5.42 (5.17-5.69) | 7.71 (5.90-9.84) | 6.25 (6.01-6.49) | 8.29 (6.26-10.23) | 6.81 (6.45-7.19) | 8.33 (6.21-10.59) | 25.65% | 8.04% |
| Age-related macular degeneration | 8.33 (8.01-8.65) | 11.39 (8.90-14.36) | 8.62 (8.35-8.91) | 11.42 (9.00-14.15) | 8.70 (8.29-9.13) | 12.47 (9.78-15.59) | 4.44% | 9.48% |
| Referral possible glaucoma | 57.48 (56.67-58.31) | 65.84 (55.31-80.71) | 52.90 (52.23-53.58) | 55.87 (49.78-62.33) | 53.05 (52.05-54.06) | 51.90 (45.88-57.91) | -7.71% | -21.17% |
| Pathological myopia | 6.08 (5.81-6.36) | 8.11 (6.57-9.74) | 6.27 (6.03-6.51) | 8.99 (6.97-11.31) | 6.41 (6.06-6.78) | 9.29 (7.31-11.61) | 5.43% | 14.55% |
| Retinal vein occlusion | 0.57 (0.49-0.67) | 0.90 (0.64-1.22) | 0.89 (0.81-0.99) | 1.25 (0.90-1.64) | 1.25 (1.10-1.42) | 1.75 (1.28-2.40) | 119.30% | 94.44% |
| Macula hole | 0.61 (0.53-0.71) | 0.82 (0.55-1.14) | 1.02 (0.92-1.12) | 1.32 (0.94-1.76) | 1.16 (1.01-1.32) | 1.58 (1.07-2.15) | 90.16% | 92.68% |
| Epiretinal macular membrane | 16.37 (15.93-16.82) | 25.10 (17.76-34.27) | 17.62 (17.22-18.02) | 24.65 (17.56-32.48) | 18.06 (17.47-18.66) | 23.92 (16.75-31.69) | 10.32% | -4.70% |
| Hypertensive retinopathy | 0.27 (0.22-0.34) | 0.38 (0.26-0.51) | 0.32 (0.27-0.38) | 0.41 (0.29-0.54) | 0.48 (0.39-0.59) | 0.51 (0.35-0.68) | 77.78% | 34.21% |
| Myelinated fibers | 5.14 (4.89-5.39) | 4.60 (4.02-5.21) | 5.42 (5.20-5.65) | 4.88 (4.28-5.50) | 5.60 (5.27-5.94) | 5.09 (4.46-5.77) | 8.95% | 10.65% |
| Retinitis pigmentosa | 0.39 (0.32-0.47) | 0.35 (0.26-0.45) | 0.38 (0.32-0.45) | 0.35 (0.26-0.43) | 0.42 (0.34-0.52) | 0.38 (0.26-0.51) | 7.69% | 8.57% |

Crude, crude prevalence per 1000 participants; Adjusted, age and sex-adjusted prevalence per 1000 standard population. Data was expressed as the estimate (95% confidence interval)

# Table S18. Prevalence of retinal and optic nerve diseases in male participants.

|  | **Prevalence 2019** | | **Prevalence 2020** | | **Prevalence 2021** | | **Percentage change 2019-2021** | |
| --- | --- | --- | --- | --- | --- | --- | --- | --- |
|  | **n=345880** | | **n=455725** | | **n=179054** | |  |  |
|  | Crude | Adjusted | Crude | Adjusted | Crude | Adjusted | Crude | Adjusted |
| Diabetic retinopathy | 13.37 (12.99-13.76) | 14.11 (10.98-17.43) | 14.04 (13.70-14.38) | 14.22 (11.04-17.61) | 14.00 (13.46-14.56) | 14.19 (11.37-17.21) | 4.71% | 0.57% |
| Age-related macular degeneration | 14.59 (14.20-15.00) | 16.91 (13.29-21.10) | 13.45 (13.12-13.79) | 15.03 (11.69-18.64) | 13.86 (13.32-14.41) | 15.33 (11.70-19.09) | -5.00% | -9.34% |
| Referral possible glaucoma | 89.37 (88.42-90.33) | 86.12 (75.20-98.11) | 77.42 (76.65-78.20) | 76.93 (65.21-92.50) | 78.71 (77.46-79.97) | 74.15 (66.52-81.03) | -11.93% | -13.90% |
| Pathological myopia | 4.44 (4.22-4.67) | 4.90 (4.00-5.88) | 4.66 (4.46-4.86) | 5.32 (4.24-6.52) | 4.59 (4.28-4.91) | 5.49 (4.43-6.78) | 3.38% | 12.04% |
| Retinal vein occlusion | 0.87 (0.78-0.98) | 1.07 (0.78-1.40) | 1.34 (1.23-1.45) | 1.53 (1.15-1.92) | 1.91 (1.71-2.12) | 2.01 (1.47-2.58) | 119.54% | 87.85% |
| Macula hole | 0.29 (0.24-0.35) | 0.38 (0.25-0.54) | 0.48 (0.42-0.55) | 0.62 (0.45-0.84) | 0.53 (0.43-0.65) | 0.67 (0.46-0.95) | 82.76% | 76.32% |
| Epiretinal macular membrane | 14.01 (13.62-14.40) | 19.77 (14.33-26.39) | 13.93 (13.59-14.27) | 19.02 (13.79-25.17) | 15.02 (14.46-15.59) | 19.86 (14.06-25.95) | 7.21% | 0.46% |
| Hypertensive retinopathy | 0.62 (0.54-0.70) | 0.67 (0.51-0.85) | 0.74 (0.66-0.82) | 0.68 (0.52-0.85) | 1.13 (0.98-1.29) | 1.02 (0.76-1.28) | 82.26% | 52.24% |
| Myelinated fibers | 6.72 (6.45-7.00) | 7.75 (5.27-11.93) | 6.58 (6.35-6.82) | 5.76 (4.95-6.61) | 6.72 (6.35-7.11) | 6.18 (5.13-7.41) | 0.00% | -20.26% |
| Retinitis pigmentosa | 0.32 (0.26-0.39) | 0.28 (0.22-0.36) | 0.35 (0.29-0.41) | 0.29 (0.23-0.35) | 0.36 (0.28-0.46) | 0.31 (0.22-0.41) | 12.50% | 10.71% |

Crude, crude prevalence per 1000 participants; Adjusted, age and sex-adjusted prevalence per 1000 standard population. Data was expressed as the estimate (95% confidence interval)

# Table S19. Prevalence of retinal and optic nerve diseases in Anhui province.

|  | **Prevalence 2019** | | **Prevalence 2020** | | **Prevalence 2021** | | **Percentage change 2019-2021** | |
| --- | --- | --- | --- | --- | --- | --- | --- | --- |
|  | **n=13413** | | **n=8679** | | **n=4293** | |  |  |
|  | Crude | Adjusted | Crude | Adjusted | Crude | Adjusted | Crude | Adjusted |
| Diabetic retinopathy | 8.42 (6.95-10.12) | 9.32 (6.27-12.61) | 8.99 (7.11-11.20) | 10.71 (7.58-14.33) | 13.28 (10.07-17.17) | 12.22 (8.00-16.32) | 57.72% | 31.12% |
| Age-related macular degeneration | 13.94 (12.03-16.07) | 17.76 (13.36-22.79) | 14.40 (12.00-17.14) | 17.17 (12.29-22.48) | 12.81 (9.67-16.64) | 12.23 (7.97-16.82) | -8.11% | -31.14% |
| Referral possible glaucoma | 70.53 (66.25-74.99) | 80.04 (70.91-91.00) | 61.07 (56.12-66.31) | 64.17 (56.26-73.04) | 60.80 (53.83-68.37) | 59.42 (50.14-69.38) | -13.80% | -25.76% |
| Pathological myopia | 6.34 (5.06-7.83) | 9.24 (5.94-13.79) | 5.30 (3.88-7.06) | 8.02 (4.74-12.08) | 7.45 (5.10-10.51) | 7.59 (4.57-11.03) | 17.51% | -17.86% |
| Retinal vein occlusion | 0.89 (0.46-1.56) | 1.04 (0.44-1.74) | 1.15 (0.55-2.12) | 1.75 (0.69-3.05) | 1.63 (0.66-3.36) | 2.13 (0.44-4.40) | 83.15% | 104.81% |
| Macula hole | 0.67 (0.31-1.27) | 0.87 (0.26-1.66) | 0.46 (0.13-1.18) | 0.88 (0.07-2.12) | 0.93 (0.25-2.38) | 0.90 (0.05-2.15) | 38.81% | 3.45% |
| Epiretinal macular membrane | 12.30 (10.51-14.31) | 19.62 (13.34-27.49) | 13.60 (11.27-16.26) | 20.49 (13.66-28.38) | 15.61 (12.11-19.78) | 18.46 (10.77-27.93) | 26.91% | -5.91% |
| Hypertensive retinopathy | 0.22 (0.05-0.65) | 0.21 (0.00-0.55) | 0.58 (0.19-1.34) | 0.58 (0.13-1.17) | 0.47 (0.06-1.68) | 0.44 (0.00-1.24) | 113.64% | 109.52% |
| Myelinated fibers | 6.64 (5.33-8.16) | 6.63 (4.94-8.54) | 7.14 (5.48-9.15) | 6.75 (4.83-8.74) | 6.76 (4.53-9.69) | 5.87 (3.54-8.31) | 1.81% | -11.46% |
| Retinitis pigmentosa | 0.37 (0.12-0.87) | 0.30 (0.06-0.58) | 0.46 (0.13-1.18) | 0.39 (0.00-0.81) | 0.23 (0.01-1.30) | 0.16 (0.00-0.58) | -37.84% | -46.67% |

Crude, crude prevalence per 1000 participants; Adjusted, age and sex-adjusted prevalence per 1000 standard population. Data was expressed as the estimate (95% confidence interval)

# Table S20. Prevalence of retinal and optic nerve diseases in Beijing.

|  | **Prevalence 2019** | | **Prevalence 2020** | | **Prevalence 2021** | | **Percentage change 2019-2021** | |
| --- | --- | --- | --- | --- | --- | --- | --- | --- |
|  | **n=120514** | | **n=191169** | | **n=93068** | |  |  |
|  | Crude | Adjusted | Crude | Adjusted | Crude | Adjusted | Crude | Adjusted |
| Diabetic retinopathy | 11.24 (10.65-11.85) | 14.06 (11.25-17.31) | 11.99 (11.51-12.49) | 13.32 (10.67-16.26) | 12.40 (11.70-13.13) | 15.24 (12.18-18.46) | 10.32% | 8.39% |
| Age-related macular degeneration | 11.49 (10.90-12.11) | 15.33 (12.01-18.93) | 9.59 (9.16-10.04) | 11.68 (9.22-14.41) | 10.65 (10.00-11.33) | 15.55 (12.20-19.51) | -7.31% | 1.44% |
| Referral possible glaucoma | 72.94 (71.48-74.42) | 82.77 (70.86-98.23) | 69.63 (68.50-70.78) | 75.86 (63.79-94.64) | 66.80 (65.20-68.42) | 66.68 (61.18-71.93) | -8.42% | -19.44% |
| Pathological myopia | 3.70 (3.37-4.06) | 4.75 (3.84-5.88) | 3.71 (3.45-4.00) | 4.69 (3.79-5.78) | 4.35 (3.94-4.80) | 6.42 (5.06-7.91) | 17.57% | 35.16% |
| Retinal vein occlusion | 0.81 (0.66-0.99) | 1.14 (0.80-1.52) | 1.14 (0.99-1.30) | 1.40 (1.04-1.78) | 1.55 (1.31-1.82) | 2.06 (1.44-2.70) | 91.36% | 80.70% |
| Macula hole | 0.46 (0.34-0.59) | 0.65 (0.42-0.93) | 0.70 (0.59-0.83) | 0.89 (0.61-1.19) | 0.80 (0.62-1.00) | 1.22 (0.78-1.75) | 73.91% | 87.69% |
| Epiretinal macular membrane | 16.93 (16.21-17.67) | 25.80 (18.46-34.74) | 17.34 (16.76-17.93) | 23.54 (17.26-31.19) | 15.84 (15.05-16.66) | 23.05 (16.54-30.68) | -6.44% | -10.66% |
| Hypertensive retinopathy | 0.49 (0.37-0.63) | 0.56 (0.39-0.75) | 0.49 (0.40-0.60) | 0.50 (0.35-0.65) | 0.81 (0.63-1.01) | 0.92 (0.62-1.25) | 65.31% | 64.29% |
| Myelinated fibers | 5.48 (5.08-5.92) | 5.53 (4.77-6.31) | 5.26 (4.94-5.60) | 5.19 (4.52-5.91) | 5.52 (5.06-6.02) | 5.52 (4.72-6.30) | 0.73% | -0.18% |
| Retinitis pigmentosa | 0.28 (0.20-0.39) | 0.36 (0.18-0.60) | 0.34 (0.26-0.43) | 0.29 (0.21-0.38) | 0.27 (0.17-0.40) | 0.31 (0.17-0.46) | -3.57% | -13.89% |

Crude, crude prevalence per 1000 participants; Adjusted, age and sex-adjusted prevalence per 1000 standard population. Data was expressed as the estimate (95% confidence interval)

# Table S21. Prevalence of retinal and optic nerve diseases in Chongqing.

|  | **Prevalence 2019** | | **Prevalence 2020** | | **Prevalence 2021** | | **Percentage change 2019-2021** | |
| --- | --- | --- | --- | --- | --- | --- | --- | --- |
|  | **n=34921** | | **n=13623** | | **n=5725** | |  |  |
|  | Crude | Adjusted | Crude | Adjusted | Crude | Adjusted | Crude | Adjusted |
| Diabetic retinopathy | 11.97 (10.86-13.17) | 10.57 (8.28-13.06) | 10.20 (8.58-12.04) | 13.32 (9.30-18.63) | 9.08 (6.79-11.89) | 8.72 (5.77-12.25) | -24.14% | -17.50% |
| Age-related macular degeneration | 10.62 (9.58-11.76) | 10.93 (8.55-13.66) | 10.72 (9.06-12.59) | 11.75 (9.08-14.74) | 11.70 (9.08-14.84) | 12.09 (7.93-16.95) | 10.17% | 10.61% |
| Referral possible glaucoma | 89.03 (86.06-92.07) | 91.51 (84.24-99.06) | 66.28 (62.16-70.59) | 69.95 (60.89-79.93) | 64.28 (58.06-70.94) | 69.78 (60.78-78.96) | -27.80% | -23.75% |
| Pathological myopia | 8.08 (7.16-9.07) | 9.57 (7.61-11.88) | 9.62 (8.05-11.40) | 11.72 (8.53-15.03) | 12.58 (9.85-15.81) | 15.83 (11.06-21.69) | 55.69% | 65.41% |
| Retinal vein occlusion | 0.69 (0.44-1.02) | 0.80 (0.41-1.25) | 0.88 (0.46-1.54) | 1.05 (0.43-1.78) | 1.05 (0.38-2.28) | 1.15 (0.23-2.48) | 52.17% | 43.75% |
| Macula hole | 0.11 (0.03-0.29) | 0.16 (0.03-0.33) | 0.59 (0.25-1.16) | 0.60 (0.22-1.04) | 1.05 (0.38-2.28) | 0.84 (0.23-1.54) | 854.55% | 425.00% |
| Epiretinal macular membrane | 17.50 (16.15-18.93) | 25.73 (18.25-34.72) | 19.01 (16.79-21.45) | 24.27 (17.06-32.81) | 16.94 (13.76-20.63) | 21.82 (13.93-31.88) | -3.20% | -15.20% |
| Hypertensive retinopathy | 0.43 (0.24-0.71) | 0.45 (0.16-0.80) | 0.15 (0.02-0.53) | 0.16 (0.00-0.41) | 0.52 (0.11-1.53) | 0.63 (0.00-1.44) | 20.93% | 40.00% |
| Myelinated fibers | 7.02 (6.17-7.95) | 6.15 (5.13-7.17) | 6.90 (5.58-8.44) | 6.39 (4.58-8.46) | 9.08 (6.79-11.89) | 9.63 (7.03-12.34) | 29.34% | 56.59% |
| Retinitis pigmentosa | 0.31 (0.16-0.56) | 0.33 (0.14-0.55) | 0.51 (0.21-1.06) | 0.41 (0.13-0.76) | 0.35 (0.04-1.26) | 0.25 (0.00-0.64) | 12.90% | -24.24% |

Crude, crude prevalence per 1000 participants; Adjusted, age and sex-adjusted prevalence per 1000 standard population. Data was expressed as the estimate (95% confidence interval)

# Table S22. Prevalence of retinal and optic nerve diseases in Fujian.

|  | **Prevalence 2019** | | **Prevalence 2020** | | **Prevalence 2021** | | **Percentage change 2019-2021** | |
| --- | --- | --- | --- | --- | --- | --- | --- | --- |
|  | **n=5429** | | **n=4420** | | **n=2842** | |  |  |
|  | Crude | Adjusted | Crude | Adjusted | Crude | Adjusted | Crude | Adjusted |
| Diabetic retinopathy | 4.79 (3.13-7.01) | 7.22 (4.13-10.86) | 8.37 (5.90-11.52) | 12.30 (7.10-18.20) | 8.09 (5.14-12.12) | 10.38 (5.45-16.52) | 68.89% | 43.77% |
| Age-related macular degeneration | 8.47 (6.21-11.29) | 15.15 (9.12-21.95) | 11.31 (8.41-14.89) | 12.34 (7.87-17.42) | 12.67 (8.89-17.49) | 15.56 (8.47-24.44) | 49.59% | 2.71% |
| Referral possible glaucoma | 86.57 (79.22-94.37) | 90.70 (77.91-103.28) | 66.74 (59.56-74.50) | 69.37 (58.04-80.56) | 72.13 (62.89-82.27) | 83.53 (69.09-99.15) | -16.68% | -7.91% |
| Pathological myopia | 3.32 (1.97-5.23) | 5.21 (2.17-9.44) | 5.66 (3.66-8.34) | 7.52 (3.90-12.57) | 3.87 (1.93-6.91) | 3.41 (1.45-5.78) | 16.57% | -34.55% |
| Retinal vein occlusion | 0.00 (0.00-0.68) | 0.00 (0.00-0.00) | 0.90 (0.25-2.32) | 0.85 (0.12-1.81) | 0.70 (0.09-2.54) | 1.01 (0.00-2.70) | NA | NA |
| Macula hole | 0.18 (0.00-1.03) | 0.19 (0.00-0.60) | 0.45 (0.05-1.63) | 0.72 (0.00-2.10) | 0.35 (0.01-1.96) | 0.23 (0.00-0.76) | 94.44% | 21.05% |
| Epiretinal macular membrane | 14.37 (11.37-17.90) | 27.42 (17.54-39.97) | 16.06 (12.57-20.22) | 26.07 (15.75-39.62) | 15.83 (11.57-21.13) | 20.71 (11.87-32.64) | 10.16% | -24.47% |
| Hypertensive retinopathy | 0.00 (0.00-0.68) | 0.00 (0.00-0.00) | 0.68 (0.14-1.98) | 0.62 (0.00-1.50) | 0.35 (0.01-1.96) | 0.33 (0.00-1.08) | NA | NA |
| Myelinated fibers | 5.58 (5.14-6.05) | 5.55 (5.15-6.04) | 4.98 (4.61-5.37) | 4.95 (4.41-5.51) | 5.44 (4.82-6.11) | 5.44 (4.52-6.20) | -2.51% | -1.98% |
| Retinitis pigmentosa | 0.22 (0.14-0.33) | 0.21 (0.11-0.32) | 0.27 (0.19-0.37) | 0.27 (0.22-0.33) | 0.36 (0.22-0.57) | 0.37 (0.31-0.41) | 63.64% | 76.19% |

Crude, crude prevalence per 1000 participants; Adjusted, age and sex-adjusted prevalence per 1000 standard population. Data was expressed as the estimate (95% confidence interval)

# Table S23. Prevalence of retinal and optic nerve diseases in Guangdong.

|  | **Prevalence 2019** | | **Prevalence 2020** | | **Prevalence 2021** | | **Percentage change 2019-2021** | |
| --- | --- | --- | --- | --- | --- | --- | --- | --- |
|  | **n=91905** | | **n=151275** | | **n=46534** | |  |  |
|  | Crude | Adjusted | Crude | Adjusted | Crude | Adjusted | Crude | Adjusted |
| Diabetic retinopathy | 4.93 (4.49-5.40) | 8.03 (6.15-10.20) | 5.58 (5.21-5.97) | 9.49 (7.24-12.17) | 5.44 (4.79-6.15) | 9.39 (6.92-12.17) | 10.34% | 16.94% |
| Age-related macular degeneration | 8.25 (7.67-8.85) | 13.92 (10.62-17.33) | 7.87 (7.43-8.32) | 13.69 (10.77-16.80) | 7.41 (6.65-8.24) | 14.36 (10.64-18.65) | -10.18% | 3.16% |
| Referral possible glaucoma | 74.25 (72.56-75.96) | 78.72 (72.02-85.59) | 63.35 (62.13-64.59) | 69.94 (64.58-76.18) | 61.18 (59.02-63.40) | 66.77 (60.90-73.54) | -17.60% | -15.18% |
| Pathological myopia | 3.75 (3.37-4.17) | 5.47 (4.31-6.72) | 3.38 (3.09-3.68) | 5.85 (4.70-7.21) | 3.80 (3.26-4.41) | 6.73 (4.78-9.10) | 1.33% | 23.03% |
| Retinal vein occlusion | 0.35 (0.24-0.49) | 0.68 (0.40-1.01) | 0.49 (0.38-0.61) | 0.97 (0.63-1.35) | 0.86 (0.61-1.17) | 1.56 (0.89-2.36) | 145.71% | 129.41% |
| Macula hole | 0.46 (0.33-0.62) | 0.85 (0.50-1.27) | 0.44 (0.34-0.56) | 0.84 (0.55-1.15) | 0.82 (0.58-1.12) | 1.72 (0.90-2.82) | 78.26% | 102.35% |
| Epiretinal macular membrane | 11.88 (11.19-12.60) | 25.12 (17.64-33.58) | 10.59 (10.08-11.12) | 25.77 (18.77-34.67) | 12.01 (11.04-13.04) | 26.00 (18.51-35.71) | 1.09% | 3.50% |
| Hypertensive retinopathy | 0.22 (0.13-0.34) | 0.34 (0.15-0.57) | 0.22 (0.16-0.31) | 0.32 (0.17-0.49) | 0.43 (0.26-0.66) | 0.43 (0.25-0.61) | 95.45% | 26.47% |
| Myelinated fibers | 5.33 (4.87-5.82) | 5.64 (4.53-6.97) | 5.43 (5.07-5.82) | 5.86 (5.11-6.70) | 5.05 (4.43-5.74) | 5.09 (4.17-6.05) | -5.25% | -9.75% |
| Retinitis pigmentosa | 0.35 (0.24-0.49) | 0.30 (0.19-0.42) | 0.37 (0.28-0.48) | 0.45 (0.29-0.63) | 0.37 (0.21-0.58) | 0.33 (0.14-0.55) | 5.71% | 10.00% |

Crude, crude prevalence per 1000 participants; Adjusted, age and sex-adjusted prevalence per 1000 standard population. Data was expressed as the estimate (95% confidence interval)

# Table S24. Prevalence of retinal and optic nerve diseases in Guizhou.

|  | **Prevalence 2019** | | **Prevalence 2020** | | **Prevalence 2021** | | **Percentage change 2019-2021** | |
| --- | --- | --- | --- | --- | --- | --- | --- | --- |
|  | **n=30175** | | **n=39960** | | **n=10004** | |  |  |
|  | Crude | Adjusted | Crude | Adjusted | Crude | Adjusted | Crude | Adjusted |
| Diabetic retinopathy | 15.24 (13.89-16.69) | 15.57 (12.02-19.35) | 14.71 (13.56-15.94) | 15.94 (12.28-19.76) | 9.80 (7.96-11.93) | 12.88 (8.59-18.10) | -35.70% | -17.28% |
| Age-related macular degeneration | 10.94 (9.79-12.17) | 12.12 (9.28-15.32) | 10.14 (9.18-11.17) | 11.69 (8.91-14.72) | 9.30 (7.51-11.38) | 12.36 (8.68-16.69) | -14.99% | 1.98% |
| Referral possible glaucoma | 69.59 (66.75-72.52) | 67.74 (59.34-76.05) | 54.95 (52.74-57.23) | 51.80 (45.43-58.12) | 52.38 (48.09-56.93) | 60.91 (49.95-78.00) | -24.73% | -10.08% |
| Pathological myopia | 6.13 (5.28-7.08) | 6.91 (5.25-8.84) | 6.43 (5.67-7.26) | 7.68 (5.90-9.66) | 6.30 (4.84-8.05) | 8.03 (5.39-11.15) | 2.77% | 16.21% |
| Retinal vein occlusion | 1.52 (1.12-2.03) | 2.19 (1.27-3.41) | 1.65 (1.28-2.10) | 2.33 (1.62-3.24) | 1.60 (0.91-2.60) | 3.46 (1.09-6.95) | 5.26% | 57.99% |
| Macula hole | 0.60 (0.35-0.94) | 0.74 (0.32-1.24) | 0.80 (0.55-1.13) | 1.28 (0.66-2.12) | 0.50 (0.16-1.17) | 1.10 (0.23-2.35) | -16.67% | 48.65% |
| Epiretinal macular membrane | 14.95 (13.61-16.38) | 23.70 (16.65-32.59) | 11.61 (10.58-12.71) | 20.25 (14.24-27.50) | 10.60 (8.68-12.80) | 22.75 (14.49-32.68) | -29.10% | -4.01% |
| Hypertensive retinopathy | 1.19 (0.84-1.65) | 1.44 (0.91-2.07) | 1.13 (0.82-1.51) | 1.15 (0.79-1.54) | 1.40 (0.77-2.35) | 1.71 (0.67-3.12) | 17.65% | 18.75% |
| Myelinated fibers | 6.23 (5.37-7.18) | 8.73 (4.33-16.97) | 6.58 (5.81-7.42) | 5.88 (4.83-6.90) | 5.80 (4.41-7.49) | 5.33 (3.98-6.81) | -6.90% | -38.95% |
| Retinitis pigmentosa | 0.40 (0.21-0.69) | 0.27 (0.11-0.44) | 0.35 (0.19-0.59) | 0.33 (0.15-0.58) | 0.40 (0.11-1.02) | 0.33 (0.00-0.74) | 0.00% | 22.22% |

Crude, crude prevalence per 1000 participants; Adjusted, age and sex-adjusted prevalence per 1000 standard population. Data was expressed as the estimate (95% confidence interval)

# Table S25. Prevalence of retinal and optic nerve diseases in Hubei

|  | **Prevalence 2019** | | **Prevalence 2020** | | **Prevalence 2021** | | **Percentage change 2019-2021** | |
| --- | --- | --- | --- | --- | --- | --- | --- | --- |
|  | **n=24894** | | **n=14603** | | **n=6418** | |  |  |
|  | Crude | Adjusted | Crude | Adjusted | Crude | Adjusted | Crude | Adjusted |
| Diabetic retinopathy | 7.07 (6.07-8.19) | 9.09 (6.72-11.59) | 9.11 (7.63-10.78) | 12.37 (8.32-17.01) | 7.95 (5.92-10.43) | 8.58 (5.00-12.54) | 12.45% | -5.61% |
| Age-related macular degeneration | 10.81 (9.56-12.17) | 15.12 (11.74-18.89) | 8.77 (7.32-10.41) | 12.29 (8.36-17.40) | 11.84 (9.34-14.80) | 14.83 (10.11-19.61) | 9.53% | -1.92% |
| Referral possible glaucoma | 61.18 (58.23-64.23) | 63.78 (57.20-70.79) | 55.26 (51.61-59.09) | 59.20 (52.11-67.00) | 64.97 (59.06-71.28) | 68.03 (59.80-76.99) | 6.19% | 6.66% |
| Pathological myopia | 6.47 (5.51-7.54) | 9.15 (7.04-11.85) | 6.03 (4.84-7.42) | 9.18 (6.32-12.45) | 6.70 (4.85-9.01) | 8.18 (4.99-12.60) | 3.55% | -10.60% |
| Retinal vein occlusion | 0.68 (0.40-1.09) | 1.10 (0.48-1.93) | 1.44 (0.89-2.20) | 2.50 (1.15-4.14) | 2.03 (1.08-3.46) | 2.41 (1.09-3.99) | 198.53% | 119.09% |
| Macula hole | 0.48 (0.25-0.84) | 0.92 (0.43-1.54) | 0.55 (0.24-1.08) | 1.03 (0.24-2.02) | 0.78 (0.25-1.82) | 0.58 (0.05-1.36) | 62.50% | -36.96% |
| Epiretinal macular membrane | 13.70 (12.29-15.22) | 23.83 (15.89-33.00) | 10.20 (8.64-11.97) | 20.87 (13.91-29.17) | 15.89 (12.98-19.26) | 23.04 (14.70-33.32) | 15.99% | -3.32% |
| Hypertensive retinopathy | 0.36 (0.17-0.69) | 0.56 (0.17-1.07) | 0.41 (0.15-0.89) | 0.67 (0.13-1.54) | 1.25 (0.54-2.45) | 1.45 (0.52-2.60) | 247.22% | 158.93% |
| Myelinated fibers | 6.91 (5.92-8.02) | 7.27 (5.69-8.92) | 4.79 (3.74-6.05) | 5.02 (3.51-6.72) | 5.61 (3.93-7.76) | 5.48 (3.64-7.37) | -18.81% | -24.62% |
| Retinitis pigmentosa | 0.32 (0.14-0.63) | 0.28 (0.07-0.53) | 0.48 (0.19-0.99) | 0.64 (0.12-1.54) | 0.62 (0.17-1.59) | 0.51 (0.10-1.03) | 93.75% | 82.14% |

Crude, crude prevalence per 1000 participants; Adjusted, age and sex-adjusted prevalence per 1000 standard population. Data was expressed as the estimate (95% confidence interval)

# Table S26. Prevalence of retinal and optic nerve diseases in Hunan.

|  | **Prevalence 2019** | | **Prevalence 2020** | | **Prevalence 2021** | | **Percentage change 2019-2021** | |
| --- | --- | --- | --- | --- | --- | --- | --- | --- |
|  | **n=12503** | | **n=11266** | | **n=6828** | |  |  |
|  | Crude | Adjusted | Crude | Adjusted | Crude | Adjusted | Crude | Adjusted |
| Diabetic retinopathy | 7.04 (5.65-8.66) | 8.34 (5.70-11.34) | 7.99 (6.43-9.81) | 9.71 (6.51-13.39) | 7.62 (5.69-9.98) | 8.61 (5.18-12.70) | 8.24% | 3.24% |
| Age-related macular degeneration | 9.76 (8.11-11.64) | 14.56 (9.51-21.36) | 10.03 (8.27-12.05) | 13.11 (9.23-17.89) | 7.47 (5.57-9.81) | 7.87 (5.25-10.95) | -23.46% | -45.95% |
| Referral possible glaucoma | 82.46 (77.70-87.42) | 85.15 (75.44-95.93) | 62.76 (58.35-67.39) | 69.54 (58.56-83.03) | 70.01 (64.07-76.32) | 69.40 (59.37-79.45) | -15.10% | -18.50% |
| Pathological myopia | 4.56 (3.45-5.90) | 5.01 (3.52-6.56) | 6.04 (4.69-7.65) | 8.07 (5.32-11.91) | 4.10 (2.73-5.92) | 4.68 (2.87-6.73) | -10.09% | -6.59% |
| Retinal vein occlusion | 0.72 (0.33-1.37) | 1.08 (0.31-2.28) | 1.07 (0.55-1.86) | 1.57 (0.68-2.80) | 1.17 (0.51-2.31) | 2.10 (0.37-5.17) | 62.50% | 94.44% |
| Macula hole | 0.24 (0.05-0.70) | 0.45 (0.00-1.16) | 0.89 (0.43-1.63) | 3.09 (0.41-7.52) | 0.73 (0.24-1.71) | 1.53 (0.10-4.10) | 204.17% | 240.00% |
| Epiretinal macular membrane | 12.96 (11.05-15.10) | 21.62 (14.64-30.38) | 14.02 (11.94-16.37) | 22.47 (15.40-30.50) | 13.47 (10.88-16.50) | 21.48 (12.43-31.85) | 3.94% | -0.65% |
| Hypertensive retinopathy | 0.16 (0.02-0.58) | 0.12 (0.00-0.38) | 0.27 (0.05-0.78) | 0.20 (0.00-0.47) | 0.29 (0.04-1.06) | 0.55 (0.00-1.53) | 81.25% | 358.33% |
| Myelinated fibers | 6.00 (4.72-7.51) | 6.98 (3.90-11.33) | 6.39 (5.00-8.04) | 6.74 (4.70-9.20) | 7.62 (5.69-9.98) | 7.22 (4.84-10.11) | 27.00% | 3.44% |
| Retinitis pigmentosa | 0.16 (0.02-0.58) | 0.13 (0.00-0.40) | 0.18 (0.02-0.64) | 0.13 (0.00-0.35) | 0.29 (0.04-1.06) | 0.26 (0.00-0.67) | 81.25% | 100.00% |

Crude, crude prevalence per 1000 participants; Adjusted, age and sex-adjusted prevalence per 1000 standard population. Data was expressed as the estimate (95% confidence interval)

# Table S27. Prevalence of retinal and optic nerve diseases in aged Jiangsu

|  | **Prevalence 2019** | | **Prevalence 2020** | | **Prevalence 2021** | | **Percentage change 2019-2021** | |
| --- | --- | --- | --- | --- | --- | --- | --- | --- |
|  | **n=64904** | | **n=103625** | | **n=37354** | |  |  |
|  | Crude | Adjusted | Crude | Adjusted | Crude | Adjusted | Crude | Adjusted |
| Diabetic retinopathy | 9.38 (8.66-10.15) | 13.10 (10.16-16.41) | 9.85 (9.26-10.47) | 12.69 (10.11-15.60) | 11.27 (10.22-12.39) | 12.48 (9.64-15.27) | 20.15% | -4.73% |
| Age-related macular degeneration | 13.36 (12.49-14.27) | 19.84 (15.56-24.62) | 13.94 (13.24-14.68) | 19.09 (14.90-23.49) | 15.02 (13.81-16.30) | 17.81 (13.95-22.12) | 12.43% | -10.23% |
| Referral possible glaucoma | 69.92 (67.97-71.91) | 79.74 (69.81-93.14) | 62.29 (60.83-63.78) | 67.44 (61.92-73.81) | 64.97 (62.49-67.52) | 68.52 (62.44-74.90) | -7.08% | -14.07% |
| Pathological myopia | 4.55 (4.04-5.09) | 6.70 (5.21-8.40) | 5.22 (4.79-5.68) | 7.38 (5.92-9.02) | 5.49 (4.76-6.29) | 6.71 (5.19-8.60) | 20.66% | 0.15% |
| Retinal vein occlusion | 0.45 (0.30-0.64) | 0.70 (0.40-1.06) | 1.01 (0.83-1.23) | 1.56 (1.07-2.10) | 1.58 (1.20-2.04) | 1.96 (1.08-3.08) | 251.11% | 180.00% |
| Macula hole | 0.35 (0.22-0.53) | 0.74 (0.33-1.23) | 0.67 (0.52-0.84) | 1.10 (0.68-1.59) | 0.56 (0.35-0.86) | 0.76 (0.36-1.28) | 60.00% | 2.70% |
| Epiretinal macular membrane | 12.87 (12.01-13.76) | 26.16 (18.67-34.92) | 13.76 (13.06-14.49) | 24.06 (17.10-31.98) | 17.35 (16.05-18.72) | 24.93 (17.27-34.32) | 34.81% | -4.70% |
| Hypertensive retinopathy | 0.49 (0.34-0.70) | 0.53 (0.31-0.77) | 0.45 (0.33-0.60) | 0.59 (0.36-0.89) | 0.78 (0.52-1.11) | 0.76 (0.43-1.15) | 59.18% | 43.40% |
| Myelinated fibers | 6.87 (6.25-7.54) | 6.88 (5.83-7.99) | 6.99 (6.49-7.51) | 6.99 (6.08-8.00) | 6.26 (5.49-7.12) | 5.73 (4.83-6.61) | -8.88% | -16.72% |
| Retinitis pigmentosa | 0.35 (0.22-0.53) | 0.35 (0.21-0.53) | 0.36 (0.25-0.49) | 0.45 (0.25-0.70) | 0.48 (0.29-0.76) | 0.45 (0.21-0.74) | 37.14% | 28.57% |

Crude, crude prevalence per 1000 participants; Adjusted, age and sex-adjusted prevalence per 1000 standard population. Data was expressed as the estimate (95% confidence interval)

# Table S28. Prevalence of retinal and optic nerve diseases in Liaoning.

|  | **Prevalence 2019** | | **Prevalence 2020** | | **Prevalence 2021** | | **Percentage change 2019-2021** | |
| --- | --- | --- | --- | --- | --- | --- | --- | --- |
|  | **n=8021** | | **n=7058** | | **n=5263** | |  |  |
|  | Crude | Adjusted | Crude | Adjusted | Crude | Adjusted | Crude | Adjusted |
| Diabetic retinopathy | 10.22 (8.14-12.67) | 15.85 (10.61-21.98) | 19.55 (16.45-23.06) | 21.97 (15.38-29.01) | 19.76 (16.17-23.89) | 20.37 (14.59-26.59) | 93.35% | 28.52% |
| Age-related macular degeneration | 11.59 (9.37-14.19) | 17.91 (12.58-24.44) | 19.84 (16.71-23.36) | 22.26 (16.55-29.12) | 17.67 (14.29-21.60) | 17.03 (11.78-23.19) | 52.46% | -4.91% |
| Referral possible glaucoma | 78.92 (73.11-85.03) | 89.80 (79.52-100.10) | 64.61 (58.98-70.60) | 66.17 (57.68-74.79) | 68.40 (61.73-75.56) | 76.33 (62.47-94.27) | -13.33% | -15.00% |
| Pathological myopia | 3.37 (2.22-4.89) | 4.97 (2.51-8.32) | 4.68 (3.22-6.56) | 6.90 (3.47-11.97) | 5.70 (3.85-8.13) | 7.51 (4.05-11.82) | 69.14% | 51.11% |
| Retinal vein occlusion | 0.87 (0.35-1.80) | 1.11 (0.38-2.04) | 2.55 (1.51-4.03) | 2.58 (1.12-4.34) | 3.61 (2.17-5.63) | 3.09 (1.61-4.72) | 314.94% | 178.38% |
| Macula hole | 0.50 (0.14-1.28) | 0.62 (0.09-1.26) | 1.56 (0.78-2.79) | 1.75 (0.44-3.67) | 1.71 (0.78-3.24) | 2.46 (0.77-5.03) | 242.00% | 296.77% |
| Epiretinal macular membrane | 9.48 (7.47-11.85) | 14.84 (9.23-21.74) | 20.40 (17.23-23.98) | 23.01 (14.82-33.08) | 19.76 (16.17-23.89) | 19.70 (12.54-27.67) | 108.44% | 32.75% |
| Hypertensive retinopathy | 0.50 (0.14-1.28) | 0.87 (0.16-1.85) | 1.98 (1.08-3.33) | 2.03 (0.83-3.33) | 1.71 (0.78-3.24) | 1.64 (0.67-2.79) | 242.00% | 88.51% |
| Myelinated fibers | 4.99 (3.57-6.78) | 4.63 (3.13-6.22) | 6.52 (4.78-8.68) | 6.72 (4.77-9.01) | 5.70 (3.85-8.13) | 5.21 (2.78-8.11) | 14.23% | 12.53% |
| Retinitis pigmentosa | 0.50 (0.14-1.28) | 0.38 (0.04-0.82) | 0.28 (0.03-1.02) | 0.22 (0.00-0.59) | 0.57 (0.12-1.66) | 0.38 (0.00-1.01) | 14.00% | 0.00% |

Crude, crude prevalence per 1000 participants; Adjusted, age and sex-adjusted prevalence per 1000 standard population. Data was expressed as the estimate (95% confidence interval)

# Table S29. Prevalence of retinal and optic nerve diseases in Ningxia.

|  | **Prevalence 2019** | | **Prevalence 2020** | | **Prevalence 2021** | | **Percentage change 2019-2021** | |
| --- | --- | --- | --- | --- | --- | --- | --- | --- |
|  | **n=14136** | | **n=9918** | | **n=3950** | |  |  |
|  | Crude | Adjusted | Crude | Adjusted | Crude | Adjusted | Crude | Adjusted |
| Diabetic retinopathy | 12.52 (10.75-14.49) | 14.28 (10.08-19.02) | 14.42 (12.17-16.96) | 16.97 (12.89-21.94) | 13.92 (10.51-18.09) | 16.99 (11.02-25.05) | 11.18% | 18.98% |
| Age-related macular degeneration | 13.16 (11.34-15.18) | 16.45 (11.91-22.65) | 16.03 (13.65-18.70) | 23.28 (16.58-31.15) | 14.94 (11.39-19.23) | 17.80 (11.13-24.62) | 13.53% | 8.21% |
| Referral possible glaucoma | 81.21 (76.76-85.84) | 81.03 (71.08-90.81) | 58.08 (53.55-62.86) | 63.36 (55.20-72.93) | 62.53 (55.18-70.54) | 69.08 (56.64-83.58) | -23.00% | -14.75% |
| Pathological myopia | 3.40 (2.50-4.50) | 4.01 (2.56-5.88) | 3.13 (2.12-4.43) | 4.30 (2.34-6.86) | 5.82 (3.69-8.72) | 9.00 (3.76-16.47) | 71.18% | 124.44% |
| Retinal vein occlusion | 1.63 (1.03-2.44) | 1.98 (1.07-3.09) | 3.02 (2.04-4.32) | 3.59 (2.12-5.27) | 4.05 (2.32-6.57) | 4.03 (1.84-6.59) | 148.47% | 103.54% |
| Macula hole | 0.28 (0.08-0.72) | 0.36 (0.04-0.83) | 1.51 (0.85-2.49) | 2.91 (1.02-5.93) | 0.00 (0.00-0.93) | 0.00 (0.00-0.00) | NA | NA |
| Epiretinal macular membrane | 13.02 (11.21-15.02) | 25.22 (16.63-36.15) | 13.31 (11.15-15.76) | 20.82 (14.04-29.35) | 16.46 (12.72-20.93) | 25.06 (14.96-36.88) | 26.42% | -0.63% |
| Hypertensive retinopathy | 0.35 (0.11-0.83) | 0.29 (0.03-0.65) | 1.01 (0.48-1.85) | 1.50 (0.40-3.20) | 1.52 (0.56-3.30) | 1.03 (0.31-1.89) | 334.29% | 255.17% |
| Myelinated fibers | 7.29 (5.95-8.83) | 6.77 (5.17-8.42) | 6.86 (5.33-8.68) | 6.34 (4.64-8.00) | 9.62 (6.82-13.18) | 10.38 (6.69-14.63) | 31.96% | 53.32% |
| Retinitis pigmentosa | 0.28 (0.08-0.72) | 0.23 (0.00-0.55) | 0.40 (0.11-1.03) | 0.30 (0.06-0.63) | 0.51 (0.06-1.83) | 0.50 (0.00-1.30) | 82.14% | 117.39% |

Crude, crude prevalence per 1000 participants; Adjusted, age and sex-adjusted prevalence per 1000 standard population. Data was expressed as the estimate (95% confidence interval)

# Table S30. Prevalence of retinal and optic nerve diseases in Shannxi.

|  | **Prevalence 2019** | | **Prevalence 2020** | | **Prevalence 2021** | | **Percentage change 2019-2021** | |
| --- | --- | --- | --- | --- | --- | --- | --- | --- |
|  | **n=9273** | | **n=9990** | | **n=6361** | |  |  |
|  | Crude | Adjusted | Crude | Adjusted | Crude | Adjusted | Crude | Adjusted |
| Diabetic retinopathy | 4.74 (3.45-6.36) | 7.70 (4.45-11.54) | 9.41 (7.61-11.50) | 9.68 (7.26-12.36) | 7.86 (5.84-10.35) | 8.00 (5.20-11.02) | 65.82% | 3.90% |
| Age-related macular degeneration | 9.27 (7.42-11.44) | 13.79 (9.46-18.78) | 11.41 (9.42-13.69) | 13.02 (9.59-16.94) | 11.16 (8.73-14.06) | 12.49 (8.54-17.41) | 20.39% | -9.43% |
| Referral possible glaucoma | 62.01 (57.18-67.11) | 76.82 (61.26-99.77) | 53.25 (48.93-57.84) | 55.86 (48.93-63.31) | 52.19 (46.86-57.95) | 56.63 (47.73-66.61) | -15.84% | -26.28% |
| Pathological myopia | 4.74 (3.45-6.36) | 7.22 (4.36-11.16) | 6.61 (5.11-8.40) | 8.23 (5.96-10.70) | 7.07 (5.16-9.45) | 6.78 (4.53-9.37) | 49.16% | -6.09% |
| Retinal vein occlusion | 0.65 (0.24-1.41) | 1.87 (0.32-3.84) | 2.40 (1.54-3.57) | 3.92 (1.58-7.14) | 2.52 (1.44-4.08) | 3.20 (1.45-5.32) | 287.69% | 71.12% |
| Macula hole | 0.00 (0.00-0.40) | 0.00 (0.00-0.00) | 0.60 (0.22-1.31) | 0.48 (0.14-0.90) | 0.31 (0.04-1.14) | 0.23 (0.00-0.59) | NA | NA |
| Epiretinal macular membrane | 8.09 (6.37-10.13) | 16.69 (10.80-23.69) | 13.91 (11.71-16.41) | 19.34 (12.36-27.08) | 12.26 (9.70-15.28) | 19.24 (11.91-28.26) | 51.55% | 15.28% |
| Hypertensive retinopathy | 0.43 (0.12-1.10) | 0.91 (0.12-2.10) | 0.50 (0.16-1.17) | 0.46 (0.10-0.93) | 1.10 (0.44-2.27) | 1.89 (0.22-4.92) | 155.81% | 107.69% |
| Myelinated fibers | 4.21 (2.99-5.74) | 3.39 (2.31-4.47) | 5.71 (4.32-7.39) | 5.33 (3.89-6.82) | 7.07 (5.16-9.45) | 7.02 (4.36-10.43) | 67.93% | 107.08% |
| Retinitis pigmentosa | 0.22 (0.03-0.78) | 0.17 (0.00-0.44) | 1.00 (0.48-1.84) | 0.74 (0.27-1.28) | 0.16 (0.00-0.88) | 0.14 (0.00-0.44) | -27.27% | -17.65% |

Crude, crude prevalence per 1000 participants; Adjusted, age and sex-adjusted prevalence per 1000 standard population. Data was expressed as the estimate (95% confidence interval)

# Table S31. Prevalence of retinal and optic nerve diseases in Shandong.

|  | **Prevalence 2019** | | **Prevalence 2020** | | **Prevalence 2021** | | **Percentage change 2019-2021** | |
| --- | --- | --- | --- | --- | --- | --- | --- | --- |
|  | **n=49093** | | **n=45209** | | **n=19518** | |  |  |
|  | Crude | Adjusted | Crude | Adjusted | Crude | Adjusted | Crude | Adjusted |
| Diabetic retinopathy | 13.77 (12.76-14.84) | 14.51 (11.46-17.99) | 17.81 (16.61-19.07) | 17.14 (13.35-21.15) | 16.19 (14.47-18.06) | 15.46 (11.50-19.46) | 17.57% | 6.55% |
| Age-related macular degeneration | 15.54 (14.47-16.68) | 17.59 (13.51-22.03) | 16.15 (15.01-17.35) | 17.52 (13.34-22.16) | 15.01 (13.35-16.82) | 15.17 (11.44-19.29) | -3.41% | -13.76% |
| Referral possible glaucoma | 74.43 (72.12-76.79) | 83.03 (70.17-103.52) | 69.08 (66.76-71.46) | 76.82 (65.04-91.51) | 77.67 (73.95-81.52) | 77.30 (68.99-86.04) | 4.35% | -6.90% |
| Pathological myopia | 2.63 (2.19-3.12) | 2.67 (2.04-3.41) | 3.01 (2.52-3.56) | 2.99 (2.27-3.82) | 3.07 (2.35-3.96) | 3.77 (2.47-5.34) | 16.73% | 41.20% |
| Retinal vein occlusion | 1.06 (0.79-1.39) | 1.33 (0.76-2.10) | 2.10 (1.70-2.57) | 2.11 (1.47-2.89) | 2.10 (1.51-2.85) | 1.98 (1.15-3.00) | 98.11% | 48.87% |
| Macula hole | 0.71 (0.50-0.99) | 0.72 (0.42-1.09) | 0.84 (0.59-1.15) | 1.06 (0.60-1.65) | 1.38 (0.91-2.01) | 1.49 (0.79-2.28) | 94.37% | 106.94% |
| Epiretinal macular membrane | 18.58 (17.40-19.81) | 22.54 (16.14-30.68) | 22.43 (21.08-23.84) | 26.73 (18.99-34.84) | 22.08 (20.07-24.24) | 24.64 (16.70-34.32) | 18.84% | 9.32% |
| Hypertensive retinopathy | 0.90 (0.65-1.20) | 0.90 (0.58-1.27) | 1.17 (0.88-1.53) | 1.05 (0.70-1.45) | 1.38 (0.91-2.01) | 1.39 (0.82-2.00) | 53.33% | 54.44% |
| Myelinated fibers | 5.60 (4.96-6.30) | 4.86 (4.17-5.60) | 6.37 (5.66-7.15) | 5.39 (4.45-6.23) | 4.92 (3.99-6.00) | 5.81 (3.41-10.00) | -12.14% | 19.55% |
| Retinitis pigmentosa | 0.51 (0.33-0.75) | 0.47 (0.29-0.68) | 0.24 (0.12-0.44) | 0.24 (0.09-0.44) | 0.61 (0.32-1.07) | 0.53 (0.25-0.82) | 19.61% | 12.77% |

Crude, crude prevalence per 1000 participants; Adjusted, age and sex-adjusted prevalence per 1000 standard population. Data was expressed as the estimate (95% confidence interval)

# Table S32. Prevalence of retinal and optic nerve diseases in Shanghai.

|  | **Prevalence 2019** | | **Prevalence 2020** | | **Prevalence 2021** | | **Percentage change 2019-2021** | |
| --- | --- | --- | --- | --- | --- | --- | --- | --- |
|  | **n=81433** | | **n=143181** | | **n=57652** | |  |  |
|  | Crude | Adjusted | Crude | Adjusted | Crude | Adjusted | Crude | Adjusted |
| Diabetic retinopathy | 8.63 (8.01-9.29) | 10.94 (8.55-13.52) | 9.83 (9.33-10.36) | 11.61 (9.02-14.21) | 9.47 (8.70-10.30) | 10.54 (8.23-13.13) | 9.73% | -3.66% |
| Age-related macular degeneration | 12.99 (12.23-13.79) | 16.75 (13.43-20.52) | 12.42 (11.86-13.01) | 15.24 (11.93-18.95) | 11.15 (10.31-12.04) | 12.93 (10.01-16.02) | -14.16% | -22.81% |
| Referral possible glaucoma | 79.82 (77.97-81.70) | 84.28 (77.87-91.09) | 73.64 (72.29-75.01) | 77.36 (71.56-84.10) | 71.12 (69.03-73.24) | 77.00 (66.08-92.57) | -10.90% | -8.64% |
| Pathological myopia | 9.17 (8.53-9.85) | 12.11 (9.78-14.89) | 10.01 (9.50-10.54) | 13.34 (10.59-16.84) | 9.18 (8.41-9.99) | 11.71 (9.06-15.24) | 0.11% | -3.30% |
| Retinal vein occlusion | 0.77 (0.59-0.99) | 1.04 (0.69-1.44) | 1.15 (0.98-1.33) | 1.50 (1.08-1.97) | 1.49 (1.19-1.84) | 1.68 (1.14-2.26) | 93.51% | 61.54% |
| Macula hole | 0.45 (0.32-0.63) | 0.56 (0.34-0.80) | 1.06 (0.90-1.24) | 1.28 (0.88-1.73) | 1.18 (0.92-1.50) | 1.34 (0.86-1.91) | 162.22% | 139.29% |
| Epiretinal macular membrane | 19.83 (18.89-20.81) | 27.36 (19.19-36.47) | 21.32 (20.58-22.08) | 26.50 (19.42-35.00) | 23.33 (22.11-24.59) | 25.70 (18.28-34.49) | 17.65% | -6.07% |
| Hypertensive retinopathy | 0.29 (0.19-0.44) | 0.36 (0.20-0.56) | 0.44 (0.34-0.56) | 0.55 (0.38-0.75) | 0.59 (0.41-0.82) | 0.59 (0.37-0.83) | 103.45% | 63.89% |
| Myelinated fibers | 5.86 (5.34-6.41) | 5.63 (4.90-6.42) | 6.14 (5.74-6.56) | 5.82 (5.14-6.46) | 6.83 (6.18-7.54) | 6.39 (5.49-7.35) | 16.55% | 13.50% |
| Retinitis pigmentosa | 0.33 (0.22-0.48) | 0.34 (0.20-0.48) | 0.34 (0.25-0.45) | 0.34 (0.24-0.44) | 0.43 (0.28-0.64) | 0.40 (0.22-0.66) | 30.30% | 17.65% |

Crude, crude prevalence per 1000 participants; Adjusted, age and sex-adjusted prevalence per 1000 standard population. Data was expressed as the estimate (95% confidence interval)

# Table S33. Prevalence of retinal and optic nerve diseases in Sichuan.

|  | **Prevalence 2019** | | **Prevalence 2020** | | **Prevalence 2021** | | **Percentage change 2019-2021** | |
| --- | --- | --- | --- | --- | --- | --- | --- | --- |
|  | **n=37360** | | **n=34866** | | **n=20424** | |  |  |
|  | Crude | Adjusted | Crude | Adjusted | Crude | Adjusted | Crude | Adjusted |
| Diabetic retinopathy | 8.97 (8.04-9.98) | 11.70 (9.17-14.76) | 9.64 (8.64-10.72) | 12.45 (9.55-15.81) | 7.25 (6.13-8.51) | 9.47 (6.88-12.47) | -19.18% | -19.06% |
| Age-related macular degeneration | 11.94 (10.86-13.09) | 15.79 (12.34-19.91) | 11.19 (10.11-12.35) | 15.38 (11.73-19.62) | 7.05 (5.95-8.30) | 10.63 (7.56-14.29) | -40.95% | -32.68% |
| Referral possible glaucoma | 71.07 (68.48-73.72) | 78.04 (70.47-86.29) | 62.07 (59.56-64.65) | 69.05 (61.29-78.10) | 64.83 (61.49-68.29) | 74.38 (66.75-82.86) | -8.78% | -4.69% |
| Pathological myopia | 8.32 (7.43-9.30) | 10.74 (8.54-13.50) | 8.78 (7.82-9.81) | 14.05 (10.33-18.67) | 6.51 (5.46-7.71) | 8.93 (6.82-11.32) | -21.75% | -16.85% |
| Retinal vein occlusion | 0.54 (0.33-0.83) | 0.78 (0.35-1.34) | 0.92 (0.63-1.30) | 1.04 (0.61-1.52) | 0.98 (0.60-1.51) | 2.22 (0.98-3.91) | 81.48% | 184.62% |
| Macula hole | 0.51 (0.31-0.79) | 0.61 (0.32-0.95) | 0.72 (0.46-1.06) | 1.13 (0.61-1.79) | 0.73 (0.41-1.21) | 1.02 (0.51-1.59) | 43.14% | 67.21% |
| Epiretinal macular membrane | 15.44 (14.22-16.75) | 25.05 (17.83-33.57) | 14.48 (13.26-15.79) | 23.73 (16.94-31.69) | 12.09 (10.64-13.69) | 23.51 (15.64-33.02) | -21.70% | -6.15% |
| Hypertensive retinopathy | 0.43 (0.24-0.70) | 0.59 (0.31-0.94) | 0.77 (0.51-1.13) | 0.92 (0.53-1.38) | 0.59 (0.30-1.03) | 0.54 (0.24-0.91) | 37.21% | -8.47% |
| Myelinated fibers | 6.93 (6.12-7.83) | 6.63 (5.57-7.66) | 7.69 (6.80-8.66) | 7.83 (6.59-9.19) | 7.05 (5.95-8.30) | 7.43 (5.85-9.24) | 1.73% | 12.07% |
| Retinitis pigmentosa | 0.56 (0.35-0.86) | 0.61 (0.35-0.90) | 0.40 (0.22-0.67) | 0.38 (0.20-0.58) | 0.49 (0.23-0.90) | 0.56 (0.25-0.97) | -12.50% | -8.20% |

Crude, crude prevalence per 1000 participants; Adjusted, age and sex-adjusted prevalence per 1000 standard population. Data was expressed as the estimate (95% confidence interval)

# Table S34. Prevalence of retinal and optic nerve diseases Tianjin

|  | **Prevalence 2019** | | **Prevalence 2020** | | **Prevalence 2021** | | **Percentage change 2019-2021** | |
| --- | --- | --- | --- | --- | --- | --- | --- | --- |
|  | **n=19540** | | **n=29334** | | **n=10720** | |  |  |
|  | Crude | Adjusted | Crude | Adjusted | Crude | Adjusted | Crude | Adjusted |
| Diabetic retinopathy | 18.99 (17.12-21.00) | 19.31 (15.20-24.06) | 10.09 (8.98-11.30) | 12.74 (10.07-15.60) | 11.29 (9.37-13.47) | 11.65 (8.34-15.09) | -40.55% | -39.67% |
| Age-related macular degeneration | 16.73 (14.98-18.63) | 18.85 (14.35-23.76) | 10.47 (9.33-11.70) | 15.24 (11.15-19.51) | 13.34 (11.25-15.70) | 11.27 (7.94-15.11) | -20.26% | -40.21% |
| Referral possible glaucoma | 83.83 (79.98-87.80) | 81.03 (73.01-89.44) | 67.19 (64.35-70.12) | 71.44 (64.89-78.94) | 73.32 (68.46-78.42) | 70.82 (62.62-79.71) | -12.54% | -12.60% |
| Pathological myopia | 3.99 (3.16-4.98) | 5.14 (3.58-6.95) | 3.48 (2.84-4.22) | 5.37 (3.88-7.16) | 4.29 (3.14-5.72) | 3.96 (2.53-5.63) | 7.52% | -22.96% |
| Retinal vein occlusion | 1.28 (0.83-1.89) | 1.83 (0.99-2.88) | 1.12 (0.77-1.58) | 1.44 (0.83-2.17) | 2.99 (2.04-4.21) | 2.18 (1.25-3.28) | 133.59% | 19.13% |
| Macula hole | 0.72 (0.39-1.20) | 0.96 (0.43-1.65) | 0.75 (0.47-1.14) | 1.55 (0.64-2.75) | 1.03 (0.51-1.84) | 1.32 (0.37-2.52) | 43.06% | 37.50% |
| Epiretinal macular membrane | 17.55 (15.76-19.49) | 24.68 (17.52-32.97) | 15.41 (14.03-16.88) | 26.63 (18.66-35.59) | 22.29 (19.58-25.27) | 26.15 (17.96-36.09) | 27.01% | 5.96% |
| Hypertensive retinopathy | 0.92 (0.55-1.46) | 0.97 (0.47-1.51) | 0.75 (0.47-1.14) | 0.98 (0.49-1.56) | 1.59 (0.92-2.54) | 1.23 (0.65-1.84) | 72.83% | 26.80% |
| Myelinated fibers | 6.81 (5.70-8.06) | 6.40 (5.05-7.92) | 6.24 (5.37-7.21) | 6.11 (4.84-7.34) | 6.90 (5.42-8.66) | 6.15 (4.39-8.20) | 1.32% | -3.91% |
| Retinitis pigmentosa | 0.41 (0.18-0.81) | 0.36 (0.12-0.65) | 0.24 (0.10-0.49) | 0.18 (0.06-0.33) | 0.28 (0.06-0.82) | 0.17 (0.00-0.38) | -31.71% | -52.78% |

Crude, crude prevalence per 1000 participants; Adjusted, age and sex-adjusted prevalence per 1000 standard population. Data was expressed as the estimate (95% confidence interval)

# Table S35. Prevalence of retinal and optic nerve diseases in Zhejiang

|  | **Prevalence 2019** | | **Prevalence 2020** | | **Prevalence 2021** | | **Percentage change 2019-2021** | |
| --- | --- | --- | --- | --- | --- | --- | --- | --- |
|  | **n=38219** | | **n=41956** | | **n=22129** | |  |  |
|  | Crude | Adjusted | Crude | Adjusted | Crude | Adjusted | Crude | Adjusted |
| Diabetic retinopathy | 5.63 (4.90-6.43) | 10.21 (7.54-13.37) | 8.06 (7.22-8.96) | 11.22 (8.21-14.52) | 6.37 (5.37-7.51) | 8.42 (6.26-10.98) | 13.14% | -17.53% |
| Age-related macular degeneration | 8.84 (7.93-9.83) | 15.42 (11.85-19.17) | 11.54 (10.54-12.60) | 17.17 (13.11-21.43) | 10.53 (9.23-11.96) | 14.49 (11.00-18.70) | 19.12% | -6.03% |
| Referral possible glaucoma | 64.84 (62.39-67.35) | 79.66 (70.72-89.13) | 56.56 (54.37-58.81) | 63.40 (57.50-70.10) | 57.12 (54.10-60.26) | 65.02 (57.34-72.77) | -11.91% | -18.38% |
| Pathological myopia | 4.61 (3.95-5.34) | 6.29 (4.78-8.00) | 4.96 (4.31-5.68) | 7.67 (5.45-10.47) | 4.38 (3.56-5.34) | 6.22 (4.26-8.57) | -4.99% | -1.11% |
| Retinal vein occlusion | 0.44 (0.26-0.71) | 0.73 (0.30-1.23) | 0.83 (0.58-1.16) | 1.33 (0.74-2.05) | 0.90 (0.55-1.40) | 1.13 (0.53-1.87) | 104.55% | 54.79% |
| Macula hole | 0.29 (0.14-0.51) | 0.45 (0.17-0.82) | 0.72 (0.48-1.02) | 1.24 (0.68-2.00) | 0.45 (0.22-0.83) | 0.57 (0.19-1.16) | 55.17% | 26.67% |
| Epiretinal macular membrane | 9.79 (8.82-10.82) | 23.34 (15.31-32.47) | 12.58 (11.54-13.70) | 23.63 (16.10-32.68) | 12.07 (10.67-13.59) | 23.77 (16.57-32.35) | 23.29% | 1.84% |
| Hypertensive retinopathy | 0.18 (0.07-0.38) | 0.21 (0.06-0.38) | 0.60 (0.39-0.88) | 0.76 (0.41-1.16) | 0.59 (0.31-1.00) | 0.84 (0.38-1.38) | 227.78% | 300.00% |
| Myelinated fibers | 5.10 (4.41-5.87) | 5.26 (4.29-6.27) | 5.98 (5.27-6.77) | 6.00 (4.92-7.14) | 6.10 (5.12-7.22) | 7.14 (5.08-9.86) | 19.61% | 35.74% |
| Retinitis pigmentosa | 0.31 (0.16-0.55) | 0.32 (0.07-0.66) | 0.43 (0.25-0.68) | 0.52 (0.24-0.87) | 0.54 (0.28-0.95) | 0.64 (0.20-1.28) | 74.19% | 100.00% |

Crude, crude prevalence per 1000 participants; Adjusted, age and sex-adjusted prevalence per 1000 standard population. Data was expressed as the estimate (95% confidence interval)

# Figure S1. Distribution of participants in 2019-2021.


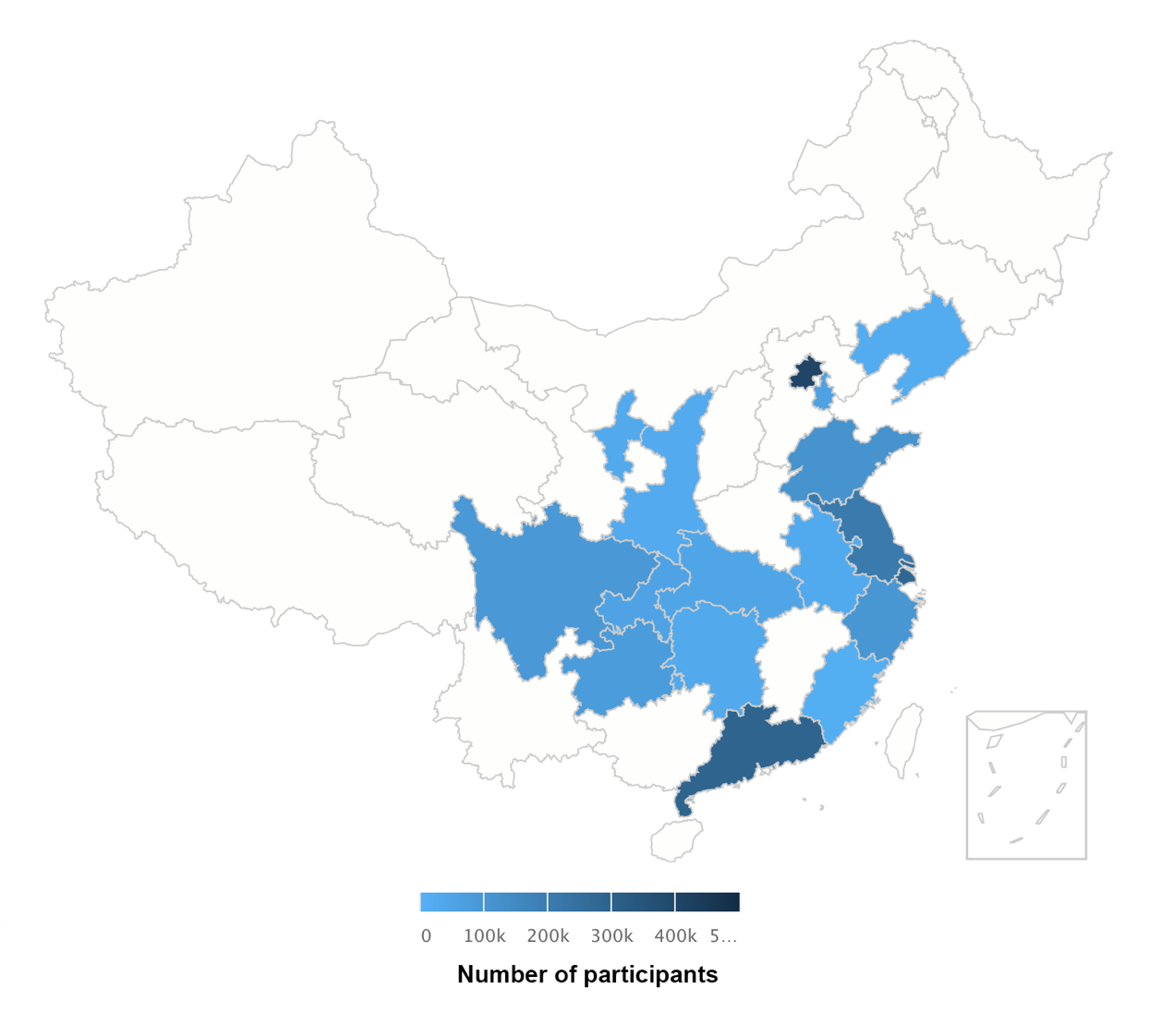


# Figure S2. Prevalence of diabetic retinopathy in 2021 (age and sex-adjusted prevalence per 1000 standard population).


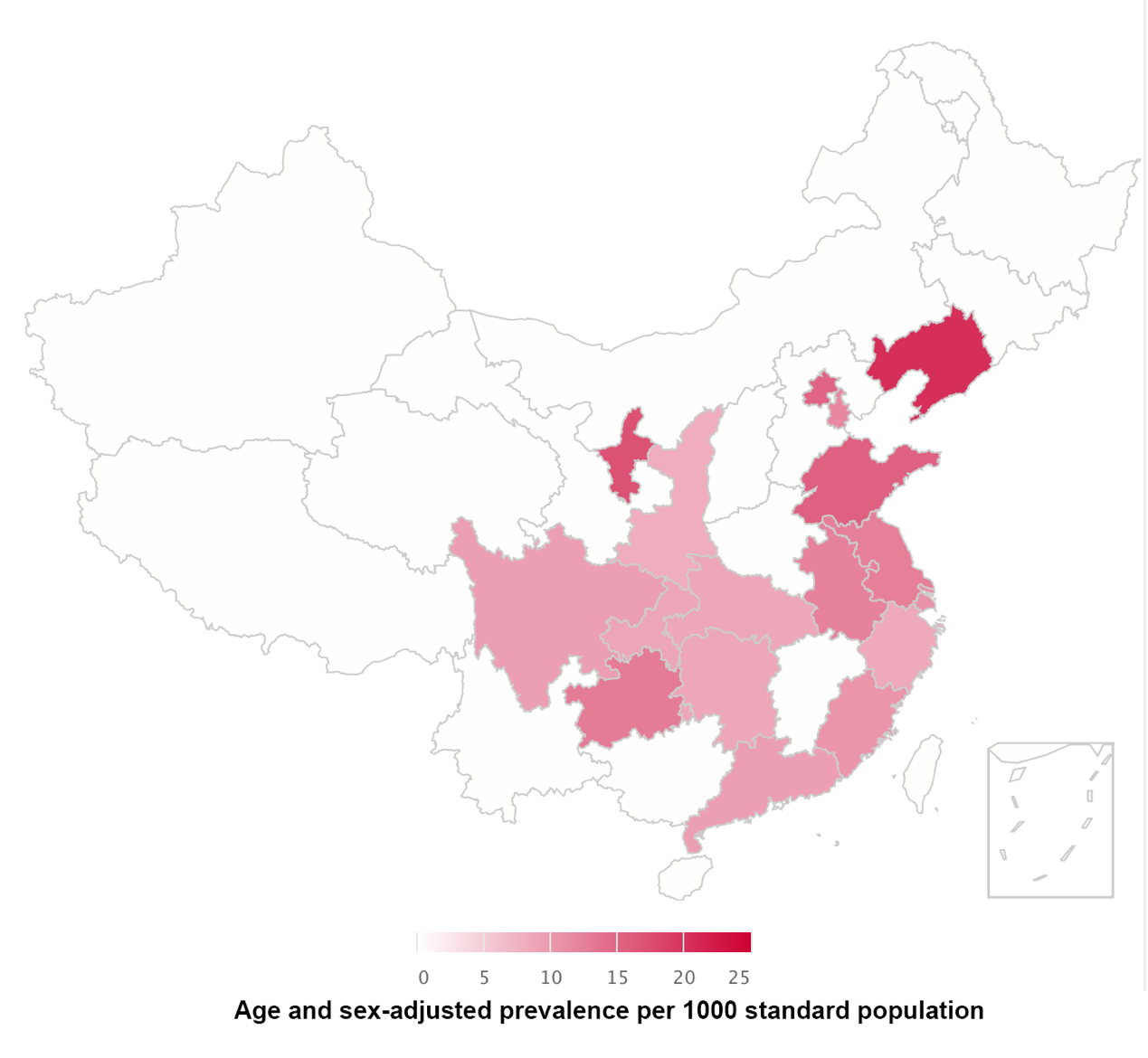


# Figure S3. Prevalence of age-related macular degeneration in 2021 (age and sex-adjusted prevalence per 1000 standard population).


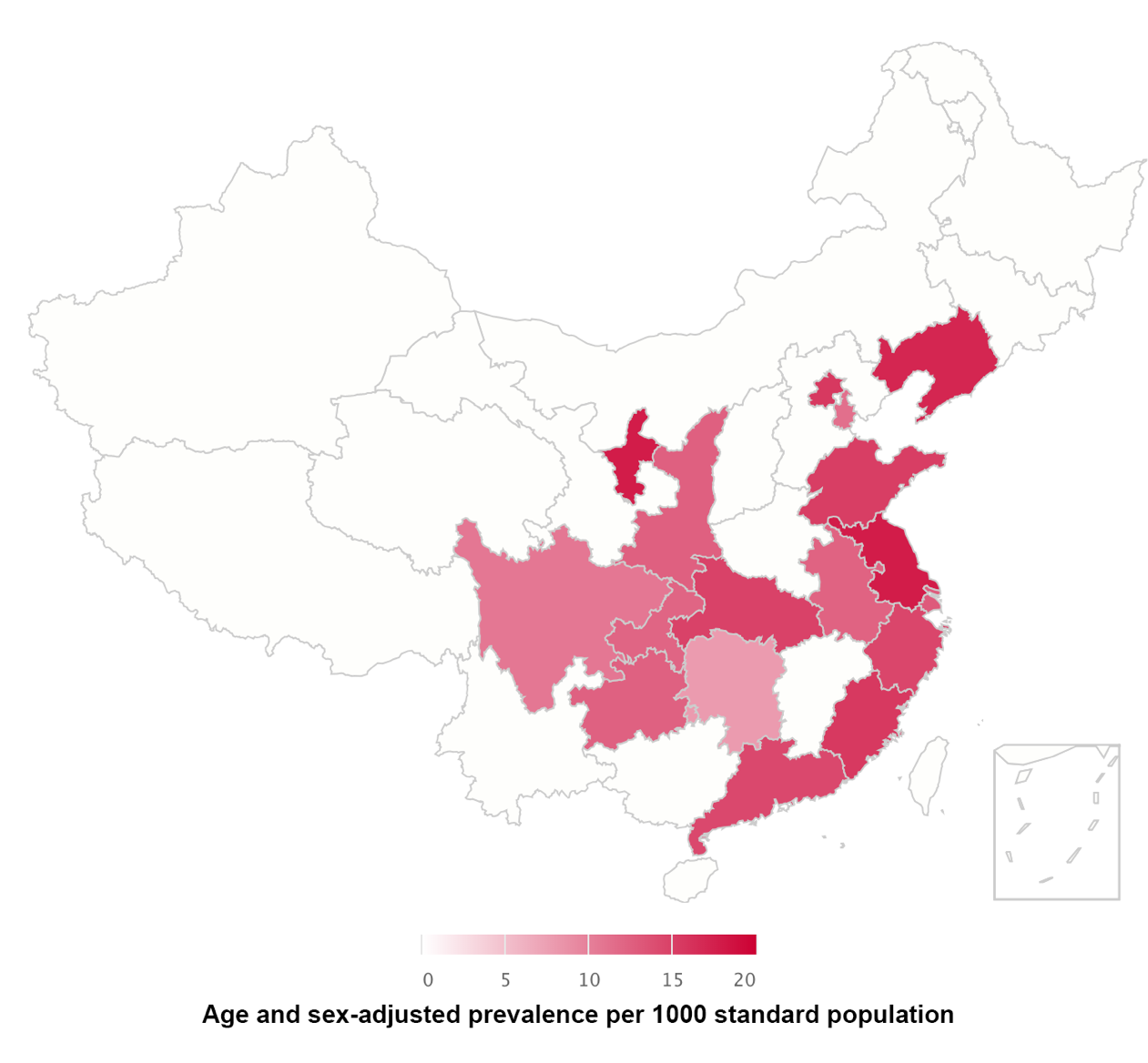


# Figure S4. Prevalence of referral possible glaucoma in 2021 (age and sex-adjusted prevalence per 1000 standard population.


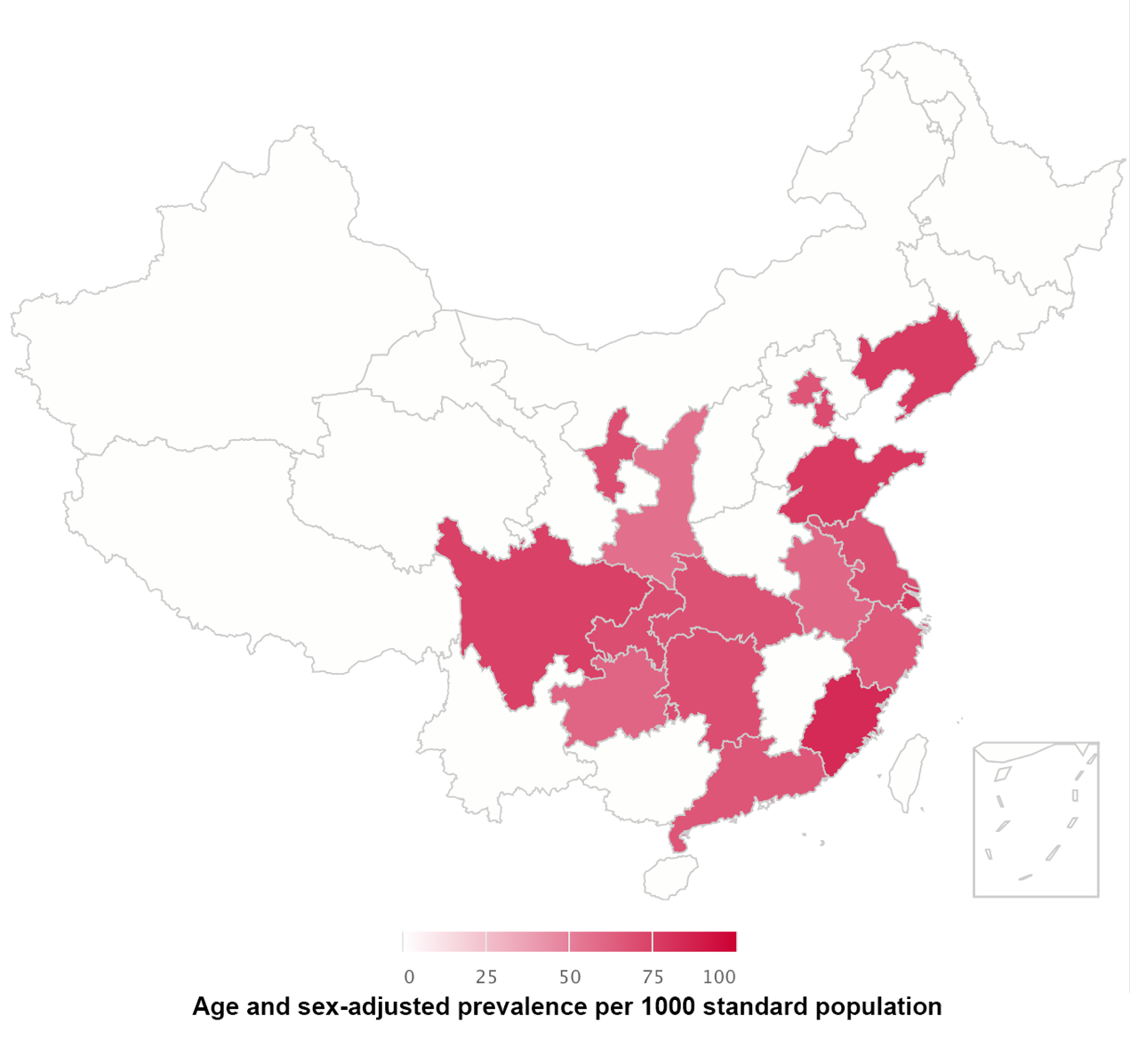


# Figure S5. Prevalence of pathological myopia in 2021(age and sex-adjusted prevalence per 1000 standard population).


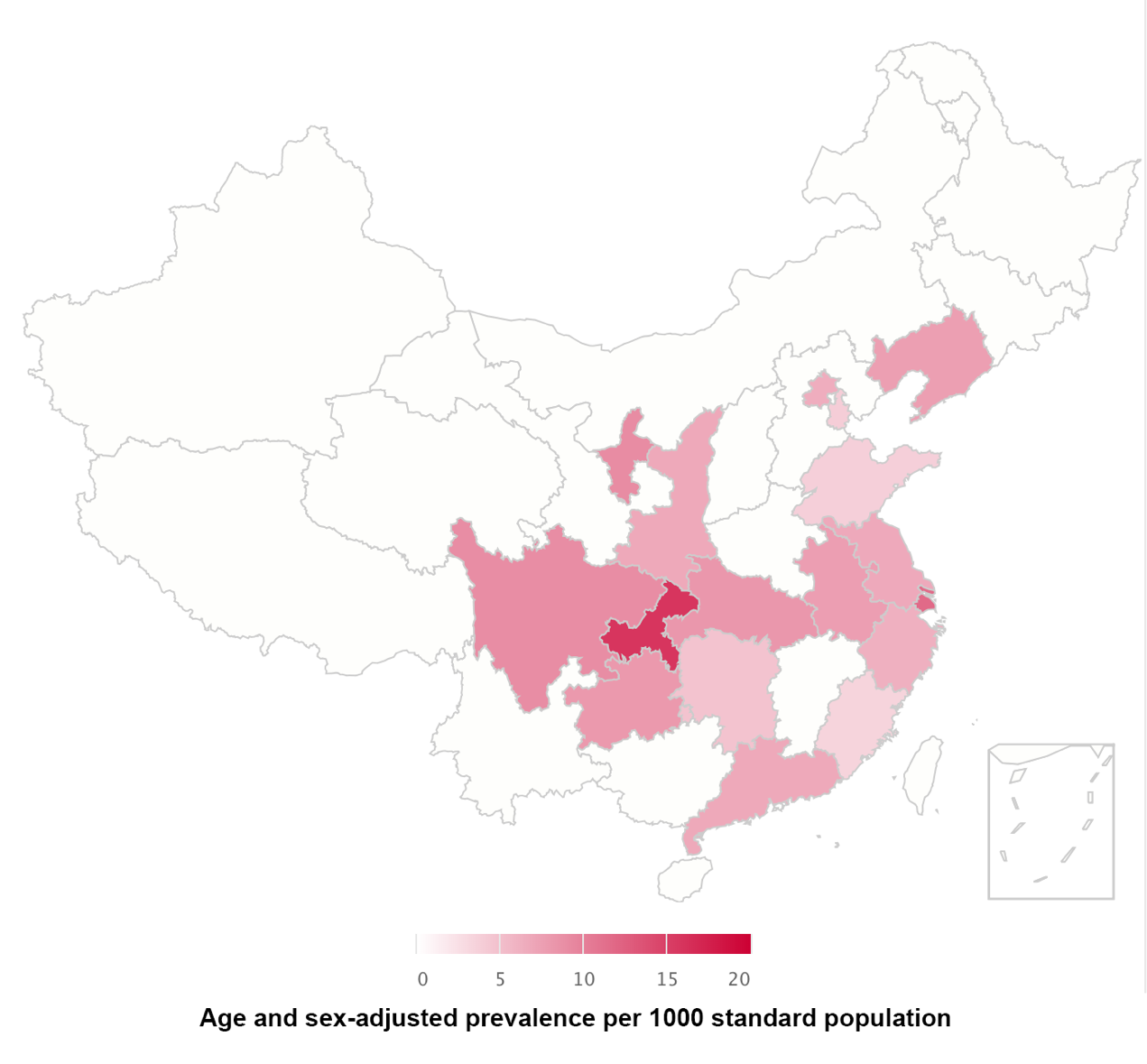


# Figure S6. Prevalence of retinal vein occlusion in 2021(age and sex-adjusted prevalence per 1000 standard population).


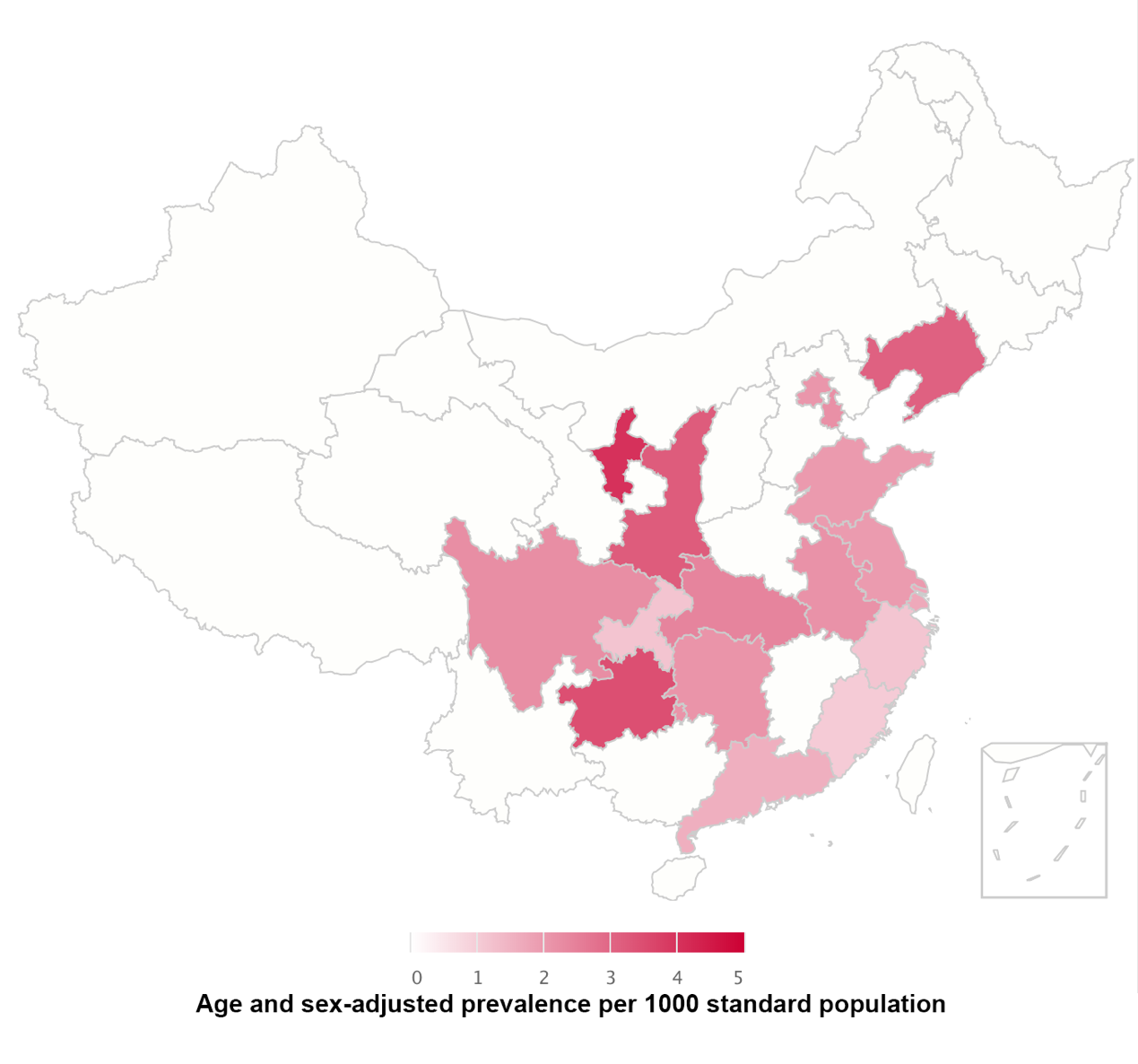


# Figure S7. Prevalence of macula hole in 2021 (age and sex-adjusted prevalence per 1000 standard population).


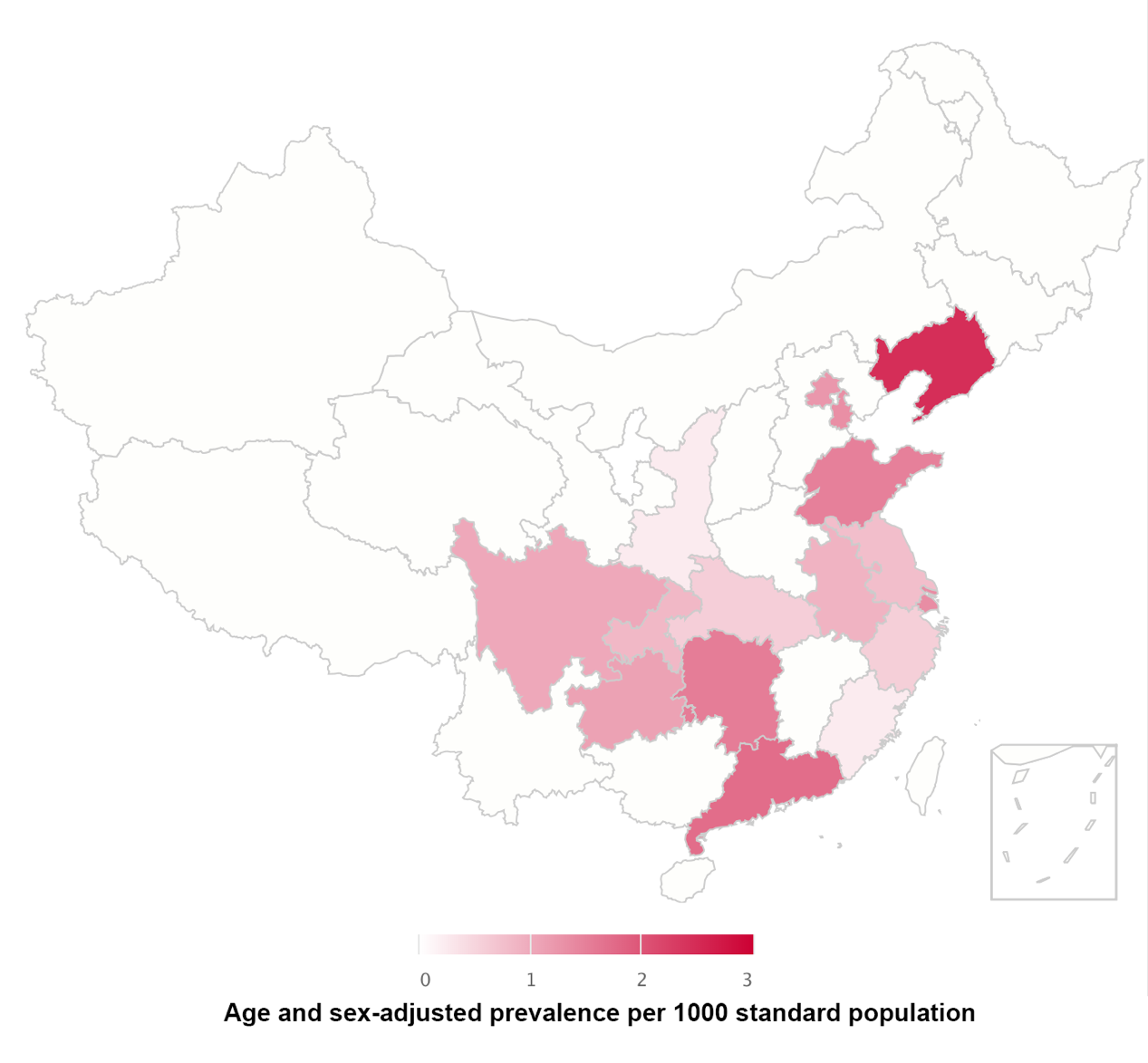


# Figure S8. Prevalence of epiretinal macular membrane in 2021 (age and sex-adjusted prevalence per 1000 standard population).


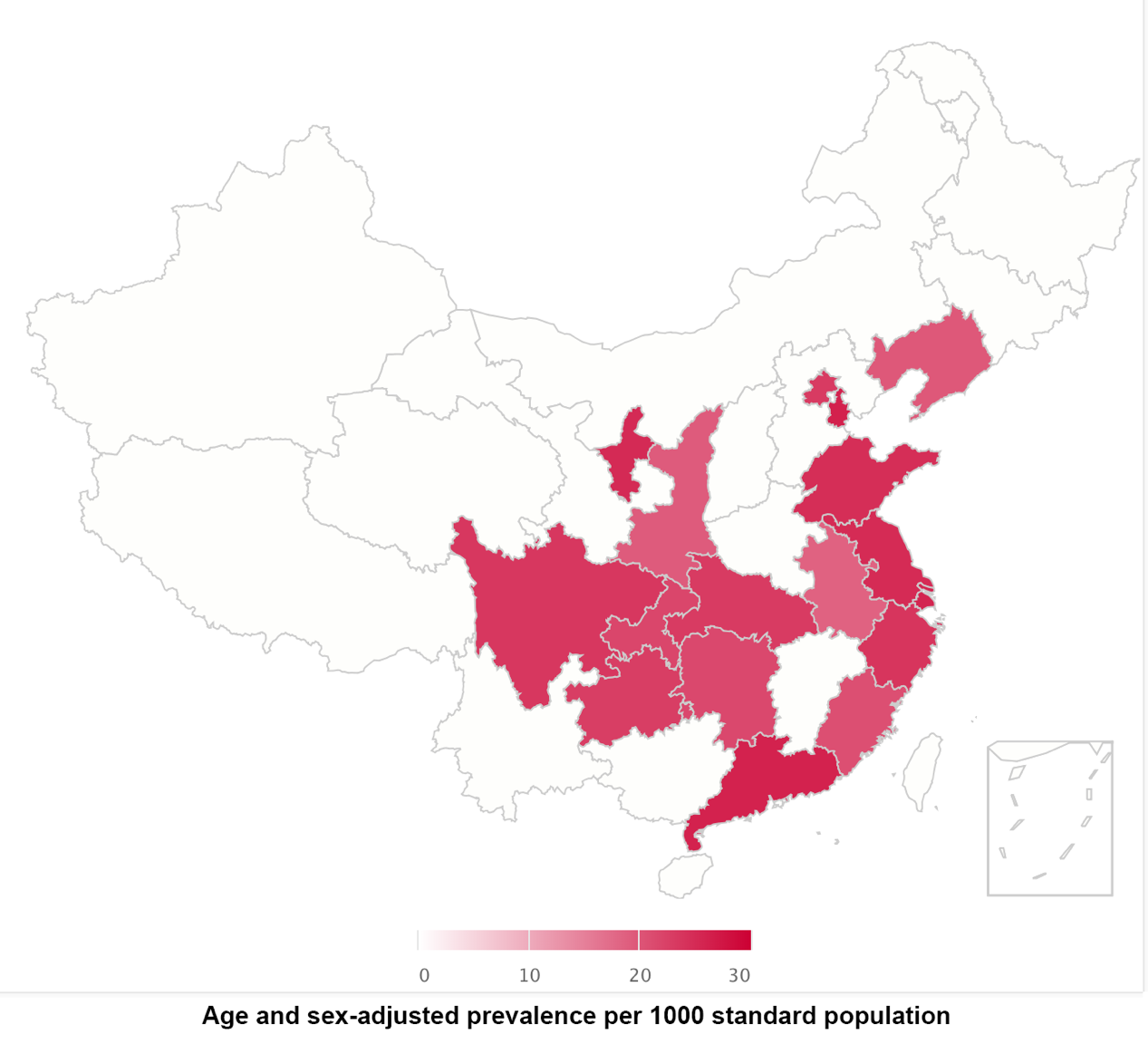


# Figure S9. Prevalence of hypertensive retinopathy in 2021 (age and sex-adjusted prevalence per 1000 standard population).


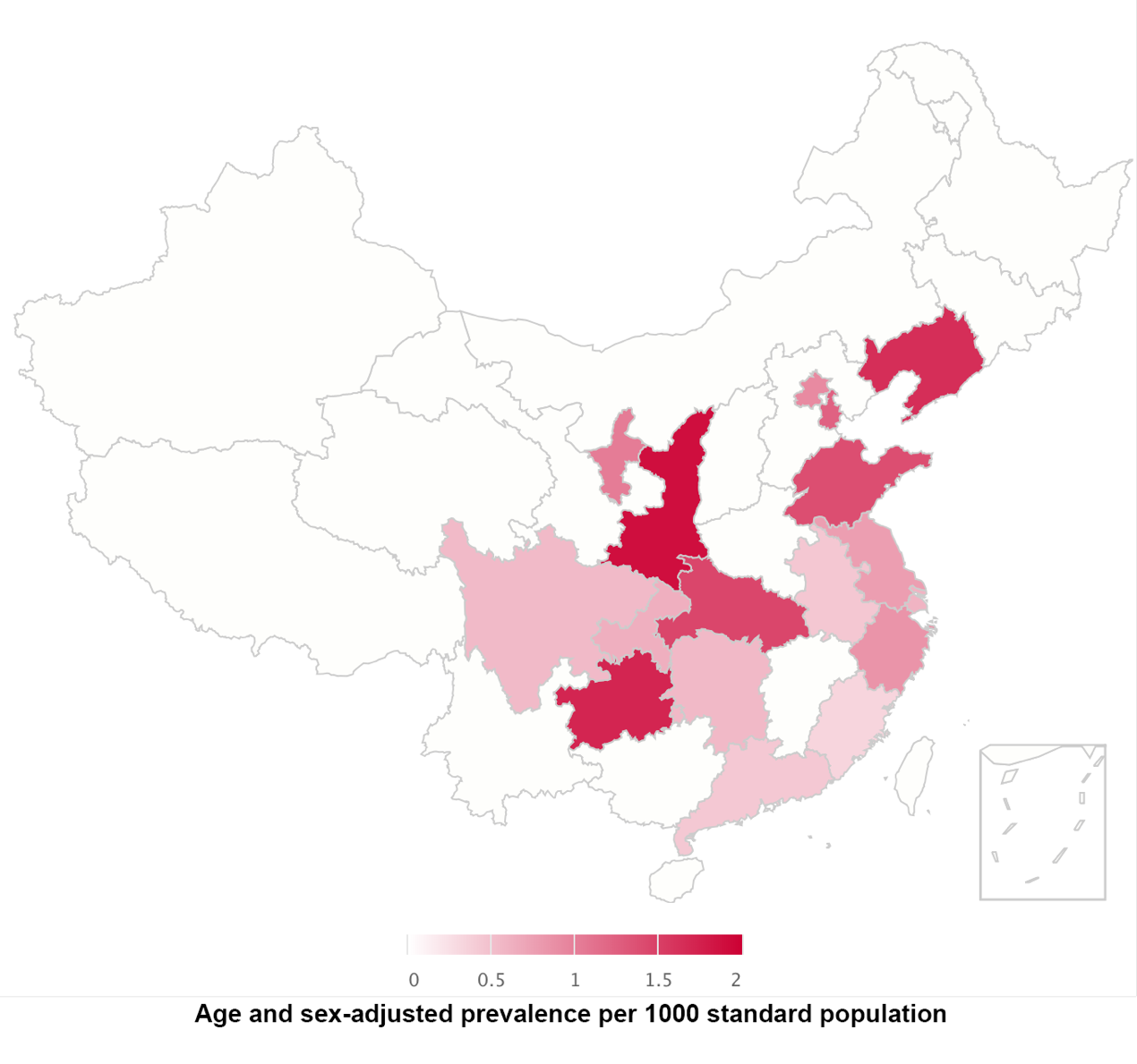


# Figure S10. Prevalence of myelinated fibers in 2021 (age and sex-adjusted prevalence per 1000 standard population).


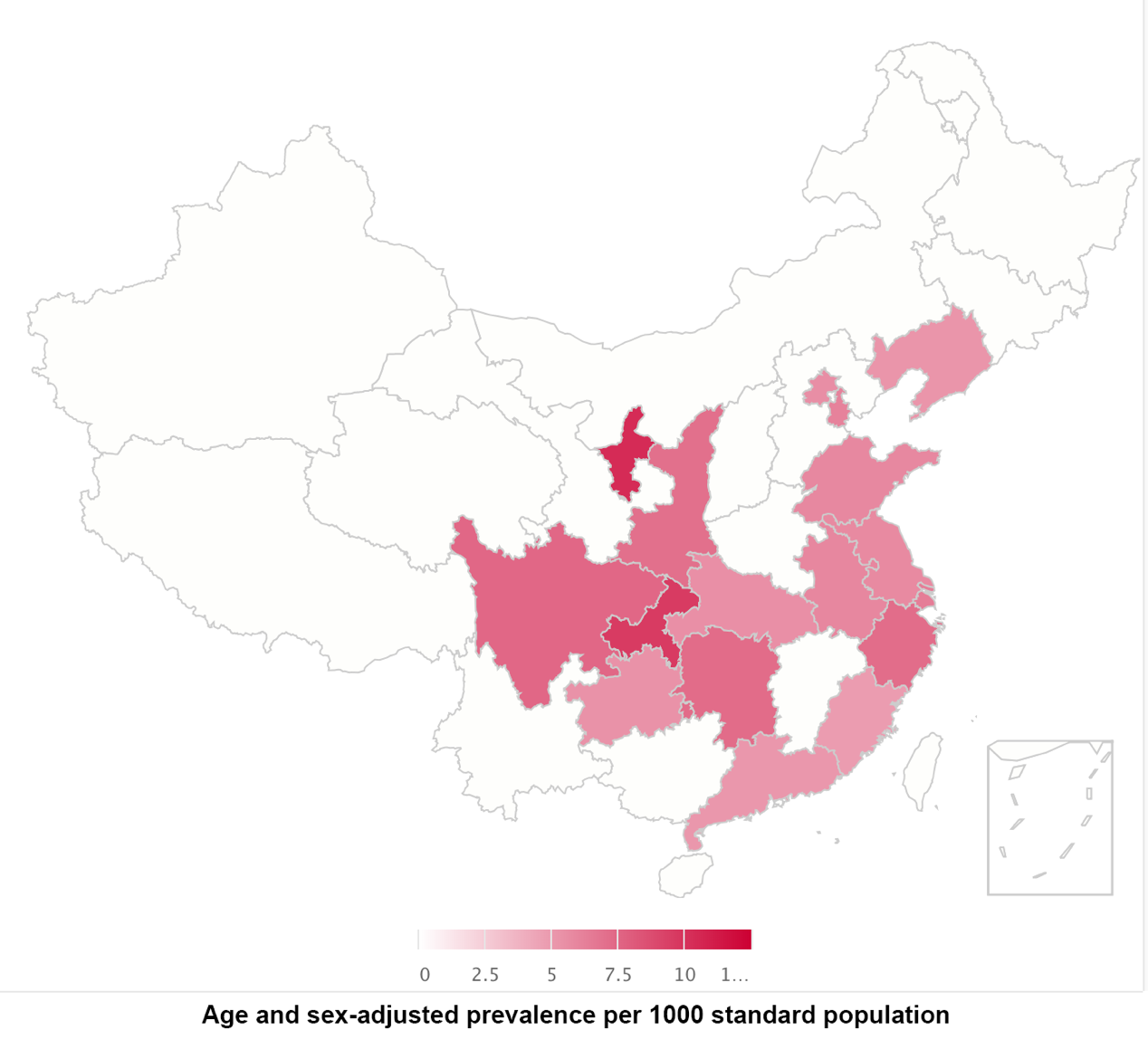


# Figure S11. Prevalence of retinitis pigmentosa in 2021


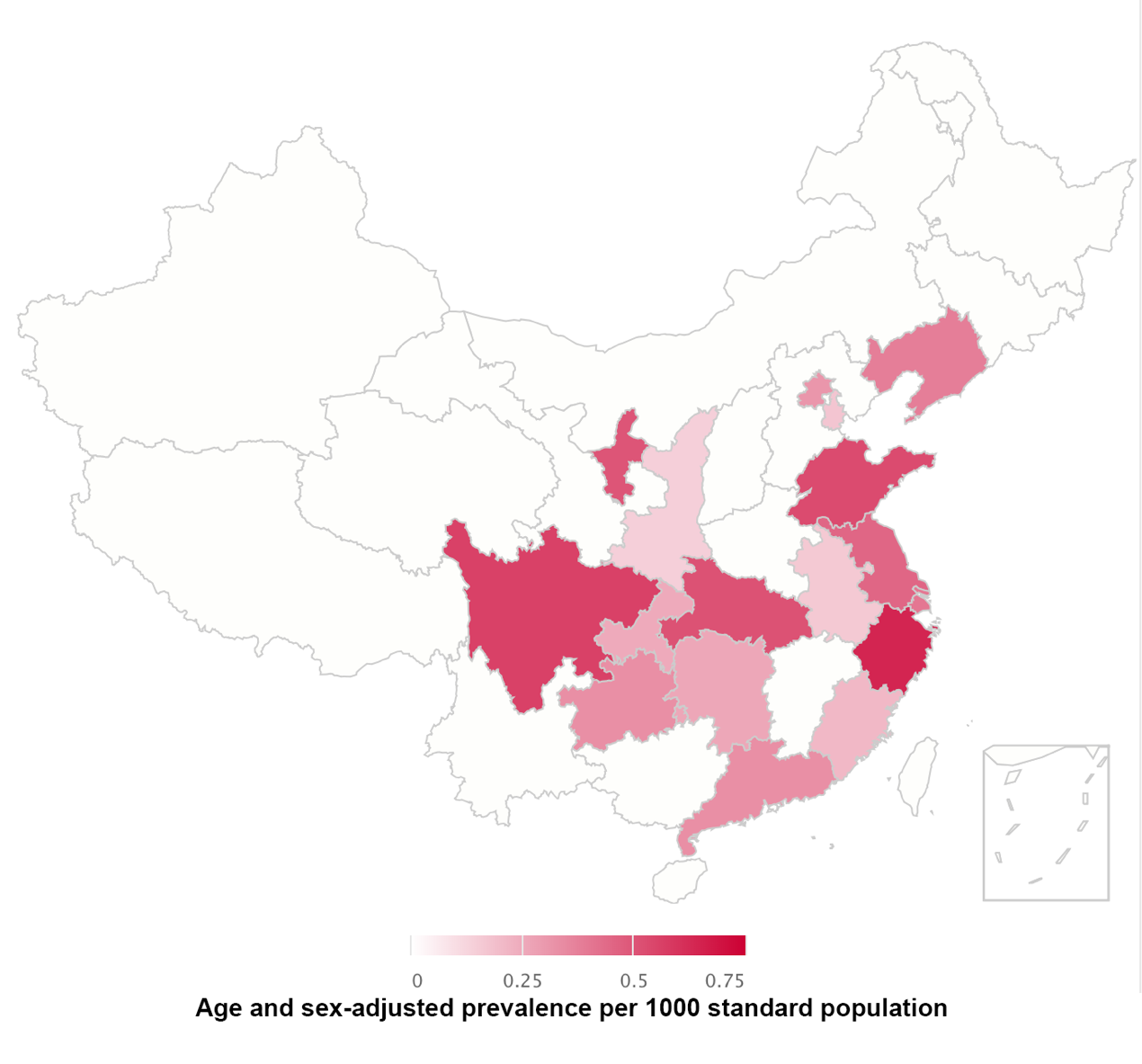

Supplement: Supplement 1 [file tvst-13-4-28_s001.docx]
